# Pushing the Thermodynamic and Kinetic Limits of Near-Infrared Emissive Cr^III^ Complexes in Photocatalysis

**DOI:** 10.1021/jacs.5c08541

**Published:** 2025-07-28

**Authors:** Giacomo Morselli, Tim H. Eggenweiler, Marco Villa, Alessandro Prescimone, Oliver S. Wenger

**Affiliations:** † Department of Chemistry, 27209 University of Basel, St. Johanns-Ring 19, 4056 Basel, Switzerland; ‡ Department of Chemistry “G. Ciamician”, 9296 University of Bologna, Via Piero Gobetti 85, 40129 Bologna, Italy; § Department of Chemistry, University of Basel, BPR 1096, Mattenstrasse 24a, 4058 Basel, Switzerland

## Abstract

Photoactive Cr^III^ complexes are typically based on polypyridine coordination environments, exhibit red luminescence, and are good photo-oxidants but have modest photoreducing properties. We report new Cr^III^ complexes with anionic chelate ligands that enable color-tunable near-infrared luminescence and red-light-driven photoreduction reactions involving elementary steps that are endergonic up to 0.5 eV. Improving the metal–ligand bond covalency rather than more established approaches such as optimizing ligand field strength and coordination geometry is the underlying molecular design concept to achieve this favorable behavior. Our analysis suggests an intricate interplay between productive but slow endergonic photoinduced electron transfer and energy-wasting charge recombination rooted in cage escape effects, which could be generally important for photocatalysis. Our work also suggests the occurrence of doublet–doublet annihilation, a process that seems to have been largely neglected in current research on photoactive Cr^III^ complexes but which could provide a mechanistic entry point into the widely used process of photochemical upconversion, typically based on triplet–triplet annihilation. Overall, this work conceptually advances the current state of the art of photoactive Cr^III^ complexes in terms of molecular design, luminescence, and photoredox behavior. More generally, it informs photochemistry in terms of elucidating the limits of light-to-chemical energy conversion efficiency and the value of long-lived excited states in complexes of earth-abundant transition metals.

## Graphical abstract

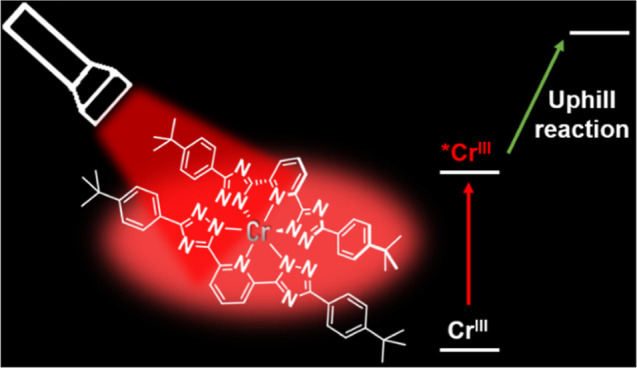

## Introduction

Research on molecular coordination complexes for luminescence and photoinduced electron and energy transfer has focused on second- and third-row transition metals for decades, but the first-row transition metal Cr^III^ was recognized as an interesting alternative, nearly 50 years ago.^1^ With the recent surge of interest in replacing rare and precious metal elements with more abundant ones, Cr^III^ complexes have experienced a revival that was initiated by the development of the so-called molecular rubies,^2^ which are made of new, bite-angle-optimized variants of the well-known polypyridine family of chelate ligands.^3,4^ Molecular rubies exhibit greatly improved photoluminescence properties and extended excited state lifetimes, but their 6-fold pyridine ligand environments are analogous to those used in some of the early Cr^III^ examples.^5^ This limitation in molecular design limits the photophysical and photochemical properties, exemplified by the finding that most Cr^III^ polypyridines luminesce in a narrow wavelength range between 720 and 780 nm and that most of them are strong photo-oxidants but weak photoreductants.^6,7^

We have shown that a fundamentally different molecular design based on anionic ligands allows for near-infrared (NIR) Cr^III^ luminescence beyond 1000 nm and drastically altered electronic structures that are promising for photochemical applications.^6^ Our approach was conceptually different from the previous strategies because it focused on tuning the metal–ligand bond covalency rather than on maximizing the ligand field strength. Several subsequent studies adopted our conceptual approach and reported Cr^III^ complexes emitting in the near-infrared spectral region, many with better properties than we originally reported.^8–10^ Recent work on complexes with other first-row transition metals has provided further evidence for the importance of metal–ligand bond covalency in the molecular design of photoactive complexes.^11,12^

It now seems clear that both design approaches, the traditional approach of creating strong ligand fields and the less conventional approach of optimizing metal–ligand bond covalency, have their inherent limitations. The two approaches are in principle complementary and would ideally be applied simultaneously, but the molecular design factors that affect the ligand field strength usually also affect the metal–ligand bond covalency, and vice versa. Consequently, it is very difficult to optimize everything at once, but we reasoned that work in this direction likely needs to explore a larger chemical space than the well-established polypyridine coordination environments for Cr^III^. For other first-row transition metals, the evolution to carbene and isocyanide ligands has led to conceptual advances that would have been very difficult to achieve with polypyridines, for example with Cr^0^,^13^ Mn^IV^,^14,15^ Fe^II/III^,^16,17^ Co^III^,^18^ and Ni^II^.^19,20^

With this in mind and with an eye toward more exploratory and perhaps drastic changes in the electronic structures and properties of photoactive Cr^III^ complexes, we identified the tridentate bis (1*H*-1,2,4-triazol-5-yl)­pyridine (^R^BTP) ligand framework as potentially interesting (Figure 1c,d).^21–23^ Compared to benchmark molecular rubies (Figure 1a,b),^2,24^ our Cr^III^ complexes exhibit emissions with band maxima (λ_em_) in the near-infrared spectral region beyond 800 nm, and they are significantly more electron-rich, as manifested by their one-electron reduction potentials (*E*_red_) comparable to that of [Ru­(bpy)_3_]^2+^ (bpy = 2,2′-bipyridine). Our [Cr­(^CF3^BTP)_2_]^−^ and [Cr­(^*t*BuPh^BTP)_2_]^−^ complexes in Figure 1c,d represent an attempt to explore uncharted territory between the electron-deficient, strongly red-emissive molecular rubies (Figure 1a,b) and the very electron-rich but nonluminescent Cr^III^ complexes (Figure 1e).^25^

**Figure 1:**
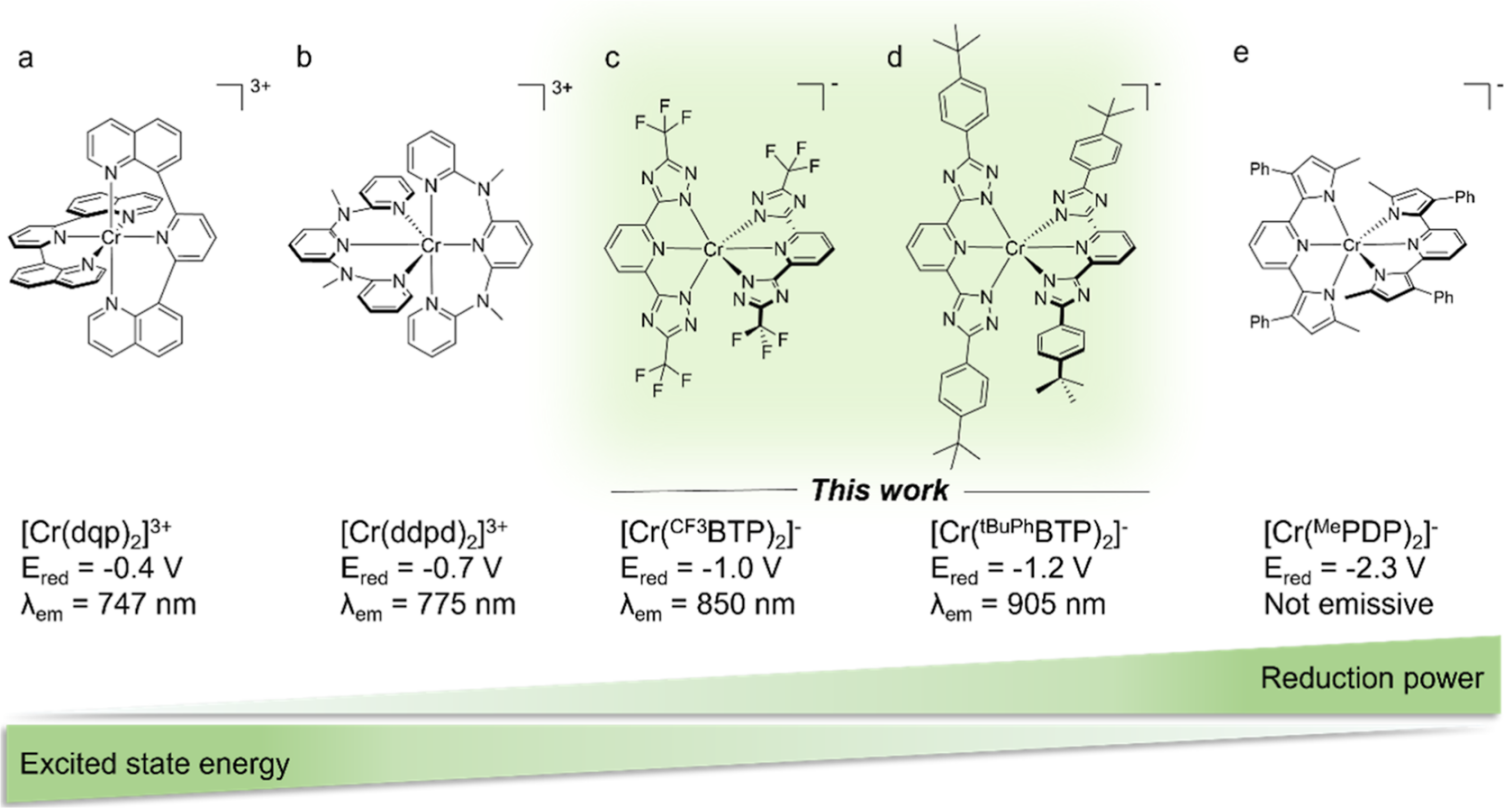
Molecular structures and key electrochemical and photophysical properties of relevant published and new Cr^III^ complexes. The electron-deficient and strongly red luminescent (a) [Cr­(dqp)_2_]^3+^ ^24^ and (b) [Cr­(ddpd)_2_]^3+^, for which ligand field strength and optimized coordination geometry were key design criteria.^2^ The new electron-rich near-infrared luminescent [Cr­(^R^BTP)_2_]^−^ complexes (c,d), with greater metal–ligand bond covalency as a key molecular design concept (R = CF_3_ or *t*BuPh). Nonluminescent [Cr­(^Me^PDP)_2_]^−^ complex (e).^25^ *E*_red_ is the electrochemical potential for one-electron reduction in V vs SCE, λ_em_ is the emission band maximum in solution at room temperature.

Our molecular design allows for color-tunable near-infrared luminescence at room temperature due to a balance between ligand field strength and metal–ligand bond covalency^26^ and also provides access to an unusual photochemistry for Cr^III^ complexes, where photoreduction reactions become possible instead of the more commonly studied photo-oxidations.^7,27–29^ This molecular design also represents a compromise between lowered excited state energies and consequent accelerated (nonradiative) excited state decay, a common dilemma in photophysics.^26,30^ The compromise achieved here makes photoinduced electron transfer steps that are endergonic up to 0.5 eV kinetically competitive with the inherent excited state decay, allowing us to explore and extend the current kinetic and thermodynamic limits of Cr^III^ photochemistry. Based on the results obtained, we gain insight into the trade-offs between slow, endergonic, and fast, exergonic photoinduced electron transfer elementary steps, allowing us to discuss generally relevant design concepts for photochemistry to optimize the interplay between energy-storing and energy-wasting processes. We also observe evidence of second-order processes that arise from either doublet–doublet annihilation or excited-state disproportionation. These possibilities have been largely overlooked in modern Cr^III^ photophysics and photochemistry, possibly due to the electrostatic repulsion between the highly charged Cr^III^ complexes typically studied. While these processes are currently energy-wasting, they hold the potential to be harnessed for efficient light conversion in the future.^31^

## Results and Discussion

### Synthesis and Structural Aspects of Anionic Complexes with a High Metal–Ligand Binding Covalency

The ^R^BTP ligands were synthesized through straightforward multistep procedures (Figure 2a).^21–23^ The reaction between pyridine-2,6-dicarbonitrile and hydrazine yielded a bis­(carboximidhydrazide), which then underwent amide coupling in the presence of trifluoroacetic acid, 4-*tert*-butylbenzoyl chloride, or 4-*tert*-butylbenzoic acid. Subsequent dehydration and cyclization at high temperatures produced trifluoromethyl (^CF3^BTP) and (*tert*-butyl)­phenyl (^*t*BuPh^BTP) derivatives, respectively. The Cr^III^ complexes (hereby abbreviated as [Cr­(^R^BTP)_2_]^−^) were prepared by first deprotonating the BTP ligands, followed by chelation of Cr^II^ and oxidation under air. The direct use of Cr^III^ salts (i.e., CrCl_3_·3THF) proved ineffective. Cation exchange with tetra-*n*-butylammonium (*n*Bu_4_N^+^) ensured solubility of the complexes in organic solvents such as DCM and MeCN, yielding (*n*Bu_4_N)­[Cr­(^CF3^BTP)_2_] and (*n*Bu_4_N)­[Cr­(^*t*BuPh^BTP)_2_] (Figure 2b).

**Figure 2:**
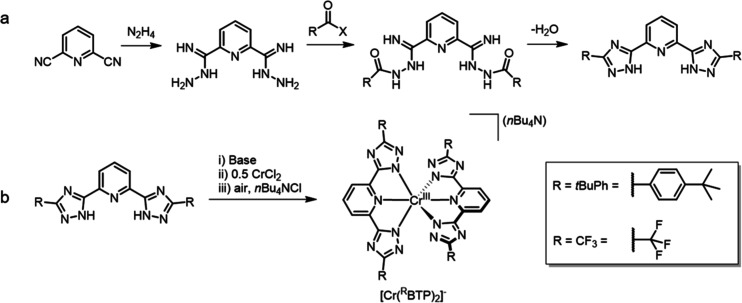
(a) Synthesis of the bis­(1*H*-1,2,4-triazol-5-yl)­pyridine (^R^BTP) ligands, where R is either a (*tert*-butyl)­phenyl (*t*BuPh) or a trifluoromethyl (CF_3_) group. X = Cl or OH in the case of R = *t*BuPh, and X = OH in the case of R = CF_3_. (b) Synthesis of the [Cr­(^R^BTP)_2_]^−^ complexes.

The single-crystal X-ray structures of [Cr­(^CF3^BTP)_2_]^−^ (Figure 3a) and [Cr­(^*t*BuPh^BTP)_2_]^−^ (Figure 3b) confirm a distorted octahedral coordination geometry. For [Cr­(^CF3^BTP)_2_]^−^, the average bond distance between the metal and the coordinating nitrogen atoms is 2.029 Å, with the Cr^III^–N bonds of the anionic triazole groups being almost the same length as the axial Cr^III^–N bonds of the pyridine units. On the other hand, for [Cr­(^*t*BuPh^BTP)_2_]^−^, the average bond distance between Cr^III^ and the anionic triazole groups is 2.047 Å, which is 0.04 Å longer than the axial Cr^III^–N bonds of the pyridine units. This difference is evident in the computed parameter Δ (eq S1), which accounts for the average deviation in ligand–metal bond lengths.^32^ Δ is only 1.8 × 10^–5^ for [Cr­(^CF3^BTP)_2_]^−^ but it increases to 2.8 × 10^–4^ in [Cr­(^*t*BuPh^BTP)_2_]^−^. For comparison, the Cr–N bond distances in [Cr­(bpy)_3_]^3+^ (bpy = 2,2′-bipyridine) and [Cr­(NH_3_)_6_]^3+^ lie in the range of 2.04–2.05 Å^33^ and 2.06 Å,^34^ respectively, and the Δ parameters of these complexes are 5.6 × 10^–5^ and 2.8 × 10^–5^. An even more pronounced distortion from the ideal octahedral geometry is observed when considering the bond angles. The angles formed between the pyridine nitrogen (N5), the metal center, and the triazole nitrogen (N4) of the same ligand are 77.4° for [Cr­(^CF3^BTP)_2_]^−^ and 77.8° for [Cr­(^*t*BuPh^BTP)_2_]^−^. When the triazole nitrogens from the two different coordinated ligands are considered (N3 instead of N4), the N5–Cr^III^–N3 angle measures around 102° for both complexes. These values deviate significantly from the ideal angle found in the nearly perfectly octahedrally shaped [Cr­(NH_3_)_6_]^3+^ complex (89.7°).^34^ Closer similarities are observed in [Cr­(tpy)_2_]^3+^ (tpy = 2,2′:6′,2″-terpyridine), where the chelate angle between adjacent pyridines and the metal center falls within the 77.4–79.59° range.^35,36^ The distortion from the ideal 90° *cis* bond angles is captured by the parameter Σ (eq S2), which is approximately 107° for [Cr­(^CF3^BTP)_2_]^−^, 114° for [Cr­(^*t*BuPh^BTP)_2_]^−^, and 122° for [Cr­(tpy)_2_]^3+^, but only 18°^37^ for [Cr­(NH_3_)_6_]^3+^ and 67°^33^ for [Cr­(bpy)_3_]^3+^ (see Table S3).

**Figure 3:**
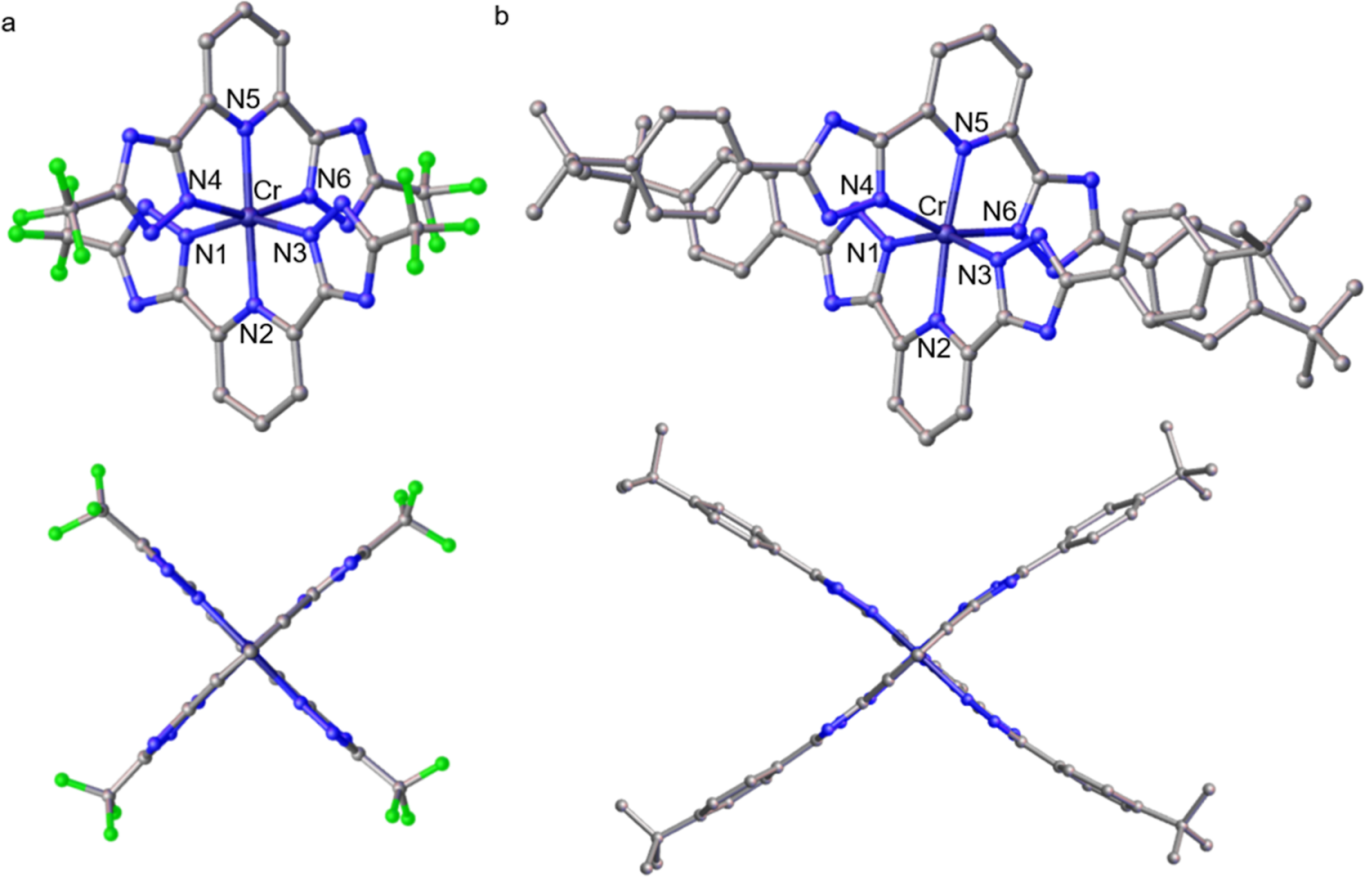
Solid-state X-ray crystal structure of (a) [Cr­(^CF3^BTP)_2_]^−^ and (b) [Cr­(^*t*BuPh^BTP)_2_]^−^ viewed from two different angles. Disordered atoms, hydrogen atoms, and solvent molecules have been omitted for clarity.

The distorted geometries of our new complexes are furthermore reflected in the octahedral distortion parameter Θ (eq S3), which is equal to 357° and 369° for the CF_3_ and the *t*BuPh derivatives, respectively, falling within the typical range for bis­(tridentate) complexes.^32^ For example, [Cr­(tpy)_2_]^3+^ shows a Θ value of 370°^36^ (see Supporting Information for further details). By contrast, [Cr­(dqp)_2_]^3+^ and [Cr­(ddpd)_2_]^3+^ exhibit lower Θ values of 105°^24^ and 122°,^38^ respectively, due to the greater flexibility of the ligands that allows for an improved octahedral symmetry. Monodentate complexes, which display the least distorted geometries, usually exhibit Θ values in the range of 50–75°.^32^ The N2–Cr–N5 angles of [Cr­(^CF3^BTP)_2_]^−^ and [Cr­(^*t*BuPh^BTP)_2_]^−^, which involve the apical pyridine nitrogens, are 178° and 174°, respectively, both close to the ideal linear arrangement. The dihedral angle between the planes of the two ligands in [Cr­(^CF3^BTP)_2_]^−^ is close to 90°. [Cr­(^*t*BuPh^BTP)_2_]^−^ exhibits deviations of up to 8% from a perfect right angle, and the ligands do not lie in perfectly defined planes, corroborating a more distorted geometry. Notably, the cross-shaped structure of both complexes provides limited shielding of the central Cr^III^ ion despite the steric bulk of the ligands.

### Shifting the Luminescence from the Red to the Near-Infrared Range and Fine-Tuning the Energy of the Photoactive Excited State

For Cr^III^ in coordination environments that can be approximated as octahedral and in strong ligand fields, the lowest electronically excited state is one in which an electron spin has been flipped, but the orbital occupancy is otherwise the same as in the ground state. The energy of this spin-flip excited state is independent of the ligand field strength,^39^ and therefore, conventional molecular design concepts are insufficient to tune Cr^III^ luminescence over a significant wavelength range.^6,26^ The concept of chelate bite angle optimization is highly successful in counteracting unwanted nonradiative relaxation of the emissive spin-flip excited state (in large part due to avoided thermal population of higher excited states from which nonradiative deactivation can easily occur),^2,3,24,40–43^ but to directly influence the radiative spin-flip transition, it is less effective than changing the metal–ligand bond covalency.^6,12^ A more drastic change than optimizing the coordination geometry of a polypyridine environment is therefore needed, even if this comes at the cost of a lower luminescence quantum yield. A classic approach to enhancing the metal–ligand bond covalency is to introduce anionic ligands, which we achieved here by using deprotonated triazole units integrated into a tridentate chelate ligand.

Further tuning of the electronic properties then is possible by varying the ligand’s chemical substituents: the inductive electron-withdrawing effect of CF_3_ leads to a blue shift in both absorption and emission bands compared to the more conjugated *tert*-butylphenyl (*t*BuPh) substituent. Both complexes absorb only weakly in the visible range, and the strongest bands are due to ligand-centered (LC) transitions occurring in the UV range (Figure 4a,b, solid traces). [Cr­(^*t*BuPh^BTP)_2_]^−^ (red traces) shows an absorption band centered at 498 nm with a strong MC character according to DFT calculations and a band centered at 422 nm with mixed LC, charge-transfer (CT), and MC character. Similarly, [Cr­(^CF3^BTP)_2_]^−^ (blue traces) has absorption bands between 425 and 452 that have a strong MC character. In the spectrum of [Cr­(^*t*BuPh^BTP)_2_]^−^ a tailing of the absorption up to 700 nm is furthermore detectable (inset of Figure 4b), which is likely attributable to spin-forbidden LC transitions.^24^

**Figure 4:**
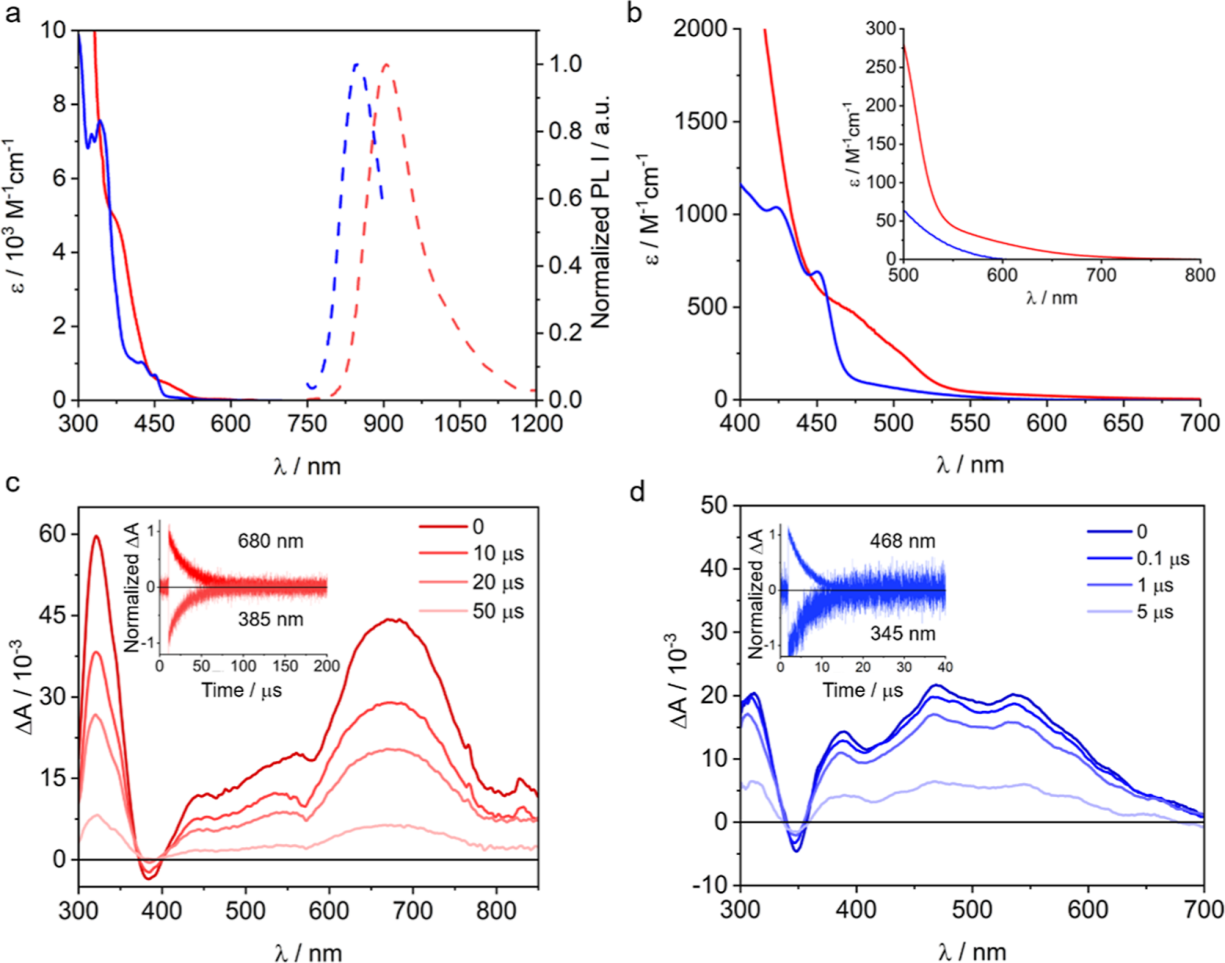
(a) UV–vis absorption (solid traces) and emission (dashed traces) spectra of (*n*Bu_4_N)­[Cr­(^CF3^BTP)_2_] (blue, λ_ex_ = 350 nm) and (*n*Bu_4_N)­[Cr­(^*t*BuPh^BTP)_2_] (red, λ_ex_ = 300 nm). (b) Magnification of the absorption spectra between 400 and 800 nm. (c) Transient UV–vis absorption spectra at different delay times for [Cr­(^*t*BuPh^BTP)_2_]^−^ (40 μM, laser pump pulses at 355 nm, 30 mJ/pulse, and integration time of 1.5 μs). Inset: decay of the excited-state absorption (ESA) at 680 nm and recovery of the ground state bleach (GSB) at 385 nm. (d) Transient UV–vis absorption spectra at different delay times for [Cr­(^CF3^BTP)_2_]^−^ (40 μM, laser pump pulses at 355 nm, 30 mJ/pulse, integration time of 1.5 μs). Inset: decay of the ESA at 468 nm and recovery of the GSB at 345 nm. All measurements were performed in deoxygenated acetonitrile at room temperature.

Both complexes emit in the near-infrared (NIR) range in solution at room temperature (Figure 4a, dashed traces), with [Cr­(^CF3^BTP)_2_]^−^ displaying a maximum emission at 850 nm, while [Cr­(^*t*BuPh^BTP)_2_]^−^ emits at 905 nm, in both cases attributable to phosphorescence from the ^2^E and ^2^T_1_ states.^44^ Since it is difficult to assess the real nature of the emissive state,^45^ we will refer to both of them from now on. Moreover, the emission bandwidth (full width at half-maximum, fwhm, of 1900 cm^–1^) is atypical of spin-flip transitions, suggesting state mixing also with the ligand, as observed for other Cr^III^ complexes with strong metal–ligand bond covalency.^6,46^ Due to these comparably broad ^2^E/^2^T_1_ → ^4^A_2_ emission bands, the energy of the emissive doublet excited states can be underestimated if computed at the maxima. Therefore, we estimated the ^2^E/^2^T_1_ energies from the point on the short wavelength side of the emission spectra at which the intensity corresponds to 10% of the intensity reached at the maxima. This analysis yields 780 nm (corresponding to an energy of 1.59 eV for [Cr­(^CF3^BTP)_2_]^−^) and 820 nm (1.50 eV for [Cr­(^*t*BuPh^BTP)_2_]^−^), respectively. At 77 K, the luminescence of both complexes is blue-shifted by about 20–30 nm relative to 298 K (Figure S17), as reported earlier for other chromium­(III) complexes.^8^ Technical limitations precluded the determination of the photoluminescence quantum yield of [Cr­(^CF3^BTP)_2_]^−^, whereas for [Cr­(^*t*BuPh^BTP)_2_]^−^ we determined a photoluminescence quantum yield of (0.10 ± 0.01)% in deoxygenated acetonitrile at room temperature (see Supporting Information for further details). For both complexes, the excitation spectra monitoring the NIR photoluminescence closely resemble the UV–vis absorption spectra (Figures S15 and S16), indicating near-unitary efficiency of photoactive state population upon absorption across all investigated wavelengths.

Evidently, the different chemical substituents on the triazole units have a substantial effect on the electron density at the Cr^III^ center. This is noteworthy given previous findings that triazoles can exhibit a somewhat “insulating” character with respect to long-range electron transfer and can influence charge-transfer dynamics.^47,48^ This opens up possibilities for exploring a broad range of other substitution patterns to fine-tune the properties of near-infrared emissive Cr^III^ complexes, akin to strategies previously employed for photoactive complexes based on precious metals.^49–52^

Transient UV–vis absorption spectroscopy with nanosecond time resolution in deoxygenated acetonitrile revealed excited state absorptions (ESAs) over the entire visible range for both complexes, which were attributed to electronic transitions originating from the luminescent ^2^E/^2^T_1_ excited state (Figure 4c,d). For [Cr­(^*t*BuPh^BTP)_2_]^−^, an ESA band maximum at 680 nm and a ground state bleaching (GSB) at 385 nm are the most prominent spectral features (Figure 4c), while for [Cr­(^CF3^BTP)_2_]^−^ an ESA band at 468 nm and a GSB at 345 nm are prominent (Figure 4d). Monitoring these ESA and GSB signals as a function of time yields excited state lifetimes of 23 μs for [Cr­(^*t*BuPh^BTP)_2_]^−^ and 5 μs for [Cr­(^CF3^BTP)_2_]^−^.

### Balancing the Ligand Field Strength and the Metal–Ligand Bond Covalency

The electronic structures of octahedral d-metal complexes can be rationalized by the interplay between the ligand field strength (described by the ligand field parameter 10 Dq) and the metal–ligand bond covalency (captured by the Racah *B* parameter). The Tanabe–Sugano diagrams illustrate that interplay for all d-electron configurations, and the relevant d^3^ case for Cr^III^ is shown in Figure 5a. Some of the best Cr^III^ red luminophores based on coordination geometry optimized polypyridines have 10 Dq values near 25,000 cm^–1^ and *B* values around 660 cm^–1^, leading to a 10 Dq/*B* ratio near 38 in the specific case of [Cr­(dqp)_2_]^3+^ (dqp = 2,6-bis­(8′-quinolinyl)­pyridine).^24^ The deprotonated triazoles of our ^R^BTP chelates are π-donor ligands and are therefore expected to lead to a decrease in the 10 Dq value compared to polypyridine ligands.^11^ This in turn is expected to result in an energetic stabilization of the metal-centered ^4^T_2_ state (brown line in Figure 5a), which arises from an electronic transition from a t_2_ to an e orbital (Figure 5b, exemplary microstates on the far right), whereas the spin-flip ^2^E/^2^T_1_ excited state is expected to remain relatively unaffected by the decrease in 10 Dq.

**Figure 5:**
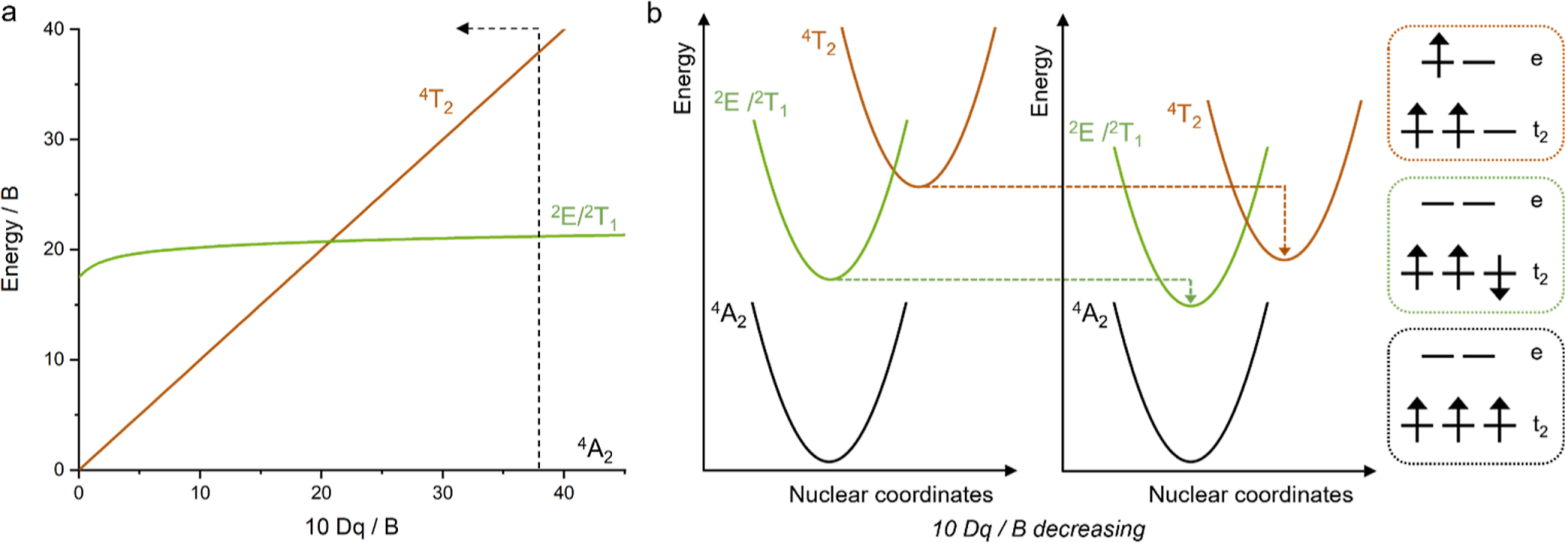
(a) Simplified Tanabe–Sugano diagram for an octahedral d^3^ configuration, with the ^2^E/^2^T_1_ spin-flip excited states (green) and the ligand field-dependent ^4^T_2_ excited state (brown) included, while all other states are not shown. The dashed vertical line represents the case of [Cr­(dqp)_2_]^3+^, for which 10 Dq/*B* = 38.^24^ (b) Resulting shift of the potential energy surfaces of the states involved upon decreasing the 10 Dq/*B* ratio in the presence of π-donor anionic (deprotonated) triazole ligand units. The potential energy surfaces and some relevant microstates are marked in black (^4^A_2_, ground state), green (^2^E, spin-flip state), and brown (^4^T_2_, metal-centered state).

The Racah *B* parameter, which reflects the repulsion between individual d-electrons, decreases in the presence of ligands that form strong covalent bonds, such as amides, carbazolates, pyrrolates, and deprotonated triazoles.^12^ The resulting delocalization of d-electron density from the metal-based orbitals toward the ligands is known as the nephelauxetic effect and is characterized by the nephelauxetic parameter β, which is the ratio of the Racah *B* parameter of a given complex and the *B* value of the free Cr^3+^ ion in the gas phase. A decrease of the Racah *B* parameter can explain why the ^2^E photoluminescence red shifts by 1100–1800 cm^–1^ for [Cr­(^CF3^BTP)_2_]^−^ and [Cr­(^*t*BuPh^BTP)_2_]^−^ with respect to a benchmark molecular ruby.^2^ Based on ^2^E energies of 12,820 cm^–1^ (780 nm) and 12,200 cm^–1^ (820 nm), and assuming a *C*/*B* ratio of 4.0 as in many textbooks (*C* is a second Racah parameter), we obtain *B* parameters of 660 and 630 cm^–1^ for [Cr­(^CF3^BTP)_2_]^−^ and [Cr­(^*t*BuPh^BTP)_2_]^−^, respectively. However, the assumption of *C*/*B* = 4.0 seems somewhat ambiguous in light of recent studies on Co^III^ complexes, which have found *C*/*B* ratios that are considerably higher.^53^ The bottom line is that the resulting estimates of the Racah *B* parameters for our Cr^III^ complexes are subject to considerable uncertainty. The resulting nephelauxetic parameters β are 0.69 and 0.66 for [Cr­(^CF3^BTP)_2_]^−^ and [Cr­(^*t*BuPh^BTP)_2_]^−^, respectively. These values are lower than those of the champion polypyridine complex [Cr­(ddpd)_2_]^3+^ (ddpd = *N*,*N*′-dimethyl-*N*,*N*′-dipyridine-2-yl-2,6-diamine), for which β = 0.80,^2^ indicating the expected greater degree of covalency with the deprotonated triazolate ligands.

Accurate determination of the 10 Dq values in our complexes is likewise challenging due to CT absorption bands that mask the weaker MC transitions and due to the admixture of CT character to some of our MC excited states (see above). Based on the DFT calculations mentioned above, the absorption band at 498 nm observed for [Cr­(^*t*BuPh^BTP)_2_]^−^ (Figure 4a,b) has a strong MC character and is therefore tentatively attributed to the ^4^A_2_ → ^4^T_2_ transition, which then implies a 10 Dq value of about 20,100 cm^–1^. Proceeding analogously for [Cr­(^CF3^BTP)_2_]^−^, we obtain a 10 Dq value of about 22,100 cm^–1^, based on the absorption band at 452 nm (Figure 4a).

The resulting 10 Dq/*B* ratios are 32 for [Cr­(^*t*BuPh^BTP)_2_]^−^ and 33 for [Cr­(^CF3^BTP)_2_]^−^, but these ratios only represent rather rough estimates, since both the determination of 10 Dq and *B* are subject to the limitations described above. Our 10 Dq/*B* estimates are substantially lower than that reported for [Cr­(dqp)_2_]^3+^ (dashed vertical line in Figure 5a and Table 1), suggesting stabilization of the ^4^T_2_ excited state in addition to stabilization of the luminescent ^2^E state. A smaller energy gap between ^2^E and ^4^T_2_ may also contribute to the lower luminescence quantum yield in our [Cr­(^*t*BuPh^BTP)_2_]^−^ complex (0.1%) compared to [Cr­(dqp)_2_]^3+^ (5.2%),^24^ in addition to the smaller energy gap between the ^2^E excited state and the ^4^A_2_ ground state.

**Table 1:** Ligand Field Parameters (10 Dq), Racah Parameters (B) and Nephelauxetic Parameters (β) for Several Photoactive Cr^III^ Complexes

complex	10 Dq/cm^–1^	B/cm^–1^	10 Dq/*B*	β
[Cr(dpc)_2_]^+^ ^a^	19,200	550	35	0.64
[Cr(ddpd)_2_]^3+^ ^b^	22,900	760	30	0.80
[Cr(dqp)_2_]^3+^ ^c^	24,937	660	38	0.69
[Cr(NH_3_)_6_]^3+^ ^d^	21,600	670	32	0.70
[Cr(^CF3^BTP)_2_]^−^	22,100	660	33	0.69
[Cr(^*t*BuPh^BTP)_2_]^−^	20,080	630	32	0.66

a From refs 6 and 12 .

b From ref 3.

c From ref 24.

d From ref 12.

### Setting the Basis for Photoreduction Reactions

Based on cyclic voltammetry, [Cr­(^*t*BuPh^BTP)_2_]^−^ is reduced at a potential of −1.21 V vs SCE, while the more electron-deficient [Cr­(^CF3^BTP)_2_]^−^ is reduced already at −1.00 V vs SCE and also exhibits a second reduction wave centered at −1.53 V vs SCE (Figure 6). The observed trend in reduction potentials is compatible with a ligand-centered reduction in both cases, as previously reported for [Cr­(^Me^PDP)_2_]^−^ (^Me^PDP = 2,6-bis­(5-methyl-3-phenyl-1*H*-pyrrol-2-yl)­pyridine, Figure 1e) and several Cr^III^ polypyridines.^24,25,45^ The reduction potentials of our anionic [Cr­(^*t*BuPh^BTP)_2_]^−^ and [Cr­(^CF3^BTP)_2_]^−^ complexes are significantly more negative than those reported for tricationic Cr^III^ complexes such as [Cr­(dqp)_2_]^3+^ (−0.4 V) and [Cr­(ddpd)_2_]^3+^ (−0.7 V),^2,7^ suggesting that our complexes could potentially facilitate a wider range of reduction reactions after reductive ^2^E/^2^T_1_ excited state quenching by an electron donor.

**Figure 6:**
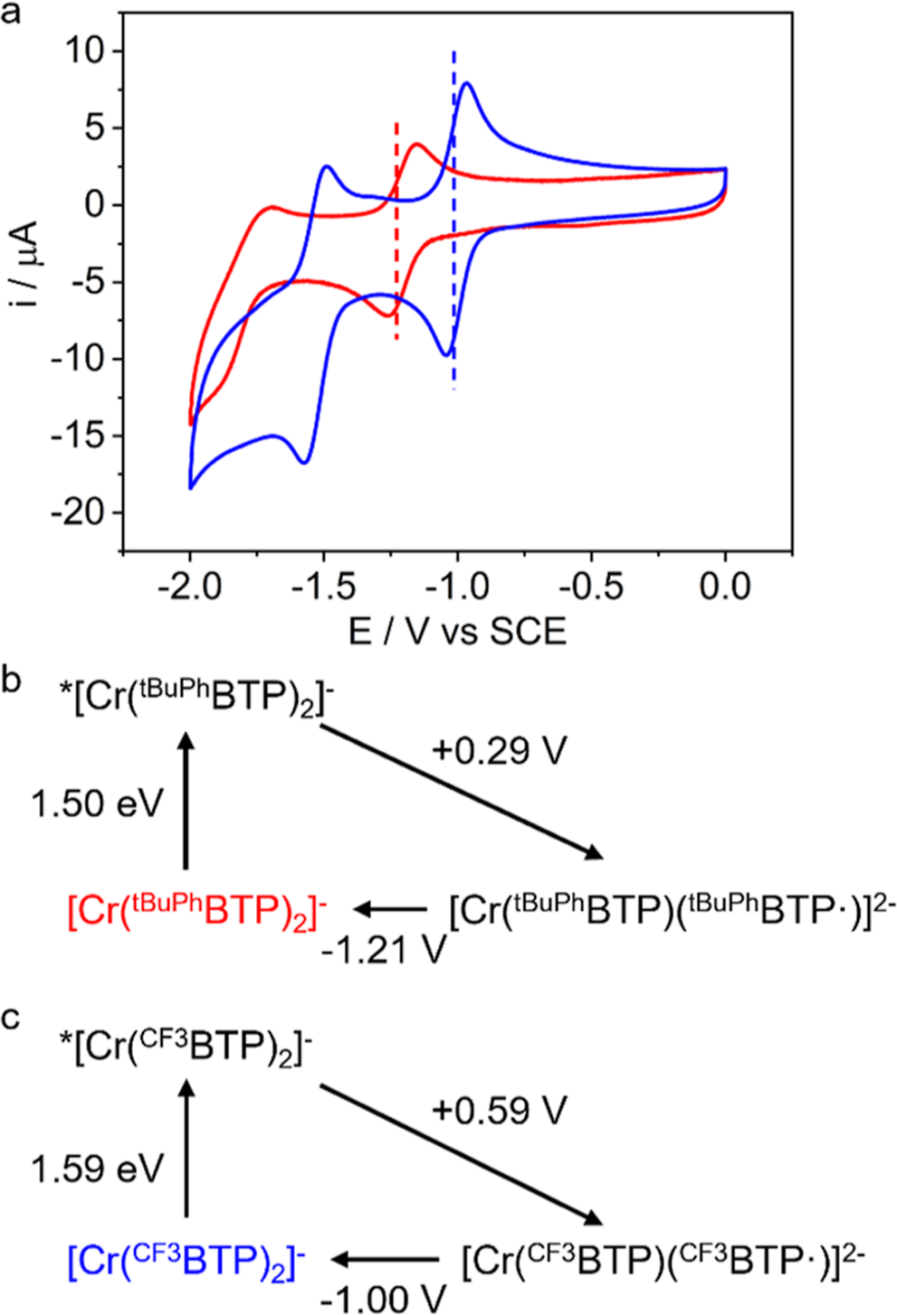
(a) Cyclic voltammograms of [Cr­(^*t*BuPh^BTP)_2_]^−^ (red) and [Cr­(^CF3^BTP)_2_]^−^ (blue), recorded from 0.5 mM solutions in Ar-flushed acetonitrile in the presence of 0.1 M *n*Bu_4_NPF_6_ as a supporting electrolyte. Scan rate: 0.2 V/s. Working electrode: glassy carbon. Counter electrode: silver wire. Reference electrode: KCl saturated calomel (SCE). (b,c) Latimer diagrams including the ground states and the photoactive ^2^E/^2^T_1_ excited states of [Cr­(^*t*BuPh^BTP)_2_]^−^ and [Cr­(^CF3^BTP)_2_]^−^.

Based on ^2^E/^2^T_1_ excited-state energies of 1.50 and 1.59 eV, we estimate excited-state reduction potentials of +0.29 V vs SCE for [Cr­(^*t*BuPh^BTP)_2_]^−^ and +0.59 V vs SCE for [Cr­(^CF3^BTP)_2_]^−^ (Figure 6b,c), which are significantly lower than those of the aforementioned tricationic Cr^III^ complexes. For comparison, [Cr­(dqp)_2_]^3+^ has a reduction potential of +1.26 V vs SCE^7^ in the ^2^E/^2^T_1_ excited state, whereas for [Cr­(ddpd)_2_]^3+^ the respective potential is +0.87 V vs SCE.^2^ Cr^III^ polypyridine photooxidants usually undergo reduction at the ligand while the Cr center relaxes back to a quartet state.^54,55^ In other words, the reduction is coupled to a reverse spin-flip relative to the initial excitation. This reverse spin-flip may contribute to the overall reorganization energy involved in the electron transfer process. In the case of our complexes, the metal–ligand bond exhibits significantly greater covalency compared with polypyridines, which makes it more challenging to distinguish between metal-centered and ligand-based reductions.

The excited-state potentials of our complexes fall below the threshold typically considered favorable for reductive quenching by aliphatic amine donors.^56^ However, the ^2^E/^2^T_1_ lifetimes of our complexes are significantly longer than the typical excited-state lifetimes of d^6^ metal-based complexes or organic photooxidants.^57–59^ Therefore, kinetic factors can outweigh thermodynamic ones, as demonstrated by the analysis in the following section.

### Exploring the Thermodynamic and Kinetic Limits of Reductive Excited-State Quenching

The [Cr­(^CF3^BTP)_2_]^−^ complex is a stronger photo-oxidant and better electron acceptor than [Cr­(^*t*BuPh^BTP)_2_]^−^, yet we chose to focus on [Cr­(^*t*BuPh^BTP)_2_]^−^ for reductive excited state quenching experiments with different electron donors because the one-electron reduced form of this complex will provide more driving force in reductive photocatalysis than [Cr­(^CF3^BTP)_2_]^−^ (Figure 1c,d). We performed ^2^E/^2^T_1_ excited state quenching experiments with 9 different electron donors (EDs) with oxidation potentials ranging from −0.61 to +0.81 V (Figure 7), covering a range of reaction free energies for photoinduced electron transfer (Δ*G*_ET_) with ^2^E/^2^T_1_-excited [Cr­(^*t*BuPh^BTP)_2_]^−^ between −0.90 and +0.52 eV. Either the transient absorption decay at 680 nm (Figure 4c) or the corrected emission intensity was used as an observable. Rate constants for bimolecular excited state quenching (*k*_q_) were determined for Stern–Volmer analyses and covered a range from 2.5 × 10^10^ to 4.8 × 10^3^ M^–1^ s^–1^ (Figure 7).

**Figure 7:**
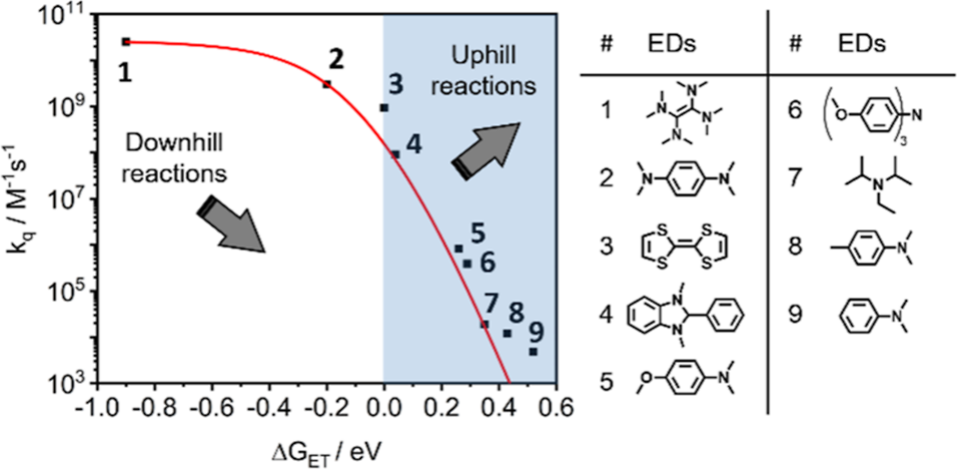
Rate constants for bimolecular electron transfer (*k*_q_) from selected electron donors (EDs, table on the right) to photoexcited [Cr­(^*t*BuPh^BTP)_2_]^−^ (black squares) as a function of the reaction free energy (Δ*G*_ET_). The red line describes the trend obtained by fitting the data with the empirical Rehm–Weller equation (Supporting Information). The best fit was obtained with a diffusion constant *k*_d_ of 3.0 × 10^10^ M^–1^ s^–1^, a parameter m accounting for the dissociation of the Cr^III^–ED encounter complex of 0.025, and a free activation energy Δ*G*_ET_^‡^ (0) of 0.23 eV in acetonitrile at the point where Δ*G*_ET_ = 0.

Typical Rehm–Weller behavior is observed, and the dependence of *k*_q_ on Δ*G*_ET_ can be described empirically by^60,61^

1 $$k_{q}=\frac{k_{d}}{1+m\left[\exp\left(\frac{\Delta G_{ET}^{‡}}{k_{B}T}\right)+\exp\left(\frac{\Delta G_{ET}}{k_{B}T}\right)\right]}$$

where *k*_d_ is the diffusion rate constant, *k*_B_ is the Boltzmann constant, and *T* is the absolute temperature (298 K). *m* is an empirical factor that accounts for the rate at which the encounter complex (formed between the excited state of the Cr^III^ complex and the electron donor) dissociates before electron transfer occurs. The activation energy, Δ*G*_ET_^‡^, is given by

2 $$\Delta G_{ET}^{‡}={\left[{\left(\frac{\Delta G_{ET}}{2}\right)}^{2}+\Delta G_{ET}^{‡}{\left(0\right)}^{2}\right]}^{\frac{1}{2}}+\left(\frac{\Delta G_{ET}}{2}\right)$$

Here, Δ*G*_ET_‡(0) represents the free energy of activation when Δ*G*_ET_ = 0. Our experimental data are best fit with *k*_d_ = 3 × 10^10^ M^–1^ s^–1^ and Δ*G*_ET_‡ = 0.23 eV using *m* = 0.025 (red trace in Figure 7). Based on Marcus theory for electron transfer, the Δ*G*_ET_‡ value of 0.23 eV translates into a reorganization energy of 0.92 eV (eq S14) for the photoinduced electron transfer reaction considered here. This is a typical value for reactions of this type.^62–64^

The most important finding of our ^2^E/^2^T_1_ excited-state quenching experiments and Rehm–Weller analysis is that [Cr­(^*t*BuPh^BTP)_2_]^−^ enables photoinduced electron transfer reactions that are endergonic up to 0.52 eV. In fact, most of the explored electron donors lead to uphill electron transfer (blue shaded area in Figure 7). This favorable behavior is largely due to the comparatively long ^2^E/^2^T_1_ excited state lifetime (23 μs, Figure 4c) of [Cr­(^*t*BuPh^BTP)_2_]^−^, which is more than an order of magnitude longer than for common transition metal-based photosensitizers.^26,65,66^ The behavior observed here is reminiscent of recent studies conducted in a more synthetically oriented context, where elementary reaction steps that were thermodynamically uphill by 0.3 eV were described as “unforeseen energy-transfer-based transformations”.^67,68^ Here, we observe electron transfer processes that are nearly twice as energetically uphill despite the fact that electron transfer processes are usually associated with larger activation barriers due to higher reorganization energies compared to energy transfer processes. Under the straightforward condition that the excited-state lifetime is sufficiently long to accommodate relatively slow electron transfer rates on the order of 10^4^ M^–1^ s^–1^, this behavior aligns with expectations based on the original Rehm–Weller studies and photochemistry textbooks.^61,69^ The endergonic region of photoinduced electron transfer reactions is usually less in focus, as most investigators aim to maximize the excited state quenching by achieving high *k*_q_ values. However, this focus neglects the importance of cage escape, i.e., the process in which the geminate radical pair formed immediately after the photoreaction ([Cr]^2–^/ED_1_^•+^ in Figure 8a) dissociates, leading to separately dissolved primary oxidation and reduction products. The efficiency of cage escape governs the overall photoreaction quantum yield^70^ and therefore represents an important reaction design factor that seems currently yet underappreciated. The cage escape quantum yield depends on many different factors,^71^ among which the reaction free energy for charge recombination (Δ*G*_iCR_) can be important.^70,72–74^

**Figure 8:**
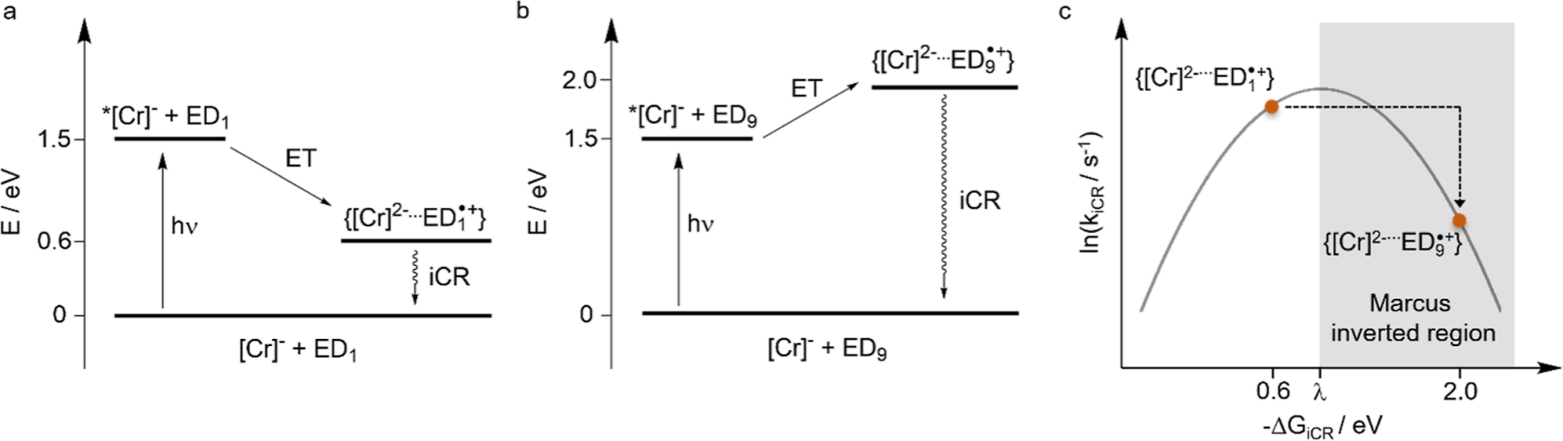
(a) Energy level diagram for the exergonic (Δ*G*_ET_ = −0.9 eV) photoinduced electron transfer (ET) from tetrakis­(dimethylamino)­ethylene (TDAE, ED_1_) to the excited state of the photocatalyst (*­[Cr]^−^) and following in-cage charge recombination (iCR) in the encounter complex; (b) energy level diagram for the endergonic (Δ*G*_ET_ = +0.5 eV) photoinduced electron transfer from *N*,*N*-dimethylaniline (DMA, ED_9_). The free energy of the charge recombination is still negative (−2.0 eV), but with a higher absolute value than the previous case (−0.6 eV); (c) classical Marcus parabola describing the driving-force dependence of the rate constant for in-cage charge recombination (*k*_iCR_). The unwanted back electron transfer from the reduced photocatalyst [Cr]^2–^ to ED_9^•+^_ occurs in the inverted regime further away from the maximum of the parabola, and therefore can be slower than the respective back electron transfer from [Cr]^2–^ to ED_1^•+^_. For simplicity, short symbols representing the reduced complex are shown, but the reduction is ligand-based, as displayed in the Latimer diagram in Figure 6b,c.

Fast downhill photoinduced electron transfer reactions are more likely to provide geminate radical pairs, for which Δ*G*_iCR_ falls into the normal or activationless regime of the Marcus theory for electron transfer than for slower uphill photoinduced electron transfer. For instance, electron donor 1 (Figure 7) reacts with *k*_q_ = 2.5 × 10^10^ M^–1^ s^–1^ and yields a geminate radical pair (abbreviated as [Cr]^2–^···ED_1_^•+^ in Figure 8a) that stores approximately 0.6 eV. Assuming that the reorganization energy associated with in-cage charge recombination is of similar magnitude as the reorganization energy for photoinduced charge separation (0.92 eV, see above), this implies rapid charge recombination near the top of the Marcus parabola. When instead considering electron donor 9 (Figure 7), the resulting geminate radical pair (abbreviated as [Cr]^2–^···ED_9_^•+^ in Figure 8b) stores 2.0 eV and as such likely falls into the Marcus inverted region (Figure 8c), for which slower in-cage charge recombination is expected.^70–74^ Consequently, we expect a trade-off between efficient initial photoinduced electron transfer and efficient in-cage charge recombination. In other words, the reactions providing the fastest excited state quenching are not necessarily the most productive ones in the overall picture.

Unfortunately, an in-depth analysis of this important interplay with our complexes is tricky due to several inherent challenges. First and foremost, many of the investigated electron donors (Figure 7) do not have characteristic UV–vis absorption bands in their one-electron oxidized forms, which makes their detection by transient absorption tricky and cage escape quantum yield measurements very difficult. Also, the reduced form of the photocatalyst (Figure S20) exhibits weak features in the sensitivity range of the detector, and its most prominent peaks overlap with the intense transient absorption signal of the doublet excited state (Figure 4c). The Cr^III^ ^2^E/^2^T_1_ ESA bands mask nearly the entire visible spectral range (Figure 4c), and unless photoreactions with very high *k*_q_ values are monitored, it is therefore very difficult to observe the primary [Cr]^2–^ reduction products, especially when cage escape quantum yields are modest. Nonetheless, the considerations summarized in Figure 8 seem valid and might represent an underappreciated aspect in current research on photoredox catalysis. The results presented in the next section offer indirect support for the validity of the arguments presented in Figure 8.

### Exploiting Uphill Reductive Excited-State Quenching for Thermodynamically Challenging Photocatalysis

Tertiary amines are widely used as electron donors in photoredox catalysis with noble metal-based or organic photocatalysts to generate a strongly reducing species after an initial photoinduced electron transfer step.^75,76^ With photoactive Cr^III^ complexes, this strategy is comparatively little explored because their one-electron reduced forms are usually very modest reducing agents. However, the molecular design of our [Cr­(^*t*BuPh^BTP)_2_]^−^ and [Cr­(^CF3^BTP)_2_]^−^ complexes (Figure 1) is such that one can expect reducing power comparable to those reachable with many Ru^II^ polypyridine complexes.^66,77,78^ Against this background and the results obtained from the previous section, we explored a benchmark photoreduction reaction and a singlet-oxygen-based photoreaction (Figure 9).

**Figure 9:**
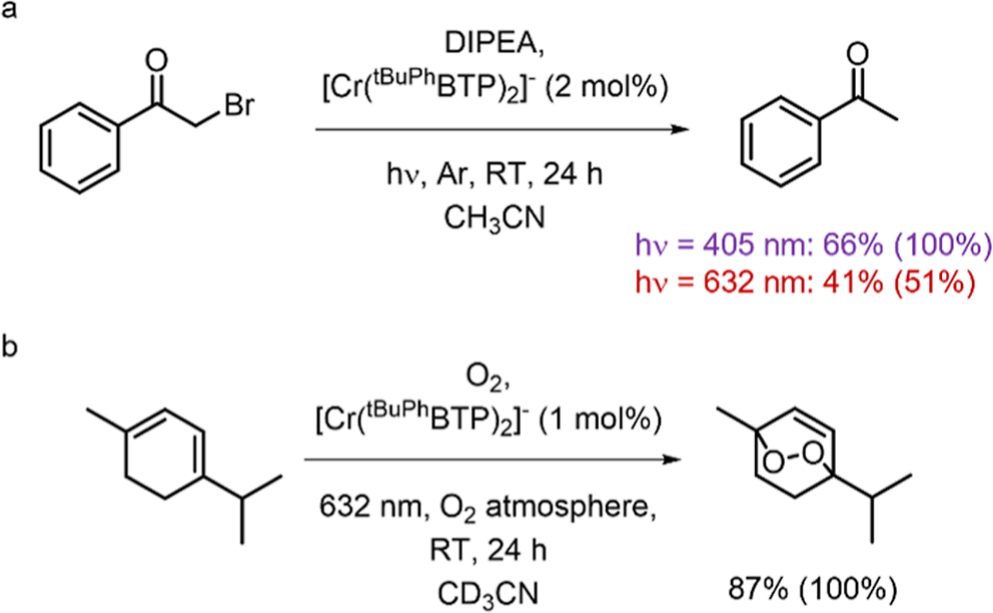
(a) Photocatalytic reductive debromination of α-bromoacetophenone upon irradiation with either a 405 or 632 nm LED and (b) oxidation of α-terpinene by singlet oxygen following irradiation at 632 nm in the presence of [Cr­(^*t*BuPh^BTP)_2_]^−^. The product yields (and the substrate conversions reported in parentheses) were determined by HPLC measurements (using suitable calibration curves) in the case of (a) or by ^1^H NMR spectroscopy referred to an internal standard for (b).

As a first proof-of-concept experiment, we chose the debromination of α-bromoacetophenone (*E*_red_ = −0.86 V,^79^ Figure 9a), which has been shown to efficiently occur upon reductive quenching of Ru^II^ complexes^70,80^ and inefficiently when using [Cr­(dqp)_2_]^3+^.^70^ Diisopropylethylamine (DIPEA, number 7 in Figure 7) was chosen as an electron donor (*E*_ED+/ED_ = +0.64 V vs SCE),^81^ implying endergonic ^2^E/^2^T_1_ excited-state quenching (Figure 7) and a scenario likely resembling that of ED_9_ in Figure 8b. We used a DIPEA concentration of 800 mM to enable fast hydrogen atom transfer in the final product-forming elementary reaction step and to ensure a 24% efficiency of the ^2^E/^2^T_1_ excited-state quenching.

Continuous irradiation with an LED at 405 nm, where the [Cr­(^*t*BuPh^BTP)_2_]^−^ complex has a molar extinction coefficient ε of 2800 M^–1^ cm^–1^, led to a 66% product yield after 24 h and an overall photoreaction quantum yield (Φ_R_, see Supporting Information) equal to roughly 1% (Table 2). Under these conditions, more than 99% of the incident 405 nm light is absorbed by the Cr^III^ complex. The obtained Φ_R_ value is 30 times higher than that obtained with the [Cr­(dqp)_2_]^3+^ photocatalyst in the presence of *N*,*N*-dimethyl-*p*-toluidine (DMT) as an electron donor.^70^ [Cr­(^*t*BuPh^BTP)_2_]^−^ is a weaker photooxidant and therefore undergoes less efficient ^2^E/^2^T_1_ excited state quenching than [Cr­(dqp)_2_]^3+^, which displays a quenching efficiency of essentially 100%, suggesting that a higher cage escape quantum yield for [Cr­(^*t*BuPh^BTP)_2_]^−^ is responsible for the greater overall photoreaction quantum yield (Φ_R_) obtained for the new complex. These findings are in line with the discussion in the previous section, according to which endergonic initial photoinduced electron transfer can provide better overall photoreactivity (Figure 8c). In the specific cases considered here, Δ*G*_ET_ = −0.54 eV and *k*_q_ = 9.8 × 10^9^ M^–1^ s^–1^ for the reaction between [Cr­(dqp)_2_]^3+^ and DMT, whereas for the [Cr­(^*t*BuPh^BTP)_2_]^−^/DIPEA couple Δ*G*_ET_ = +0.35 eV and *k*_q_ = 1.9 × 10^4^ M^–1^ s^–1^. The in-cage charge recombination between the photoreduced [Cr­(dqp)_2_]^2+^ and the oxidized electron donor, DMT^•+^, is less exergonic (Δ*G*_iCR_ = −1.1 eV) than that between [Cr­(^*t*BuPh^BTP)_2_]^2–^ and DIPEA^•+^ (Δ*G*_iCR_ = −1.8 eV). It is therefore plausible that in-cage charge recombination in the [Cr­(^*t*BuPh^BTP)_2_]^2–^/DIPEA^•+^ pair is less kinetically favored due to the Marcus inverted region effect (Figure 8c),^70,72–74^ thereby increasing the cage escape quantum yield and enhancing the overall reaction efficiency of [Cr­(^*t*BuPh^BTP)_2_]^−^ relative to [Cr­(dqp)_2_]^3+^.

**Table 2:** Comparison Between the Photoproduct Yields and the Overall Photoreaction Quantum Yields (Φ_R_) for the Photoreduction of α-Bromoacetophenone to Acetophenone (Figure 9a) With [Cr­(dqp)_2_]^3+^ and [Cr­(^*t*BuPh^BTP)_2_]^−^ as Photocatalysts

photocatalyst	λ_ex_/nm	time/h	photoproduct yield/%	Φ_R_/%
[Cr(dqp)_2_]^3+^ ^a^	415	6	<10	0.03
[Cr(^*t*BuPh^BTP)_2_]^−^	405	24	66	1
[Cr(^*t*BuPh^BTP)_2_]^−^	632	24	41	0.13

a 100 mM *N*,*N*-dimethyl-*p*-toluidine (DMT) was used as an electron donor (ED_8_) in this specific case. At this DMT concentration, the quenching efficiency is 1.^70^ λ_ex_ denotes the excitation wavelength.

The photoreaction in Figure 9a can also occur in the absence of a photocatalyst, as recently reported,^82^ leading in our case to a product yield of 42%. This reaction resulting from direct excitation of the reagent proceeds through a different mechanism and therefore does not affect our conceptual findings above. In the presence of 2 mol % of [Cr­(^*t*BuPh^BTP)_2_]^−^, more than 99% of the incident 405 nm LED light is absorbed by the Cr^III^ complex (Figure S41); hence, the reaction channel involving direct excitation of the reagent is unimportant under these conditions.

Photostability tests of [Cr­(^*t*BuPh^BTP)_2_]^−^ were performed upon continuous irradiation at 405 nm under air-equilibrated or inert conditions (see Supporting Information for further details). In both cases, the photodegradation quantum yield resulted in being around 2.5 × 10^–4^ %, indicating resistance to singlet oxygen and good stability under inert conditions.^7^ For comparison, Ru^II^ and Ir^III^ complexes have been reported to exhibit significantly higher photodegradation quantum yields under comparable conditions.^83^

### Improving the Light-to-Chemical Energy Conversion Efficiency by Using Red Excitation

Photons absorbed at 405 nm (3.06 eV) promote [Cr­(^*t*BuPh^BTP)_2_]^−^ to higher electronically excited states, which rapidly relax to the long-lived ^2^E/^2^T_1_ state with nearly unit efficiency.^43,84,85^ Since the ^2^E/^2^T_1_ excited state has an energy of only about 1.50 eV, the excess energy of 1.56 eV is lost through nonradiative channels and therefore cannot be exploited for photochemical reactions. In other words, when using 405 nm excitation, the maximum light-to-chemical energy conversion efficiency is below 50% (≤1.50 eV/3.06 eV). This is a common challenge with Cr^III^-based photocatalysts,^27^ because they absorb mostly in the blue but very weakly in the red spectral range due to the spin-forbidden nature of the ^4^A_2_ → ^2^E/^2^T_1_ transitions.^86^ This is particularly relevant for first-row transition metal complexes, which do not display elevated spin–orbit coupling.^26,87,88^ Although photocatalysis driven by red light is generally on the rise,^13,89–95^ little research has been carried out with photoactive Cr^III^ complexes in this regard.

At 632 nm (1.96 eV), the [Cr­(^*t*BuPh^BTP)_2_]^−^ complex absorbs with ε = 13 M^–1^ cm^–1^, roughly 200 times weaker than at 405 nm. Despite this, the debromination of α-bromoacetophenone proceeded successfully, achieving 51% substrate conversion and 41% product yield after 24 h of continuous irradiation at 632 nm. The control experiment (performed in the absence of Cr^III^ complex) resulted in 40% conversion and a much lower yield (19%). The quantum yield of the photoreaction in the presence of the [Cr­(^*t*BuPh^BTP)_2_]^−^ photocatalyst was 0.13%, still superior to the value obtained for [Cr­(dqp)_2_]^3+^ (Table 2),^70^ but lower than the one observed under 405 nm excitation (∼1%). The finding of an approximately 7-fold lower total quantum yield (Φ_R_) of the photoreaction after red excitation compared to blue excitation could have various causes and is difficult to narrow down here. One possibility is better cage escape when more energetic excitation is used, and this aspect probably deserves further attention with other systems that provide the necessary spectroscopic handles to measure this directly.

As a second red-light-driven photoreaction, we investigated the oxidation of α-terpinene to ascaridole (Figure 9b) mediated by singlet oxygen. In acetonitrile at room temperature, singlet oxygen quenches the ^2^E/^2^T_1_ excited state of [Cr­(^*t*BuPh^BTP)_2_]^−^ with a rate constant of 5.3 × 10^7^ M^–1^ s^–1^ and an efficiency of 67% (Supporting Information). The singlet oxygen quantum yield is 70% according to actinometry, closely matching the quenching efficiency and thus indicating that singlet oxygen formation is the main reaction channel. The ^1^O_2_ generation quantum yield determined for [Cr­(^*t*BuPh^BTP)_2_]^−^ is comparable to that of [Cr­(ddpd)_2_]^3+^, despite the steric hindrance introduced by the (*tert*-butyl)-phenyl substituents.^96^ This seems noteworthy because recent work found that sterically demanding coordination spheres can protect photoactive Cr^III^ from oxygen quenching.^38^ The crystal structure of [Cr­(^*t*BuPh^BTP)_2_]^−^ (Figure 3b) reveals sufficient space for oxygen to closely approach the metal center, enabling effective quenching of the spin-flip excited state of Cr^III^. Upon irradiation of [Cr­(^*t*BuPh^BTP)_2_]^−^ at 632 nm, α-terpinene was oxidized to ascaridole with a conversion of 25.9% and a yield of 20.5% in air-equilibrated solution and a quantitative conversion and a yield of 87% in an oxygen atmosphere after 24 h.

The light-to-chemical energy conversion efficiency achieved here with respect to ^1^O_2_ formation seems to be thought-provoking from a fundamental conceptual perspective: 632 nm (1.96 eV) photons can in principle be converted with 70% efficiency into a photoproduct (^1^O_2_) storing 0.96 eV, implying that even under the seemingly favorable conditions of red light excitation, only about 34% of the photon energy can ultimately be exploited for ^1^O_2_ formation. For comparison purposes, methylene blue, a widely used molecule for the generation of singlet oxygen,^97^ exhibits a quantum yield of 52% for Dexter energy transfer to O_2_ in acetonitrile.^98^ Upon excitation at the maximum absorption wavelength (664 nm or 1.91 eV), methylene blue converts only 26% of the photon energy.

### Doublet–Doublet Annihilation and Excited-State Disproportionation: Underappreciated Processes?

In the course of our basic photophysical experiments with [Cr­(^*t*BuPh^BTP)_2_]^−^, we found up to a 20% difference in the ^2^E/^2^T_1_ excited state lifetimes, depending on the exact measurement conditions. This prompted us to explore the dependency of the ^2^E/^2^T_1_ excited state lifetime as a function of the excitation energy (*P*_exc_) deposited by pulses of ca. 10 ns duration. In a first series of experiments, a 0.1 mM solution of [Cr­(^*t*BuPh^BTP)_2_]^−^ was excited at 355 nm, and ^2^E/^2^T_1_ lifetimes increased from 21 to 24 μs when decreasing *P*_exc_ from 60 to 15 mJ (blue circles in Figure 10a). In a second series of experiments, we used a 7.0 mM solution of [Cr­(^*t*BuPh^BTP)_2_]^−^ and excited the Cr^III^ complex at 532 nm by using the frequency-doubled (instead of the frequency-tripled) output of the same laser. This 7.0 mM solution has the same absorbance (0.4) at 532 nm as the 0.1 mM solution at 355 nm. In the more concentrated solution, the ^2^E/^2^T_1_ excited state lifetimes are lower by a factor of 2 at equal excitation pulse energy, and they further decrease when *P*_exc_ is increased (red circles in Figure 10a). For both solutions, we found an inversely proportional relationship between the ^2^E/^2^T_1_ lifetime and the square root of the excitation pulse energy (solid lines in Figure 10a). This inversely proportional relationship can be interpreted in terms of a deactivation process of the ^2^E/^2^T_1_ excited state that becomes more important with increasing excitation power (Supporting Information, Section 13), and the fact that the square root of *P*_exc_ (and not simply *P*_exc_) is relevant suggests a biphotonic process involving two ^2^E/^2^T_1_ excited Cr^III^ species. Higher initial Cr^III^ concentrations are likely helpful in reaching the regime where these biphotonic processes become important, hence the marked difference between the ^2^E/^2^T_1_ lifetimes determined from solutions with 0.1 mM and 7.0 mM concentrations.

**Figure 10:**
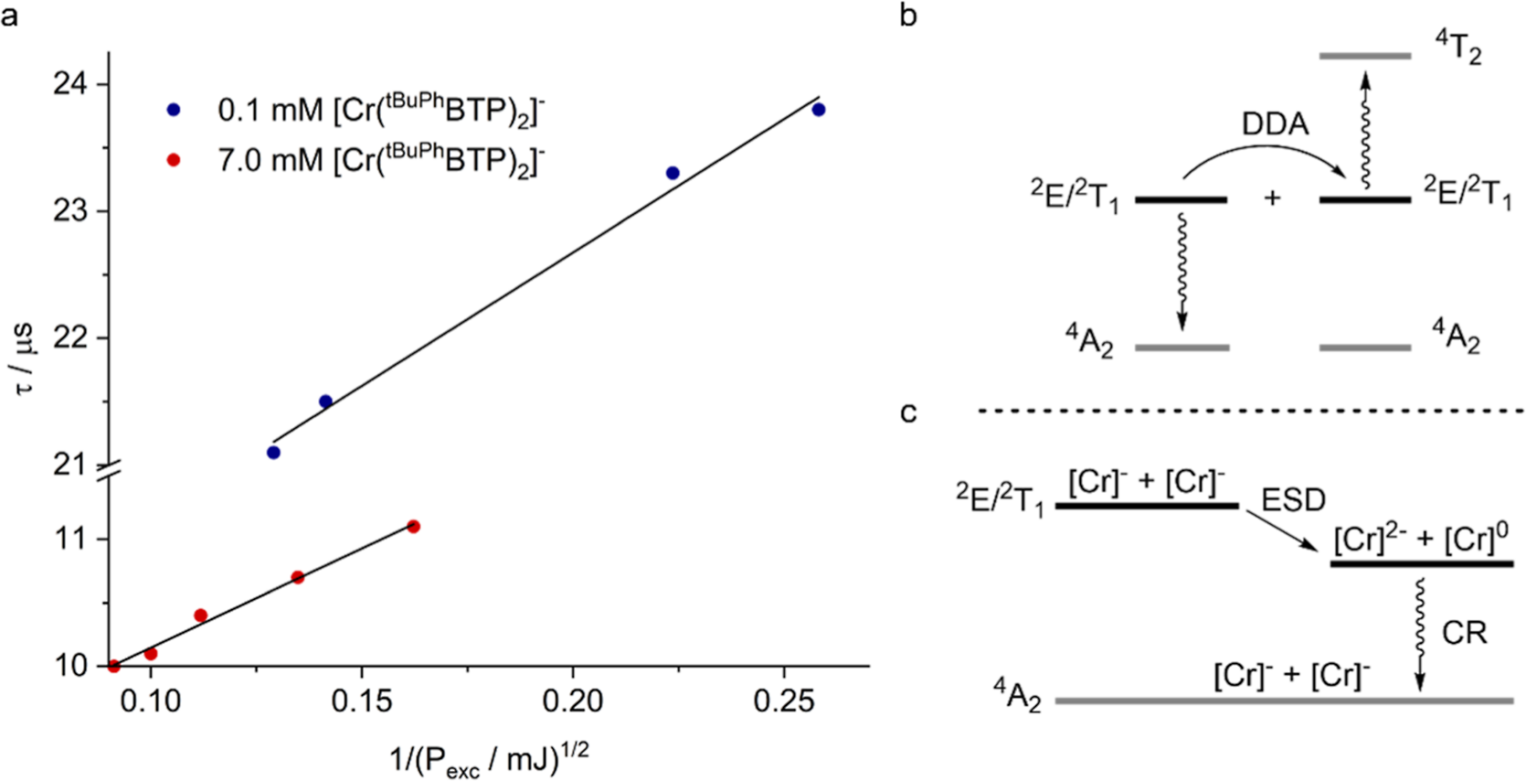
(a) Dependence of the lifetime of the ^2^E/^2^T_1_ excited state of [Cr­(^*t*BuPh^BTP)_2_]^−^ (τ) in Ar-flushed acetonitrile at 25 °C on the excitation pulse energy (P_exc_) in mJ, displayed here in the form of its reciprocal square root. The duration of the excitation pulses was approximately 10 ns. The red circles were obtained from a 7.0 mM solution that was excited at 532 nm, whereas the blue circles were recorded from a 0.1 mM solution that was excited at 355 nm. Both solutions had equal absorbance (0.4) at the respective excitation wavelength. The ^2^E/^2^T_1_ lifetime was detected by monitoring the disappearance of the excited-state absorption at 680 nm. The solid black lines are linear regression fits to the experimental data. (b,c) Possible bimolecular processes affecting the ^2^E/^2^T_1_ lifetime: (b) doublet–doublet annihilation (DDA) and (c) excited-state disproportionation (ESD) followed by charge recombination (CR) to the quartet ground state. For simplicity and for consistency with Figure 8, short symbols representing the reduced and oxidized complex are shown in (c), as ESD could in principle involve both metal-centered and ligand-based redox events; see also Figure 6b,c.

The observations made for [Cr­(^*t*BuPh^BTP)_2_]^−^ in Figure 10 point toward a second-order reaction reminiscent of triplet–triplet annihilation.^99–103^ Doublet–-doublet annihilation (DDA) has in fact been reported for other Cr^III^ complexes more than 40 years ago (Figure 10b),^104,105^ but has remained largely unexplored since then. As noted earlier, in addition to doublet–doublet annihilation, there could also be excited-state disproportionation (ESD, Figure 10c), where two ^2^E/^2^T_1_-excited Cr^III^ complexes undergo electron transfer to form a one-electron oxidized and a one-electron reduced complex. A third possibility would be aggregation-induced quenching processes, though such behavior is more typical for complexes with other coordination numbers and geometries and for more planar molecular structures,^106–108^ or for ion pairs.^109,110^ Based on the reduction potential of −1.21 V vs SCE for [Cr­(^*t*BuPh^BTP)_2_]^−^ in the electronic ground state (Figure 6b), the energy stored in its ^2^E/^2^T_1_ excited state (1.50 eV) and assuming a (ground state) oxidation potential at +1.3 V vs SCE (Figure S19), the reaction free energy for the ESD process is −0.5 eV.

This analysis leaves both ESD and DDA as viable pathways for the behavior observed in Figure 10a, and at present, it is unclear which one dominates. However, it seems clear that the second-order excited-state quenching process observed by us here can potentially affect photocatalysis when high concentrations and excitation power densities are used. Furthermore, it is conceivable that in favorable cases the higher excited states generated by DDA can be exploited photochemically, although this would likely require further aggregation with substrate molecules to enable sufficiently fast reactions.^111,112,200^ Ultimately, we cannot exclude the possibility that these processes influence the mechanism of the photoreductions we reported, although this remains a speculative consideration.

## Conclusions

The majority of recent work on photoactive Cr^III^ complexes has focused on polypyridine coordination environments and pursued a molecular design strategy aimed at obtaining strong ligand fields to optimize luminescence quantum yields and excited state lifetimes.^40,42,45,87,201–114^ Our research consolidates a complementary molecular design strategy aimed at optimizing the covalency of the metal–ligand bond and creating more electron-rich Cr^III^ complexes that emit in the near-infrared rather than the red spectral region and are suitable for photochemical reductions in addition to photooxidation reactions. In terms of metal coordination properties, our triazole-based ligands more closely resemble previously studied carbazole,^6,46^ isoindole,^8,9^ pyrrole,^25^ and triazole^43^ ligand systems for Cr^III^ than traditional polypyridines.^115,116^

The photoactive spin-flip excited states of our new Cr^III^ complexes have lifetimes on the order of 5–30 μs, which permits reductive excited state quenching with weak electron donors in elementary reaction steps that are endergonic up to 0.5 eV (around 50 kJ/mol). In principle, this opens the door to red (or even near-infrared) light-driven photocatalysis of endergonic reactions relevant to solar energy conversion and artificial photosynthesis,^117,118^ and simple proof-of-concept reactions have been successfully performed.

In a wider context, our study addresses a perhaps underappreciated aspect of photoredox catalysis, namely, the extent to which fast excited-state quenching caused by strongly exergonic initial photochemical reaction steps is indeed desirable to optimize the overall reaction quantum yield. Fast exergonic initial photoinduced electron transfer can go hand in hand with fast reverse (energy-wasting) electron transfer elementary steps, resulting in low cage escape quantum yields, whereas slower endergonic photoinduced electron transfer offers the possibility of reaching the Marcus inverted regime for unwanted charge recombination, thereby slowing unwanted energy dissipation and potentially improving overall photoreaction quantum yields.^70^ It follows that there must be an optimal thermodynamic constellation between photoinduced forward and thermal reverse electron transfer that optimizes the ratio between energy-storing and energy-wasting reaction rates (Figure 8), similar to traditional work in donor-sensitizer-acceptor compounds aimed at mimicking the primary events of natural photosynthesis.^119,120^ In our case, we achieved a higher overall photoreaction quantum yield based on an initial photoinduced electron transfer step that is endergonic by 0.35 eV than for the same reaction triggered by −0.54 eV exergonic initial photoinduced electron transfer between a different donor/acceptor pair. We anticipate that the intricate interplay between excited state quenching and optimized cage escape quantum yields can be more broadly exploited to improve the reaction quantum yield in photocatalysis.^70^

Our finding of concentration- and excitation-power-dependent ^2^E/^2^T_1_ excited state lifetimes points to doublet–doublet annihilation as a relevant process triggered by photoactive Cr^III^ complexes, which might deserve more attention in future work. Doublet-triplet energy transfer has recently been successfully exploited for upconversion processes,^31,121–123^ and it seems conceivable that photoreactions involving two doublet excited states could also provide an entry point to conceptually new and useful photochemistry.

Overall, our study illustrates the critical interplay between the molecular design of photoactive transition complexes and the advancement of a fundamental understanding of elementary photochemical reaction steps.

## Supplementary Material



## Notes

Additional experimental details, materials, and methods, including equipment details, synthesis and characterization of ligand precursors and complexes, single crystal X-ray data, NMR and HR-ESI mass spectra, photophysical and electrochemical characterization, singlet oxygen generation quantum yield, Stern–Volmer experiment, photocatalytic experiments, and mathematical derivation of the lifetime dependency on the excitation pulse energy (PDF)
