# Supplementary material for: Pushing the Thermodynamic and Kinetic Limits of Near-Infrared Emissive CrIII Complexes in Photocatalysis
Source: J Am Chem Soc. 2025 Jul 28;147(31):28226–40. doi: 10.1021/jacs.5c08541 (PMC12333365; doi:10.1021/jacs.5c08541)
Supplement: Supplementary file 1 [file ja5c08541_si_001.pdf]

Supporting Information

Pushing the Thermodynamic and Kinetic Limits of Near-Infrared Emissive Cr^{III} Complexes in Photocatalysis

*Giacomo Morselli,[a] Tim H. Eggenweiler,[a] Marco Villa,[b] Alessandro Prescimone,[c] Oliver S. Wenger[a,]**

[a] Department of Chemistry, University of Basel, St. Johanns-Ring 19, 4056 Basel, Switzerland

[b] Department of Chemistry "G. Ciamician", University of Bologna, Via Selmi 2, 40126 Bologna, Italy

[c] Department of Chemistry, University of Basel, BPR 1096, Mattenstrasse 24a, 4058 Basel, Switzerland

Contents

1 Materials and methods S2
2 General synthesis procedures S4
2.1 Synthesis of ^{tBuPh}BTP S4
2.2 Synthesis of ^{CF3}BTP S5
2.3 Synthesis of (nBu$_4$N)[Cr(^{tBuPh}BTP)$_2$] and (PPN)[Cr(^{tBuPh}BTP)$_2$] S6
2.4 Synthesis of (nBu$_4$N)[Cr(^{CF3}BTP)$_2$] S7
2.5 NMR spectra S8
2.6 HR-ESI mass spectra S12
2.7 Crystal structures of (nBu$_4$N)[Cr(^{CF3}BTP)$_2$], (PPN)[Cr(^{tBuPh}BTP)$_2$] and octahedral distortion parameters S14
3 DFT calculations S17
4 Determination of 10 Dq, B and β S25
5 Further photophysical characterization S26
6 Electrochemical data S28
6.1 Electrochemistry of (nBu$_4$N)[Cr(^{tBuPh}BTP)$_2$] S28
6.2 Electrochemistry of (nBu$_4$N)[Cr(^{CF3}BTP)$_2$] S29
7 Emission quantum yield of (nBu$_4$N)[Cr(^{tBuPh}BTP)$_2$] S31
8 Singlet oxygen generation quantum yield and excited state quenching efficiency S32
9 Stern-Volmer experiments and Rehm-Weller analysis S34
10 HPLC-MS calibration curve of α-bromoacetophenone and acetophenone S39
11 Photocatalytic experiments S41
11.1 Dehalogenation of α-bromoacetophenone S41
11.2 Oxidation of α-terpinene with singlet oxygen S51
12 Photostability S57
12.1 Photostability in air-equilibrated solution S57
12.2 Photostability under inert (Ar) atmosphere S57
13 $^2E/^2T_1$ excited-state lifetime dependence on the Cr^{III} concentration and the excitation power S58
14 References S65

1 Materials and methods

If not stated otherwise, all reactions and manipulations were carried out under an atmosphere of dry nitrogen using Schlenk techniques or in a MBraun Glovebox under an argon atmosphere. The reagents were purchased from Acros, Fluorochem and ThermoScientific and used as received unless stated otherwise. $Cr(TfO)_2$ was obtained from Cr powder and triflic acid according to a previously reported procedure.[1]

NMR spectra were recorded at 298 K on AVANCE III 400 MHz and Bruker AVANCE III 250 MHz spectrometers. Chemical shift values are reported in δ values relative to TMS and were referenced to (residual) solvent signals (for ^{1}H-NMR, $CDCl_3$: δ = 7.26 ppm, CD_3CN: δ = 1.94 ppm; DMSO-d6: δ = 7.26 ppm). Abbreviations: s = singlet, d = doublet, m = multiplet, b = broad.

Analytical HPLC-MS was carried out on a Shimadzu LCMS-2020 single quadrupole instrument equipped with a diode array detector SPD-M30A. Compounds were separated using an Agilent ZORBAX XDB-C18 column (3.5 µm, 4.6 x 75 mm), using a gradient from solvent A (H_2O, 0.1% formic acid) to solvent B (acetonitrile, 0.1% formic acid). Injections of 10 µL of the solutions were made in 99% A, held at 99% A for 3 minutes, ramped to 99% B over 6 minutes. 99% B was held for 0.5 min, and then returned to starting conditions over 0.5 min and allowed to re-equilibrate for 2 minutes with a 3 mL/min constant flow rate.

Elemental analysis was performed by Sylvie Mittelheisser, with a Vario Micro Cube instrument from Elementar.

HRMS analyses were conducted by Dr. Michael Pfeffer on a Bruker maxis 4G ESI-Q-TOF under direct injection conditions with CH_3CN as solvent.

The synthetic reactions were monitored by thin layer chromatography using TLC Silica gel 60 plates coated with fluorescence indicator F254 (Merck). The compounds were visualized by UV absorption (254 or 365 nm). Purification by standard silica gel column chromatography was performed using SiliaFlash® P60, Silicycle. (Pore size: 40-63 µm).

For cyclic voltammetry, an SCE reference electrode, a glassy carbon disk as working electrode, and a silver wire as counter electrode were used in a glass cell containing the sample solution (0.5 mM Cr^{III} complex and 100 mM tetra-*n*-butylammonium hexafluorophosphate) in dry CH_3CN that was purged with argon for 5 min. A Versastat3-200 potentiostat from Princeton Applied Research was used to apply the voltage with a potential sweep rate of 200 mV/s. The same setup was used for spectro-electrochemical absorption measurements in quartz cuvettes with 1 mm path length in combination with an OceanViewHDX miniature spectrophotometer from Ocean Optics. A platinum grid as working electrode, a platinum wire as counter electrode, and an SCE reference electrode were employed. An integration time of 50 ms was used.

For photocatalytic studies, either a M405L3-C1 405 nm LED from Thorlabs® with a typical collimated output power of 250 mW, or a SOLIS-623C 632-nm LED from Thorlabs® with an output power of 2 W at a distance of 5 cm were employed. Unwanted heating of the reaction mixture by the LED radiation was minimized by placing the sample in a water bath.

All optical spectroscopic measurements were performed at 298 K in quartz cuvettes with septum caps with 1 cm path length in dry and deaerated solutions. The experiments were carried out in deoxygenated (5 freeze-pump-thaw cycles or Argon-flushed) acetonitrile. Optical absorption spectroscopy was performed using a Cary 5000 instrument from Varian. Steady-state luminescence spectra were measured on a Fluorolog-322 from Horiba Jobin-Yvon, equipped with a Xenon lamp 450-Watt Illuminator (FL-1039A/40A) and a water-cooled photomultiplier tube (PMT Hamamatsu R2658 or R928) or an Edinburgh FS5 with a PMT980 and an InGaAs detector for visible and NIR spectral ranges, respectively. Phosphorescence spectra (for singlet oxygen emission) were collected with an Edinburgh FLS920 spectrofluorometer equipped with a liquid-nitrogen-cooled Ge-detector.

For time resolved UV-Vis absorption measurements and excited state lifetime measurements, an LP920-KS apparatus from Edinburgh Instruments was used. A frequency-tripled and a frequency-doubled pulsed Nd:YAG laser (Quantel Q-smart 450, ca. 10 ns pulse width) with a beam expander (BE02-355 from Thorlabs) in the beam path were used for excitation at 355 nm and 532 nm (pulse energy of either ~35 mJ unless described otherwise), or a frequency-tripled Nd:YAG laser (Quantel Brilliant, ca. 10 ns pulse width) equipped with an OPO from Opotek and a beam expander (GBE02-A from Thorlabs) in the beam path was used for excitation at 447 nm (pulse energy of ~10 mJ). An iCCD camera from Andor was used to detect transient absorption spectra, and single-wavelength kinetics were recorded with a photomultiplier tube.

Time-resolved photoluminescence measurements were recorded with an FLS1000 instrument from Edinburgh.

The estimated experimental errors are: 2 nm on the band maxima, 10% on the molar absorption coefficient and photoluminescence quantum yield and 5% on the excited state lifetime.

Photostability studies were performed with a continuous-wave laser with excitation at 405 nm (RLTMDL-405-500-3 Roithner LaserTechnik, power at the irradiation distance: 0.3 W). Absorption spectra were collected at fixed times with an OceanViewHDX miniature spectrophotometer from Ocean Optics. The cuvette was maintained at 295 K by a thermostat system.

2 General synthesis procedures

2.1 Synthesis of ^{tBuPh}BTP

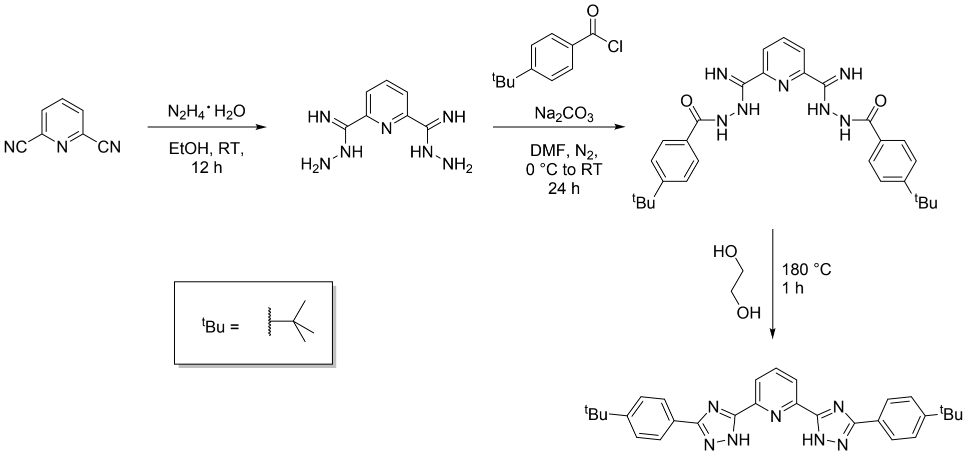

Scheme S1. General reaction procedure for the synthesis of ^{tBuPh}BTP involving acyl chlorides.

Method 1: Via acyl chlorides.

The procedure for the synthesis of ^{tBuPh}BTP (2,6-bis(3-(4-(*tert*-butyl)phenyl)-1H-1,2,4-triazol-5-yl)pyridine) was adapted from refs [2,3] (Scheme S1). Briefly, 420 mg of pyridine-2,6-dicarbonitrile (3.3 mmol) were suspended in 20 mL of absolute ethanol. 2 mL of hydrazine monohydrate ($N_2H_4{\cdot}H_2O$ 64%, 34 mmol) were added dropwise, leading first to dissolution of the starting material followed by the precipitation of the bis(carboximidhydrazide) as an off-white solid. The suspension was stirred overnight at room temperature before adding 20 mL of ethanol. Then it was sonicated, filtrated and washed with cold ethanol and *n*-pentane. The solid was transferred in a two-necked flask with 660 mg of anhydrous Na_2CO_3 (6.2 mmol), evacuated and heated at 50 °C for 10 minutes. Then, 5 mL of anhydrous DMF were added. In a separate flame-dried Schlenk flask, a solution of 4-(*tert*-butyl)benzoyl chloride (1.3 mL, 6.2 mmol) in 3 mL of anhydrous DMF was prepared under inert atmosphere. The acyl chloride solution was then transferred dropwise to the reaction flask at 0 °C under strong stirring and let slowly heat up to RT. The color of the solution turned yellow, followed by the precipitation of a yellow solid overnight. 20 mL of distilled water were added and the suspension was stirred for 1 h. The solid was filtrated on a frit and washed thoroughly with water. The solid was immediately used without any further purification. It was dispersed in 10 mL of ethylene glycol and heated up at 180 °C to dissolve the solid. After 1 h, the solution was cooled down to room temperature and water was added to yield a white solid precipitate. The solid was filtrated, washed with water to remove the excess of ethylene glycol and the product was purified by flash chromatography (6:4=cyclohexane:ethyl acetate, $R_f = 0.2$, blue fluorescent spot upon irradiation at 365 nm) to yield a white powder (30 mg, 0.63 mmol, 2% yield). The low yield is attributed to the competitive generation of formylation products, likely due to the formation of Vilsmeier reagent in DMF in the presence of acyl chlorides. A more efficient method was therefore developed (Scheme S2).

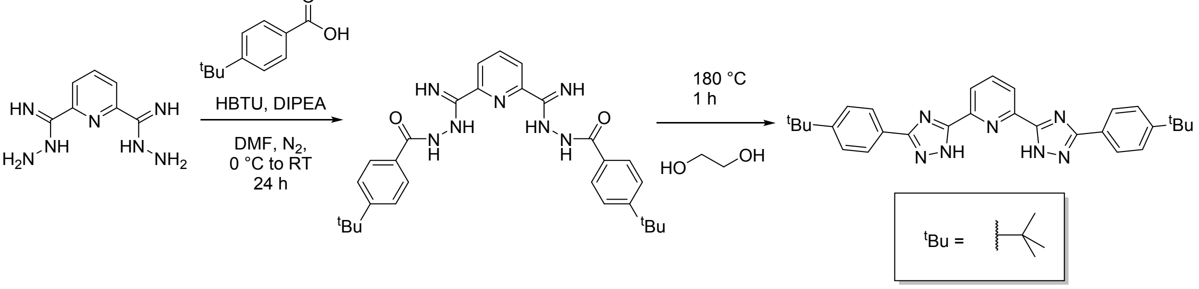

Scheme S2. General reaction procedure for the synthesis of ^{tBuPh}BTP involving carboxylic acids.

Method 1: Via carboxylic acids.

In a flame-dried two-necked 50-mL flask, 4-(*tert*-butyl)benzoic acid (250 mg, 1.4 mmol) was dissolved in anhydrous DMF (5 mL) together with 0.35 mL of DIPEA (2 mmol). At 0 °C, HBTU 2-(1H-benzotriazol-1-yl)-1,1,3,3-tetramethyluronium hexafluorophosphate, 531 mg, 1.4 mmol) was added stepwise and the reaction mixture was stirred for 10 minutes in an ice-cold water bath.

130 mg of bis(carboximidhydrazide) (0.67 mmol) obtained from the reaction between 200 mg of pyridine-2,6-dicarbonitrile (1.55 mmol) and 1 mL of hydrazine monohydrate ($N_2H_4{\cdot}H_2O$ 64%, 17 mmol) as described before, were suspended in 5 mL DMF. The white dispersion was added dropwise to the 4-(*tert*-butyl)benzoic acid mixture at 0 °C. After a first dissolution, a white solid started to precipitate. The mixture was let stir overnight at room temperature. 10 mL of water were then added, producing more precipitate. The suspension was stirred under air for 1 h and then filtrated over a frit. The solid was collected, suspended in 5 mL ethylene glycol and heated up at 180 °C to dissolve the solid. After 1 h, the solution was cooled to room temperature and water was added to obtain a white solid precipitate. The solid was filtrated, washed with water to remove the excess of ethylene glycol and dried at the vacuum line, yielding the product as a white solid (190 mg, 0.4 mmol, 60% yield).

^{1}H-NMR (DMSO-d$_6$, 400 MHz), δ(ppm) = 14.61 (bs, 2H), 8.21 (s, 3H), 8.07 (d, J = 8.2 Hz, 4H), 7.56 (d, J = 8.2 Hz, 4H), 1.34 (s, 18H). The data are in accordance with the ones reported in literature.[3]

ESI-HRMS (m/z): calculated for $[C_{29}H_{31}N_7+H]^+$: 478.2714; observed: 478.2706.

2.2 Synthesis of ^{CF3}BTP

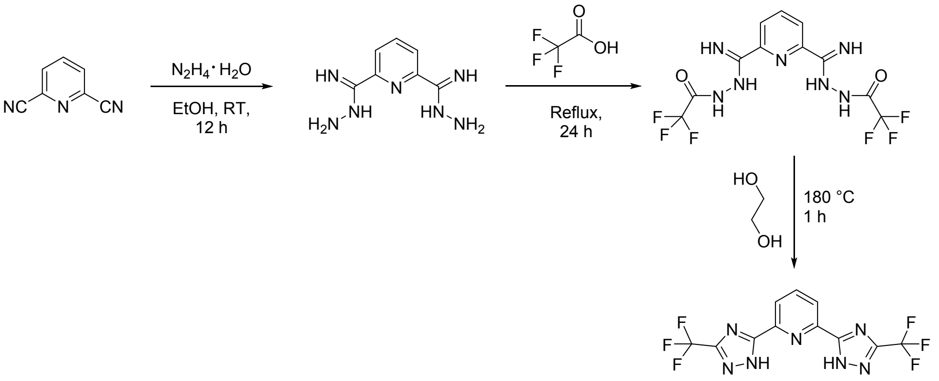

Scheme S3. General reaction procedure for the synthesis of ^{CF3}BTP

The procedure for the synthesis of ^{CF3}BTP (2,6-bis(3-(trifluoromethyl)-1H-1,2,4-triazol-5-yl)pyridine) was reproduced from ref[4] (Scheme S3). Briefly, 420 mg of pyridine-2,6-dicarbonitrile (3.3 mmol) were suspended in 20 mL of absolute ethanol. 2 mL of hydrazine monohydrate ($N_2H_4{\cdot}H_2O$ 64%, 41 mmol) were added dropwise, producing at first a yellow solution followed by the precipitation of an off-white solid. The suspension was stirred overnight at room temperature before adding 20 mL of ethanol. Then it was sonicated, filtrated and washed with cold ethanol and *n*-pentane. The solid was dissolved in 4 mL of trifluoroacetic acid. The color of the solution turned orange. The solution was refluxed for 2 days, concentrated under reduced pressure and dried at the vacuum line. The solid was immediately used without any further purification. It was dispersed in 10 mL of ethylene glycol and heated up at 180 °C to dissolve the solid. After 1 h, the solution was cooled to room temperature and water was added to obtain a white solid precipitate. The solid was washed with water to remove the excess of ethylene glycol and dried at the vacuum line, yielding an off-white solid (400 mg, 1.15 mmol, 37% yield).

^{1}H-NMR (MeCN-d$_3$, 400 MHz), δ(ppm) = 13.20 (s, 2H), 8.27 – 8.16 (m, 3H).

^{19}F{^{1}H}-NMR (DMSO-d6, 400 MHz), δ(ppm) = -65.94.

ESI-LC-MS (m/z) = 349 [M+H$^+$]. The data are consistent with the ones reported in literature.[4]

2.3 Synthesis of (nBu$_4$N)[Cr(^{tBuPh}BTP)$_2$] and (PPN)[Cr(^{tBuPh}BTP)$_2$]

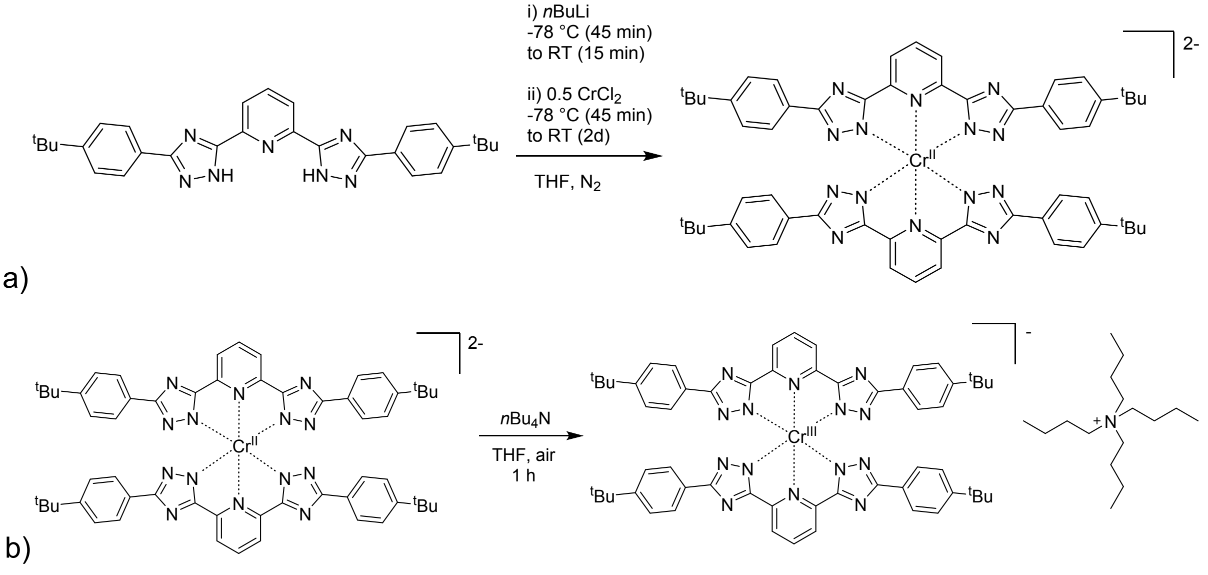

Scheme S4. Synthesis of (nBu$_4$N)[Cr(^{tBuPh}BTP)$_2$]. (a) Deprotonation of ^{tBuPh}BTP followed by complexation of Cr^{2+} and (b) oxidation of Cr^{II} to Cr^{III} under air and cation exchange.

92 mg of ^{tBuPh}BTP (0.2 mmol) were dissolved in 8 mL of anhydrous THF and degassed by flushing nitrogen or argon for 10 minutes. At -78 °C, in an acetone-dry ice bath, 170 µL of *n*-BuLi (2.5 M in hexanes, 0.4 mmol) were added dropwise. The solution color changed from yellow to red. The solution was let stir for 45 minutes at -78 °C, then the cold-acetone bath was removed and the reaction mixture was allowed to warm up for 15 minutes. During this time, the color of the suspension turned orange. After cooling back to -78 °C, a suspension of $CrCl_2$ (13 mg, 0.11 mmol) in 4 mL of dry deoxygenated THF was added dropwise. The color of the suspension turned dark green. The mixture was allowed to slowly heat up to room temperature and stirred for 2 days. The solution was opened to air, which caused a change of color to brown, and 40 mg of nBu$_4$NCl (tetra-*n*-butyl ammonium chloride, 0.14 mmol) were added. The solution was let stir for another hour, then the precipitate was filtered and the supernatant concentrated under reduced pressure. The obtained oil was dissolved in DCM and a solid was precipitated upon layering of *n*-pentane.

Column chromatography (gradient elution from 9:1 DCM/acetone to 9:1 acetone/MeOH) was used to purify the sample, which was again dissolved in acetone and precipitated with *n*-hexane. A brown powder was obtained (100 mg, 0.08 mg, 80% yield).

^{1}H-NMR (DMSO-d$_6$, 400 MHz), δ(ppm) = 8.12 (bs), 7.84 (bs), 7.37 (bs), 3.60 (bs), 3.16 (bs), 1.76 (bs), 1.56-1.44 (bm), 1.32 (s), 0.93 (bs).

ESI-HRMS (m/z): calculated for $[C_{58}H_{58}CrN_{14}]^-$: 1002.4379; observed: 1002.4400.

For (PPN)[Cr(^{tBuPh}BTP)$_2$], the same procedure was followed, but Cr(TfO)$_2$ and PPNCl (bis (triphenylphosphine) iminium chloride) were used instead of $CrCl_2$ and nBu$_4$NCl, respectively. Layering of *n*-hexane onto a concentrated solution of (PPN)[Cr(^{tBuPh}BTP)$_2$] in DCM yielded high quality single crystals for XRD and elemental analyses.

Elemental analysis, found: C, 72.22%; H, 6.00%; N, 13.12%. Calculated for $C_{94}H_{88}CrN_{15}P_2 \cdot 1.5H_2O$: C, 71.97%; H, 5.85%; N, 13.39%.

2.4 Synthesis of (nBu$_4$N)[Cr(^{CF3}BTP)$_2$]

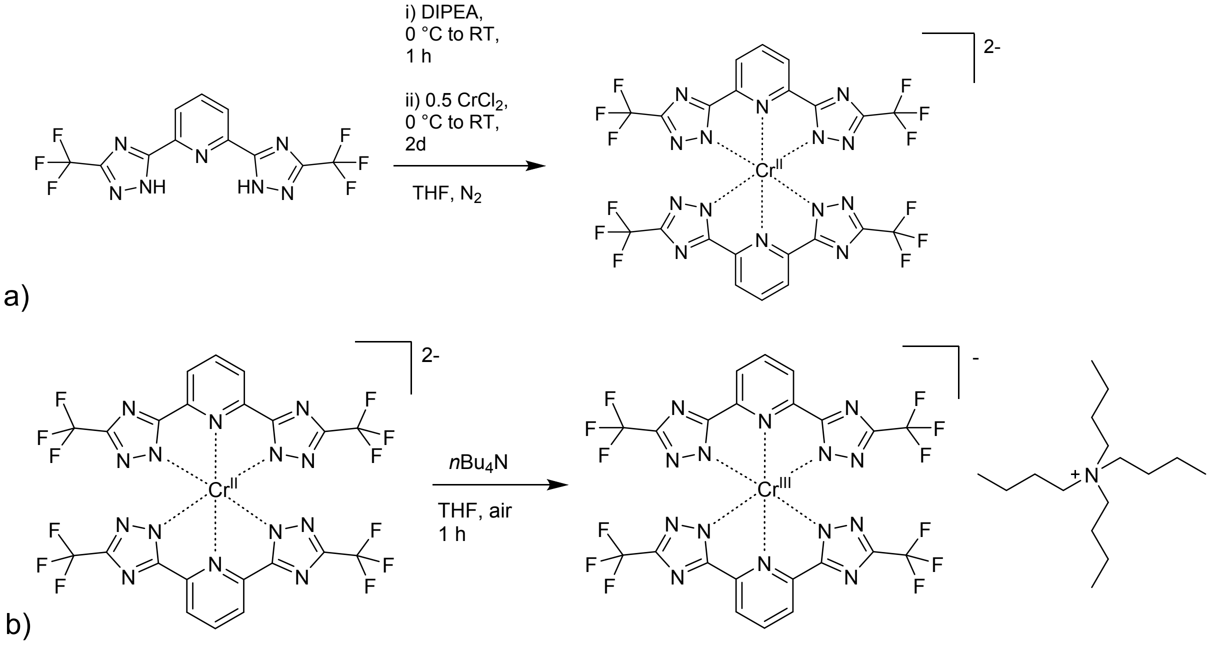

Scheme S5. Synthesis of (nBu$_4$N)[Cr(^{CF3}BTP)$_2$]. (a) Exposure of ^{CF3}BTP to DIPEA followed by complexation of Cr^{2+} and (b) oxidation of Cr^{II} to Cr^{III} under air and cation exchange.

70 mg of ^{CF3}BTP (0.2 mmol) were dissolved in 8 mL of anhydrous THF and degassed by flushing with nitrogen or argon for 10 minutes. At 0 °C, DIPEA (75 µL, 0.42 mmol) was added dropwise, and the mixture was stirred at room temperature for 1 h. At 0 °C, a suspension of $CrCl_2$ (15 mg, 0.12 mmol) in 4 mL of dry deoxygenated THF was added dropwise. The color changed to dark green. The solution was allowed to heat to room temperature and stirred for 2 days. The flask was the opened to air and 33 mg of nBu$_4$NCl (0.12 mmol) were added. After 1 h, the precipitate was filtered and the supernatant concentrated at reduced pressure. Flash chromatography (1:9 acetone/DCM to 1:1 acetone/DCM) was performed to purify the complex. Slow diffusion of Et_2O vapors into a concentrated solution in DCM yielded high-purity orange crystals suitable for XRD and elemental analyses (190 mg, 0.19 mmol, 96% yield).

High-quality ^{1}H-NMR (DMSO-d$_{6,}$ 400 MHz) couldn't be obtained due to the presence of paramagnetic nature of the Cr^{III} complex.

^{19}F{^{1}H}-NMR (DMSO-d$_6$, 400 MHz), δ(ppm) = -65.66, -65.63.

ESI-HRMS (m/z): calculated for $[C_{22}H_6CrF_{12}N_{14}]^-$: 746.0119; observed: 746.0125.

Elemental analysis, found: C, 44.60%; H, 4.13%; N, 20.80%. Calculated for $C_{38}H_{42}CrF_{12}N_{15}\cdot 1.5H_2O$, where the presence of crystallization water is supported by single crystal X-ray diffraction: C, 44.93%; H, 4.46%; N, 20.68%.

2.5 NMR spectra

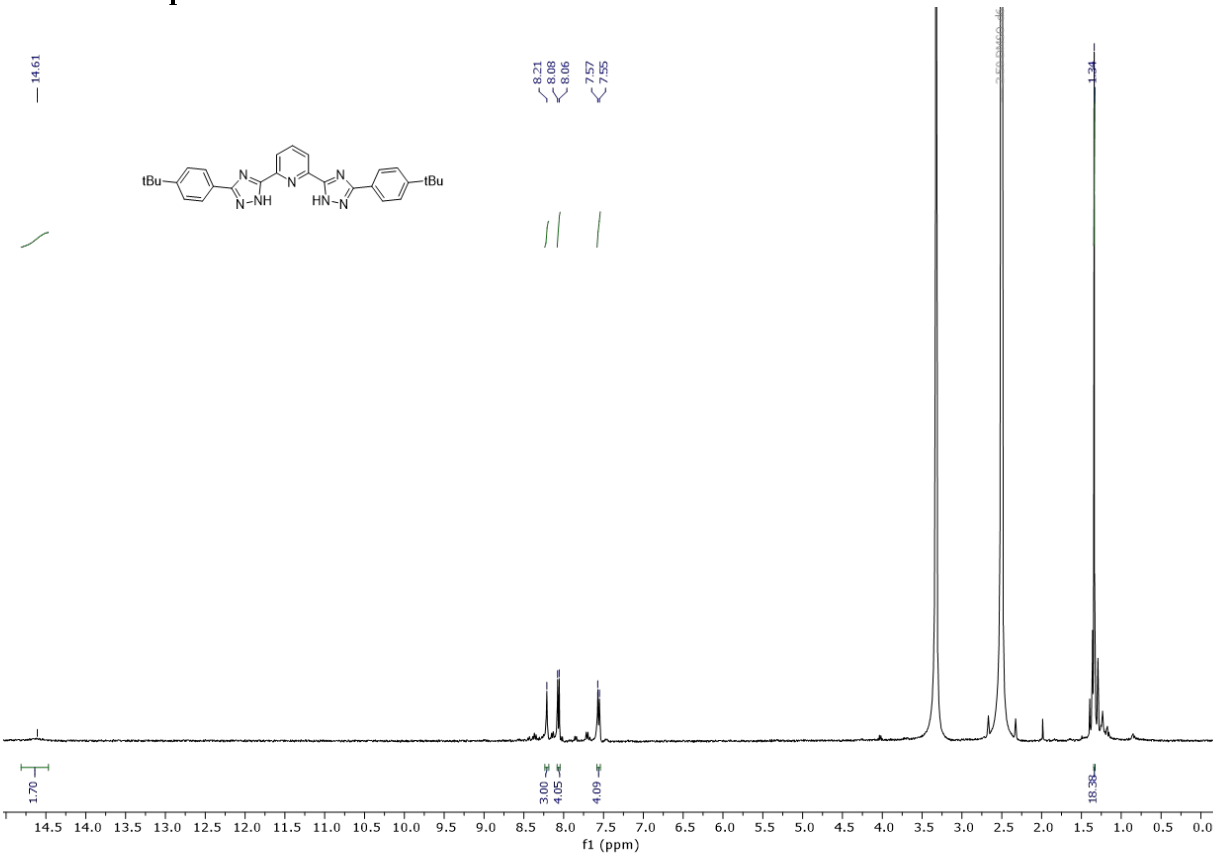

Figure S1. ^{1}H-NMR (DMSO-d_6, 400 MHz) spectrum of ^{tBuPh}BTP (2,6-bis(3-(4-(*tert*-butyl)phenyl)-1H-1,2,4-triazol-5-yl)pyridine) at 298 K, 16 scans.

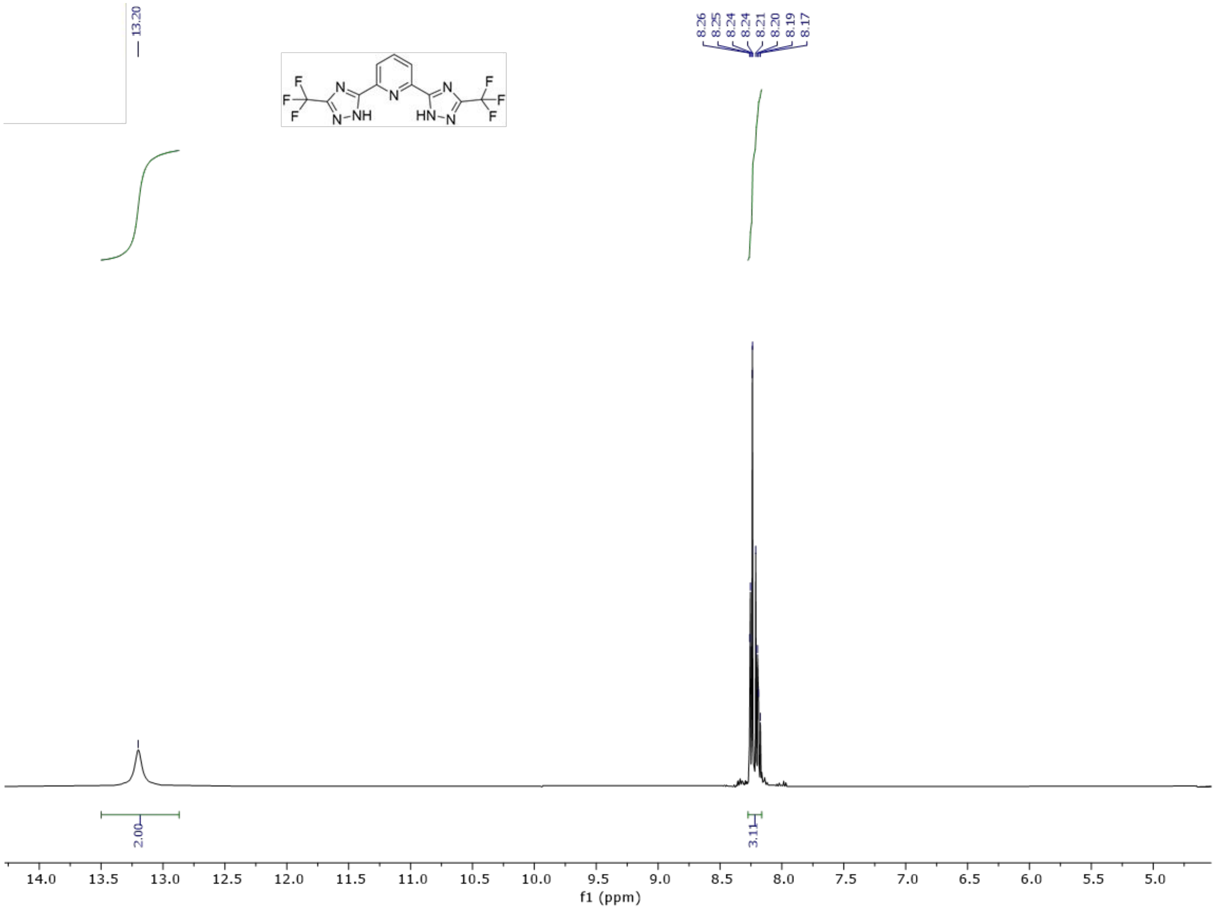

Figure S2. ^{1}H-NMR (DMSO-d$_6$, 400 MHz) spectrum of ^{CF3}BTP (2,6-bis(3-(trifluoromethyl)-1H-1,2,4-triazol-5-yl)pyridine) at 298 K, 16 scans.

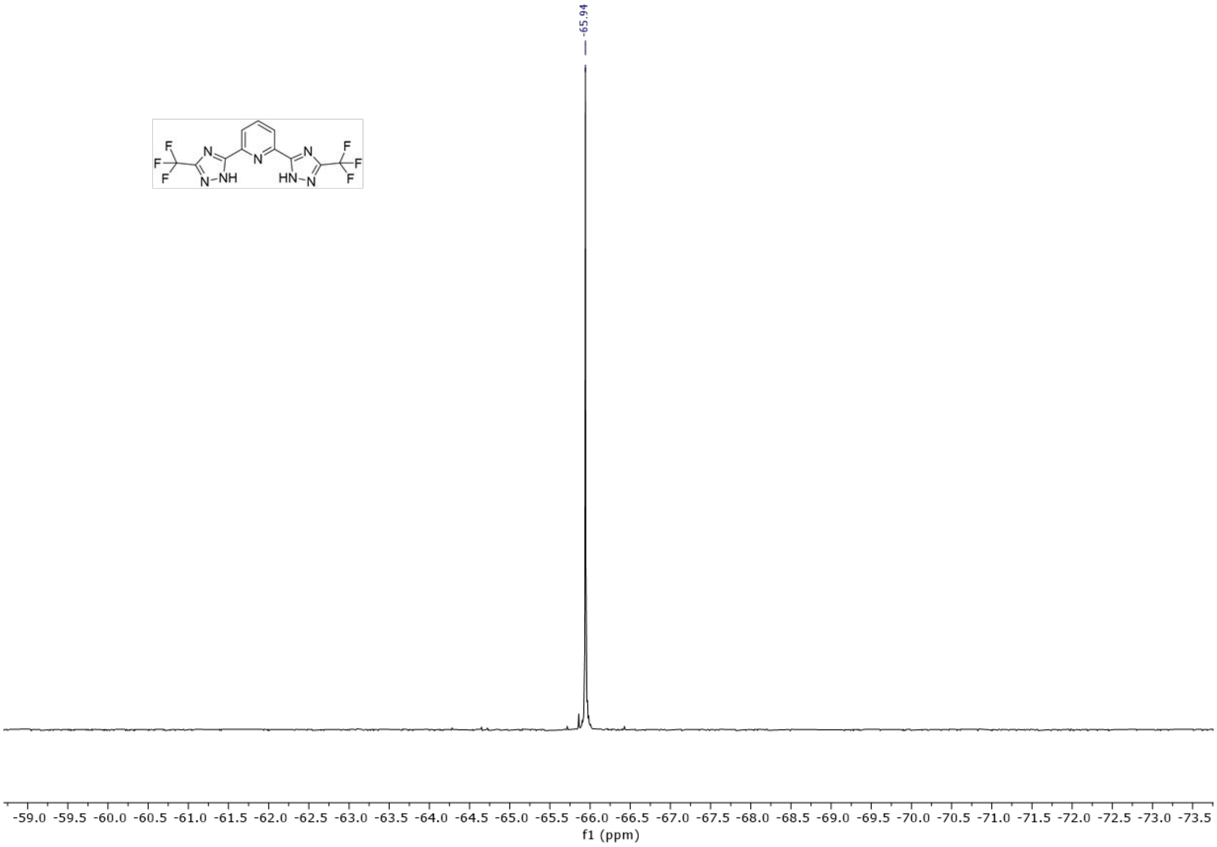

Figure S3. $^{19}F\{^{1}H\}$-NMR (DMSO-d_6, 400 MHz) spectrum of ^{CF3}BTP (2,6-bis(3-(trifluoromethyl)-1H-1,2,4-triazol-5-yl)pyridine) at 298 K, 16 scans.

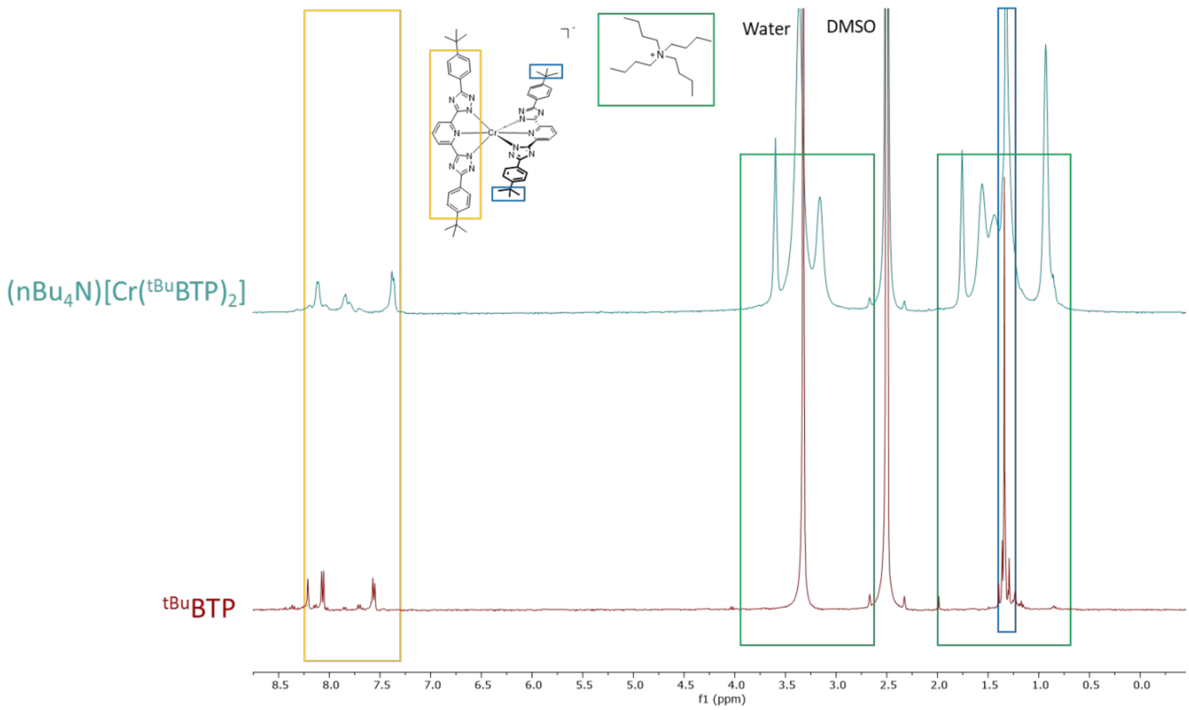

Figure S4. ^{1}H-NMR (DMSO-d_6, 400 MHz) spectrum of (top) (nBu$_4$N)[Cr(^{tBuPh}BTP)$_2$] and (bottom) ^{tBuPh}BTP (2,6-bis(3-(4-(*tert*-butyl)phenyl)-1H-1,2,4-triazol-5-yl)pyridine) at 298 K, 16 scans. The characteristic peaks are highlighted to show the difference in the chemical shift and signal broadening in the paramagnetic Cr^{III} species. Due to the presence of the paramagnetic species that produces a broadening of the signals and the overlap between the signals from the solvent and the product, the integration is not accurate and coupling constants are not provided.

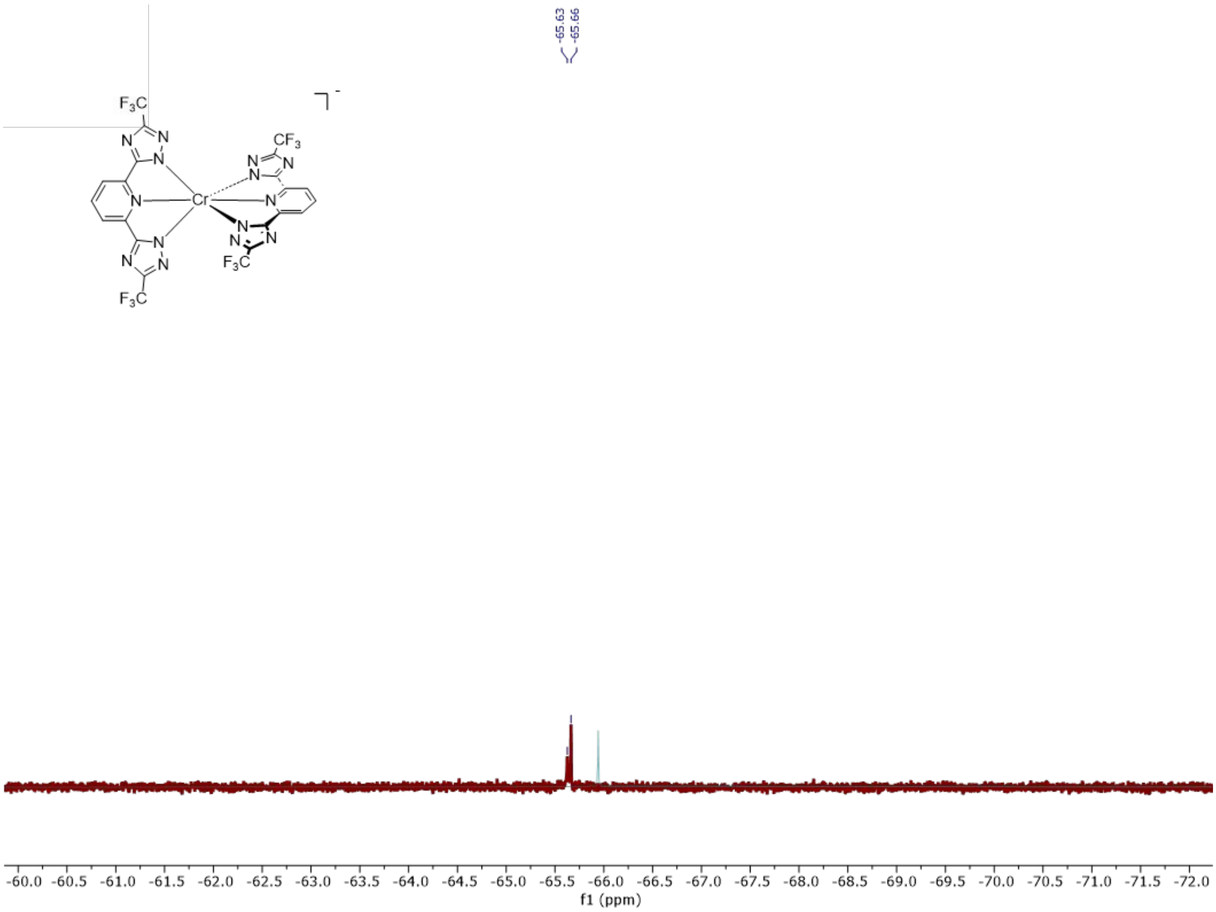

Figure S5. $^{19}F\{^{1}H\}$-NMR (DMSO-d_6, 400 MHz) spectrum of ($n$$Bu_4$N)[Cr(^{CF3}BTP)$_2$] (red line) compared to the one of ^{CF3}BTP (teal line) at 298 K, 16 scans.

2.6 HR-ESI mass spectra

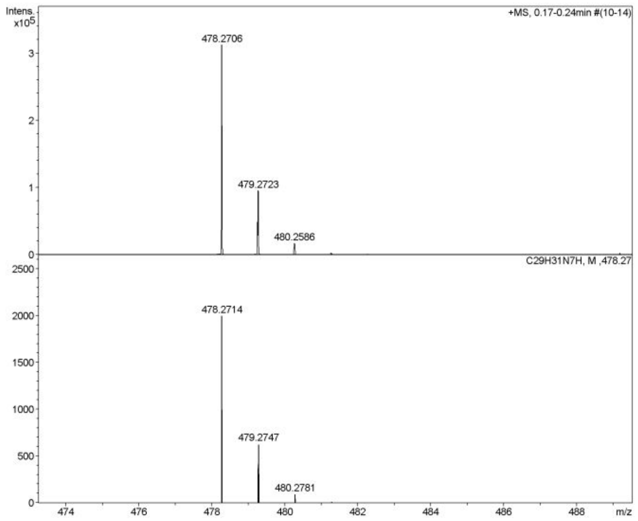

Figure S6. HRMS of ^{tBuPh}BTP (2,6-bis(3-(4-(*tert*-butyl)phenyl)-1H-1,2,4-triazol-5-yl)pyridine). Top: measured pattern; bottom: calculated pattern for $[C_{29}H_{31}N_7+H]^+$.

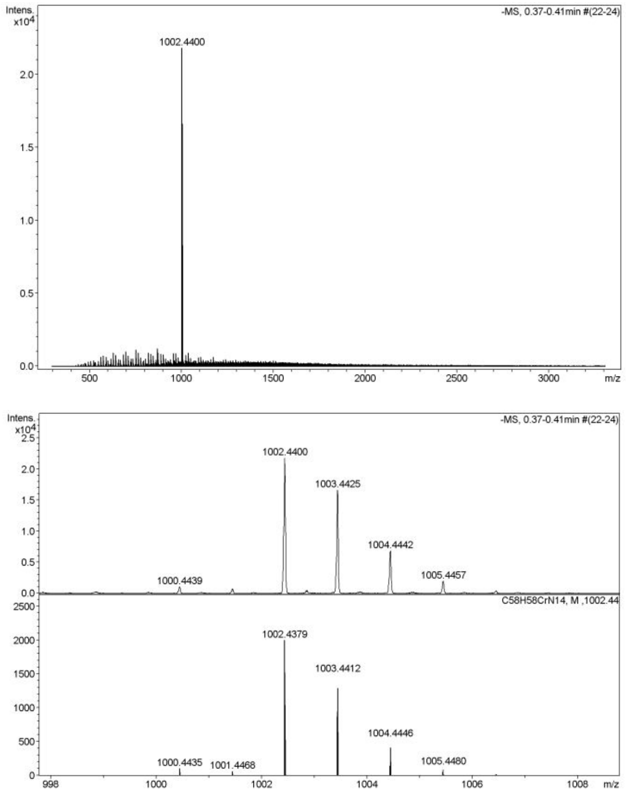

Figure S7. HRMS of $(n\mathrm{Bu}_4\mathrm{N})[\mathrm{Cr}({}^{tBuPh}\mathrm{BTP})_2]$. Top: measured pattern; middle: magnification of the measured pattern; bottom: calculated pattern for $[C_{58}H_{58}CrN_{14}]^-$.

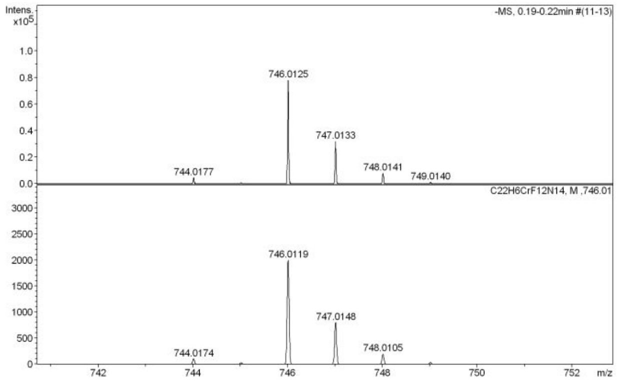

Figure S8. HRMS of (nBu$_4$N)[Cr(^{CF3}BTP)$_2$]. Top: measured pattern; bottom: calculated pattern for [$C_{22}H_6CrF_{12}N_{14}$]$^-$.

2.7 Crystal structures of (nBu$_4$N)[Cr(^{CF3}BTP)$_2$], (PPN)[Cr(^{tBuPh}BTP)$_2$] and octahedral distortion parameters

a

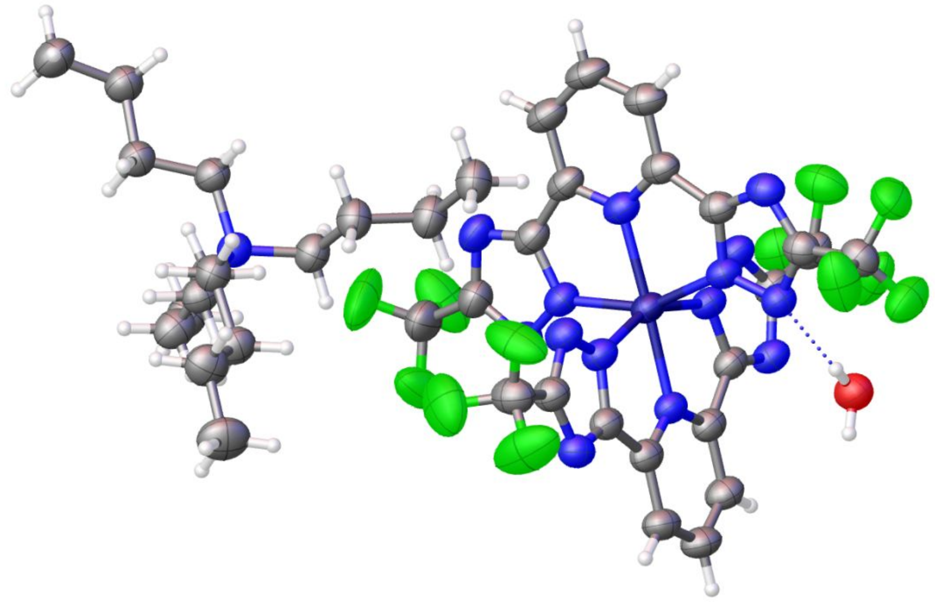

b

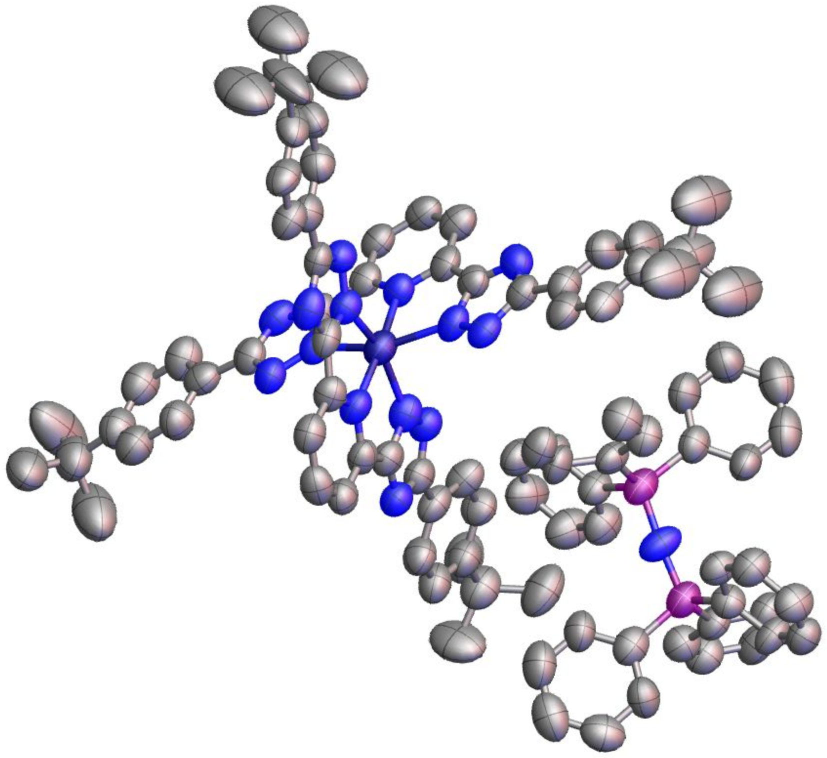

Figure S9. Solid-state X-ray crystal structure of (a) (nBu$_4$N)[Cr(^{CF3}BTP)$_2$] and (b) (PPN)[Cr(^{CF3}BTP)$_2$] (the hydrogen atoms have been removed for clarity). Thermal ellipsoids are drawn at the 50% probability level.

Single crystals of (nBu$_4$N)[Cr(^{CF3}BTP)$_2$]·H_2O were obtained by slow vapor diffusion of diethyl ether into a concentrated DCM solution. A suitable crystal was selected and mounted on a MITIGEN holder in perfluoroether oil on a STOE STADIVARI diffractometer. The crystal was kept at 150 K during data collection. Using Olex2,[5] the structure was solved with the SHELXT[6] structure solution program using Intrinsic Phasing and refined with the SHELXL[7] refinement package using Least Squares minimization.

Crystal Data for (nBu$_4$N)[Cr(^{CF3}BTP)$_2$]·H_2O (M =1006.88 g/mol): triclinic, space group P-1 (no. 2), a = 11.6695(5) Å, b = 14.9499(6) Å, c = 15.5670(6) Å, α = 62.622(3)°, β = 74.114(3)°, γ = 89.937(3)°, V =

2294.61(17) Å^3, Z = 2, T = 150 K, μ(GaKα) = 1.972 mm^{-1}, D_{calc} = 1.457 g/cm^3, 32068 reflections measured (5.846° ≤ 2ϑ ≤ 111.36°), 8788 unique (R_{int} = 0.0496, R_{sigma} = 0.0414) which were used in all calculations. The final R_1 was 0.0676 (I > 2σ(I)) and wR_2 was 0.1842 (all data).

Table S1. Crystallographic parameters for the structure of (*n*Bu$_4$N)[Cr(^{CF3}BTP)$_2$]·H_2O

Empirical formula	$C_{38}H_{44}CrF_{12}N_{15}O$
Formula weight (Da):	1006.88
Temperature/K:	150
Crystal system:	triclinic
Space group:	P-1
a/Å:	11.6695(5)
b/Å:	14.9499(6)
c/Å:	15.5670(6)
α/°:	62.622(3)
β/°:	74.114(3)
γ/°:	89.937(3)
Volume/Å^3:	2294.61(17)
Z:	2
ρ_{calc} (g/cm^3):	1.457
μ/mm^{-1}:	1.972
F(000):	1034.0
Crystal size/mm^3:	0.8 × 0.393 × 0.18
Radiation:	GaKα (λ = 1.34143 Å)
2ϑ range for data collection/°:	5.846 to 111.36
Index ranges:	-14 ≤ h ≤ 12, -15 ≤ k ≤ 18, -17 ≤ l ≤ 19
Reflections collected:	32068
Independent reflections:	8788 [R_{int} = 0.0496, R_{sigma} = 0.0414]
Data/restraints/parameters:	8788/21/665
Goodness-of-fit on F_2:	1.050
Final R indexes [I>=2σ (I)]:	R_1 = 0.0676, wR_2 = 0.1718
Final R indexes [all data]:	R_1 = 0.0764, wR_2 = 0.1842
Largest diff. peak/hole / e Å^{-3}:	0.91/-0.87

Single crystals of (PPN)[Cr(^{tBuPh}BTP)$_2$] were obtained by layering of *n*-hexane onto a concentrated solution of (PPN)[Cr(^{tBuPh}BTP)$_2$]. A suitable crystal was selected and mounted on a MITIGEN holder in perfluoroether oil on a STOE STADIVARI Cu diffractometer. The crystal was kept at 150 K during data collection. Using Olex2,[5] the structure was solved with the SHELXT[6] structure solution program using Intrinsic Phasing and refined with the SHELXL[7] refinement package using Least Squares minimization.

Crystal Data for (PPN)[Cr(^{tBuPh}BTP)$_2$] (M = 1541.63 g/mol): monoclinic, space group P2$_1$/n (no. 14), a = 16.6483(5) Å, b = 34.4672(8) Å, c = 34.2036(11) Å, β = 101.203(2), V = 19252.7(10) Å^3, Z = 8, T = 150 K, μ(Cu Kα) = 2.949 mm^{-1}, D_{calc} = 1.210 g/cm^3, 185537 reflections measured (9.33° ≤ 2ϑ ≤ 134.998°), 34583 unique (R_{int} = 0.1468, R_{sigma} = 0.1019) which were used in all calculations. The final R_1 was 0.1120 (I > 2σ(I)) and wR_2 was 0.2892 (all data).

Table S2. Crystallographic parameters for the structure of (PPN)[Cr(^{tBuPh}BTP)$_2$]

Empirical formula	$C_{94}H_{88}CrN_{15}P_2$
Formula weight (Da):	1541.63
Temperature/K:	150
Crystal system:	monoclinic
Space group:	P2$_1$/n
a/Å:	16.6483(5)
b/Å:	34.4672(8)
c/Å:	34.2036(11)

α/°:	90
β/°:	101.203(2)
γ/°:	90
Volume/Å^3:	19252.7(10)
Z:	8
ρ_{calc} (g/cm^3):	1.210
μ/mm^{-1}:	2.949
F(000):	7343.0
Crystal size/mm^3:	0.6 × 0.467 × 0.4
Radiation:	Cu Kα (λ = 1.54186)
2ϑ range for data collection/°:	9.33 to 134.998
Index ranges:	-17 ≤ h ≤ 19, -41 ≤ k ≤ 21, -40 ≤ l ≤ 40
Reflections collected:	185537
Independent reflections:	34583 [R_{int} = 0.1468, R_{sigma} = 0.1019]
Data/restraints/parameters:	34583/471/2017
Goodness-of-fit on F_2:	1.080
Final R indexes [I>=2σ (I)]:	R_1 = 0.1120, wR_2 = 0.2368
Final R indexes [all data]:	R_1 = 0.2361, wR_2 = 0.2892
Largest diff. peak/hole / e Å^{-3}:	1.06/-0.35

The distortion parameters, Δ, Σ and Θ, where obtained from OctaDist.[8] As an input file, the xyz cartesian coordinates of the atoms involved in the octahedron (the central chromium and the six coordinated nitrogen atoms) were used. The software computed the values according to the following equations:

$$\Delta = \frac{1}{6}\sum_{i=1}^{6}\left(\frac{d_i - d}{d}\right)^2 \quad \text{(S1)}$$

$$\Sigma = \sum_{i=1}^{12}|\phi_i - 90°| \quad \text{(S2)}$$

$$\Theta = \sum_{i=1}^{24}|\theta_i - 60°| \quad \text{(S3)}$$

Where d_i are the metal-ligand bond lengths around the central ion, d the average distance, φ_i are the *cis* ligand-metal-ligand angles, and θ_i are the torsional angles. In particular, Δ quantifies the deviation of each metal–ligand bond length from the average bond length. Σ represents the sum of the deviations of the 12 *cis* bond angles (ideally 90°) around the metal center. Θ captures the overall angular deviation from an ideal octahedral geometry, accounting for both angular and torsional distortions. All these parameters are zero in a perfect octahedron and increase with greater distortion. Since the geometric meaning of Θ is less intuitive, we refer interested readers to the original reference for further details.[8]

Table S3: Distortion parameters for selected Cr^{III} octahedral complexes with monodentate, bidentate and tridentate ligands.

	Δ	Σ / °	Θ / °
$[Cr(NH_3)_6]^{3+}$ [a]	0.000028	18.247594	55.557583
$[Cr(bpy)_3]^{3+}$ [b]	0.000056	66.554768	206.451409
$[Cr(dqp)_2]^{3+}$ [c]	0.000646	30.894682	105.106083
$[Cr(ddpd)_2]^{3+}$ [d]	0.000020	36.624643	122.453855
$[Cr(tpy)_2]^{3+}$ [e]	0.004869	121.604086	369.949484
$[Cr(^{CF3}BTP)_2]^{-}$ [f]	0.000018	107.307095	357.392885
$[Cr(^{tBuPh}BTP)_2]^{-}$ [f]	0.000277	114.185908	369.091704

The crystal structures from where the data were extrapolated are: [a] ref [9], [b] ref [10], [c] ref [11], [d] ref [12], [e] ref [13], [f] this work.

3 DFT calculations

All calculations were performed using the Gaussian16 software.[14] The B3LYP[15,16] hybrid density functional was used for all geometry optimizations and single-point calculations on the optimized structure. The def2-svp basis set was applied to H, C and F and the more accurate def2-tzvp basis set was used for the atoms Cr and N.[17] The solvent effect for acetonitrile was modelled through the integral equation formalism model (IEFPCM) for all calculations.

The analysis of the excited states was done through MultiWFN[18] with the intrafragment charge transfer analysis through the Muliken population. The natural transition orbitals (NTO) were visualized with an isosurface value of 0.05 through Chemcraft.[19]

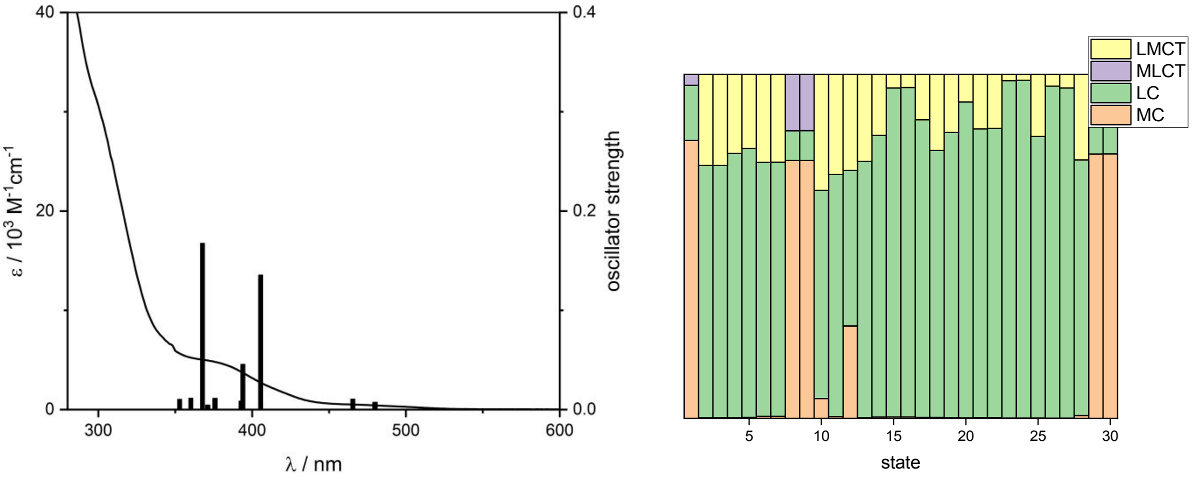

Figure S10. Calculated and experimentally obtained UV-Vis absorption spectra of the $[Cr(^{tBuPh}BTP)_2]^-$ complex (left). The experimental UV-Vis absorption spectrum (black trace) was measured in acetonitrile at 20 °C. The vertical black bars indicate the energies of the calculated excited states, with their heights representing the oscillator strengths. Metal-centered transitions, being weak, fall below the significant digit threshold and are therefore not prominently displayed. The interfragment charge transfer analysis (right) calculated for $[Cr(^{tBuPh}BTP)_2]^-$. The net values for the LMCT, MLCT, LC and MC transitions were calculated.

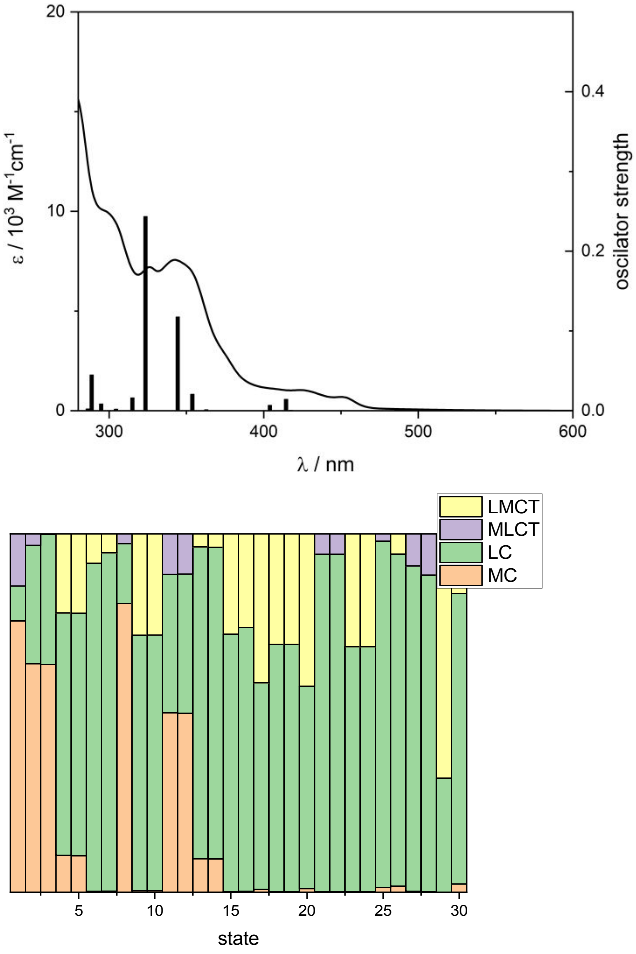

Figure S11. Calculated and experimentally obtained UV-Vis absorption spectra of [Cr(^{CF3}BTP)$_2$]$^-$ (left). The experimental UV-Vis absorption spectrum (black trace) measured in acetonitrile at 20 °C. The vertical black bars indicate the energies of the calculated excited states, with their heights representing the oscillator strengths. Metal-centered transitions, being weak, fall below the significant digit threshold and are therefore not prominently displayed. The interfragment charge transfer analysis (right) calculated for [Cr(^{CF3}BTP)$_2$]$^-$. The net values for the LMCT, MLCT, LC and MC transitions were calculated.

Table S4. Main natural transition orbitals of the first ten excited states of [Cr(^{tBuPh}BTP)$_2$]$^-$.

state	excitation energy	oscillator strength	major natural transition orbitals<br>*hole* ⟶ *electron*	assignment
1	498.4 nm (2.49 eV)	0.0000	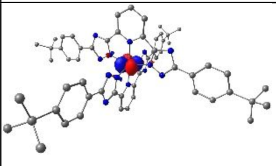 ⟶ 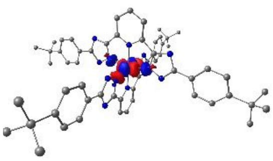	MC
2	480.0 nm (2.58 eV)	0.0076	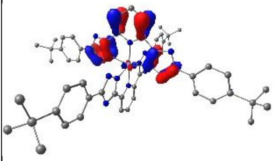 ⟶ 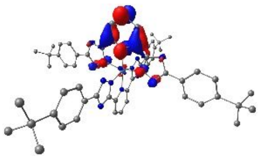	LC
3	479.7 nm (2.58 eV)	0.0075	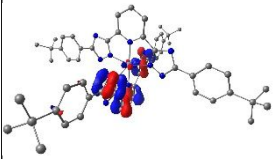 ⟶ 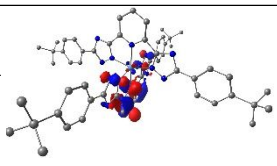	LC

4	465.5 nm (2.66 eV)	0.0108	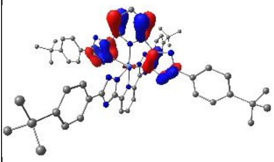 → 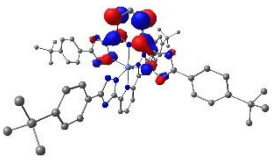	LC
5	461.6 nm (2.68 eV)	0.0000	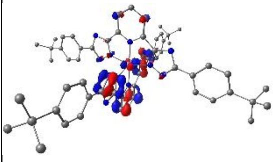 → 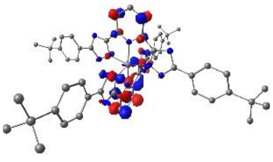	LC
6	429.9 nm (2.88 eV)	0.0001	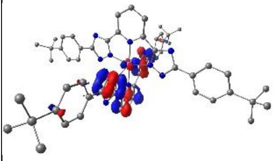 → 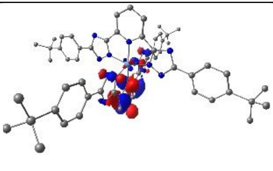	LC
7	429.7 nm (2.89 eV)	0.0002	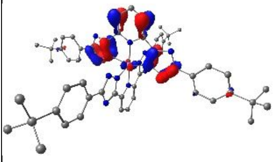 → 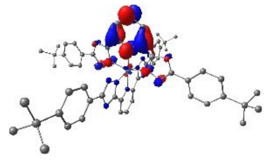	LC
8	423.0 nm (2.93 eV)	0.0006	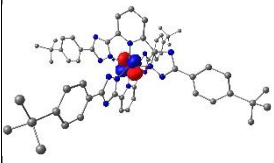 → 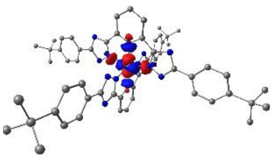	MC
9	423.0 nm (2.93 eV)	0.0006	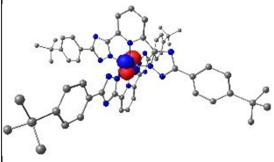 → 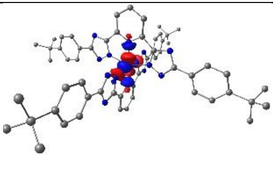	MC
10	422.1 nm (2.94 eV)	0.0000	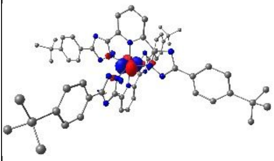 → 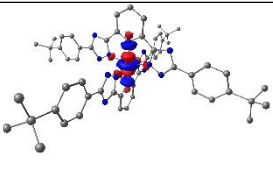	LC/(LMCT)

Table S5. Main natural transition orbitals of the first ten excited states of $[Cr(^{CF3}BTP)_2]^-$.

state	excitation energy	oscillator strength	major natural transition orbitals *hole* → *electron*	assignment
1	482.2 nm (2.57 eV)	0.0000	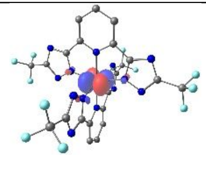 → 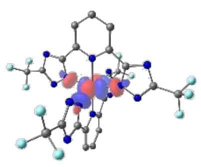	MC
2	417.5 nm (2.97 eV)	0.0007	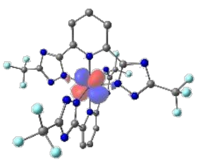 → 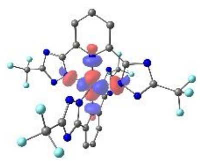	MC/(LC)

3	417.5 nm (2.97eV)	0.0007	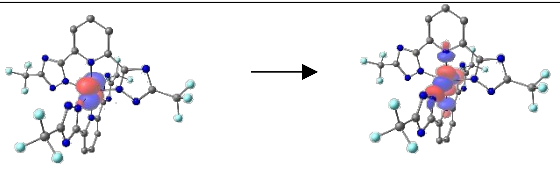	MC/(LC)
4	414.5 nm (2.99 eV)	0.0144	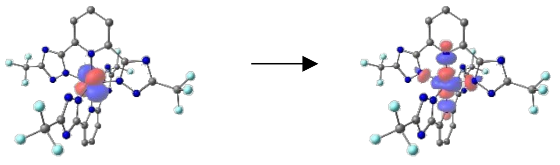	LC
5	414.4 nm (2.99 eV)	0.0144	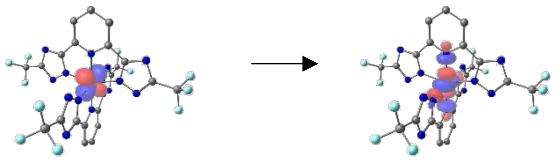	LC
6	404.0 nm (3.07 eV)	0.0069	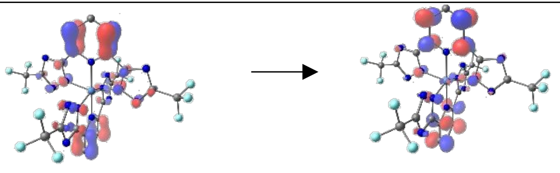	LC
7	401.0 nm (3.09 eV)	0.0000	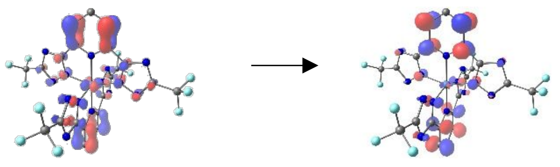	LC
8	397.0 nm (3.12 eV)	0.0000	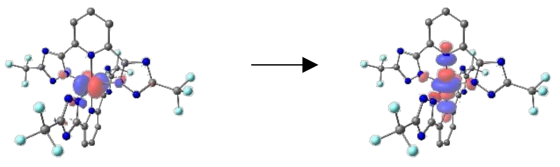	MC
9	362.9 nm (3.42 eV)	0.0014	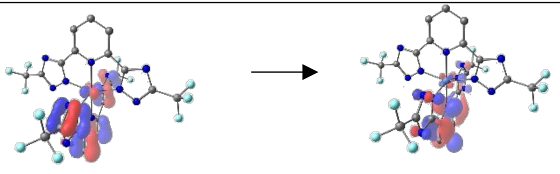	LC
10	362.9 nm (3.42 eV)	0.0014	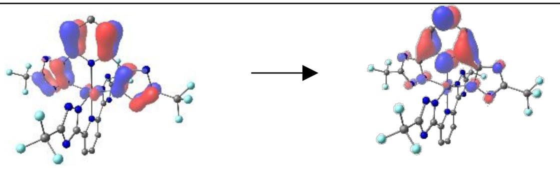	LC

Table S6. XYZ cartesian coordinates from the calculated ground state optimization of [Cr(^{tBuPh}BTP)$_2$]$^-$

Cr	0.0019120	-0.0027240	0.0081320
N	0.0044290	0.0056400	2.0670690
N	0.4227490	1.9679530	0.4496310
N	-0.4181560	-1.9694830	0.4666100
N	0.7607800	3.5424580	1.9892950
N	-0.0004310	-0.0118800	-2.0507800
N	-0.6614110	-3.1073430	-0.1966190
N	-0.7525290	-3.5315170	2.0198370
N	0.6647210	3.1002380	-0.2234020
N	1.9702790	-0.4251870	-0.4422580
C	0.4851190	2.2583170	1.7696040
N	3.5373360	-0.7656440	-1.9889270

N	3.1059680	-0.6648850	0.2257710
C	-0.4766870	-2.2494460	1.7890120
C	0.2514610	1.1619530	2.7058950
C	-0.2406300	-1.1455460	2.7158570
N	-1.9671860	0.4162930	-0.4413840
N	-3.5377920	0.7433210	-1.9873910
N	-3.1011900	0.6627710	0.2270830
C	0.8637250	4.0330390	0.7251530
C	-0.8599210	-4.0319920	0.7599650
C	0.2613200	1.2036620	4.1033620
H	0.4618790	2.1411060	4.6217690
C	1.1525210	-0.2611270	-2.6948130
C	0.0081080	0.0166190	4.7993120
H	0.0095060	0.0210150	5.8913860
C	-0.2468810	-1.1760200	4.1135950
H	-0.4461080	-2.1092560	4.6400440
C	2.2538570	-0.4914970	-1.7635050
C	-0.0037430	-0.0247660	-4.7830070
H	-0.0050790	-0.0299120	-5.8750790
C	-2.2539130	0.4707490	-1.7625290
C	4.0337530	-0.8673790	-0.7269610
C	1.1869580	-0.2755240	-4.0924320
H	2.1216940	-0.4778350	-4.6150340
C	-1.1549500	0.2313350	-2.6943430
C	-1.1927720	0.2325610	-4.0919260
H	-2.1287500	0.4300010	-4.6141650
C	-4.0311260	0.8569020	-0.7252480
C	5.4427710	-1.1670000	-0.4230990
C	6.3760500	-1.3722630	-1.4555660
C	5.9018120	-1.2570240	0.9006460
C	7.7098060	-1.6552260	-1.1670920
H	6.0465730	-1.3076660	-2.4941980
C	7.2409280	-1.5409800	1.1807200
H	5.2006170	-1.1025270	1.7226590
C	8.1819830	-1.7479740	0.1573560
H	8.3978720	-1.8072170	-2.0021410
H	7.5435460	-1.5990920	2.2265010
C	1.1635750	5.4407680	0.4152790
C	1.3588000	6.3803650	1.4439730
C	1.2648560	5.8921370	-0.9102720
C	1.6422240	7.7128370	1.1501980
H	1.2860360	6.0567380	2.4838740
C	1.5488850	7.2301530	-1.1956630
H	1.1195140	5.1858000	-1.7295530
C	1.7450540	8.1776470	-0.1761320
H	1.7860020	8.4059210	1.9825270
H	1.6162360	7.5265920	-2.2426490
C	-5.4392920	1.1601090	-0.4210740
C	-6.3763000	1.3476570	-1.4535050
C	-5.8936400	1.2719990	0.9026430
C	-7.7093230	1.6340100	-1.1650920
H	-6.0502820	1.2664030	-2.4920550
C	-7.2321040	1.5591910	1.1826470
H	-5.1892410	1.1322870	1.7245980
C	-8.1769990	1.7479370	0.1592810
H	-8.4004820	1.7715760	-2.0000790
H	-7.5311520	1.6344060	2.2283720
C	-1.1635300	-5.4412830	0.4615760
C	-1.3502390	-6.3737430	1.4934160
C	-1.2784940	-5.8991550	-0.8644740
C	-1.6398710	-7.7122120	1.2119920

H	-1.2672720	-6.0458040	2.5312900
C	-1.5675180	-7.2344090	-1.1356690
H	-1.1397440	-5.1965090	-1.6879880
C	-1.7566730	-8.1803260	-0.1074800
H	-1.7754760	-8.3939510	2.0519680
H	-1.6474610	-7.5432780	-2.1806350
C	2.0593350	9.6600660	-0.4547070
C	0.9537950	10.5446550	0.1680550
H	0.8807320	10.4053600	1.2574760
H	1.1653960	11.6103000	-0.0189130
H	-0.0305480	10.3118280	-0.2689800
C	3.4229610	10.0238940	0.1781110
H	4.2328250	9.4118620	-0.2505190
H	3.6617610	11.0836370	-0.0098810
H	3.4245800	9.8700970	1.2679760
C	2.1304690	9.9673520	-1.9614330
H	2.9210790	9.3872360	-2.4626140
H	1.1767240	9.7563240	-2.4700180
H	2.3568140	11.0346230	-2.1119610
C	9.6649200	-2.0643940	0.4301280
C	10.5503710	-0.9693350	-0.2095440
H	11.6160960	-1.1832420	-0.0257810
H	10.4060460	-0.9076870	-1.2989850
H	10.3238790	0.0207470	0.2177770
C	10.0187280	-3.4362210	-0.1907180
H	11.0788890	-3.6774090	-0.0081490
H	9.4065760	-4.2389660	0.2509170
H	9.8564930	-3.4484090	-1.2793310
C	9.9810080	-2.1207230	1.9356600
H	9.7769190	-1.1606540	2.4351290
H	9.4007060	-2.9034530	2.4488280
H	11.0481810	-2.3501140	2.0821870
C	-9.6593320	2.0673830	0.4317060
C	-10.5457240	0.9611440	-0.1870890
H	-11.6111140	1.1774280	-0.0041850
H	-10.4041740	0.8812580	-1.2757080
H	-10.3174520	-0.0214350	0.2562930
C	-10.0165270	3.4285310	-0.2103440
H	-11.0761990	3.6719180	-0.0278680
H	-9.4033890	4.2389100	0.2157040
H	-9.8582870	3.4228810	-1.2996150
C	-9.9705800	2.1480250	1.9371410
H	-9.7612000	1.1973000	2.4520600
H	-9.3915030	2.9414610	2.4350160
H	-11.0381070	2.3759430	2.0834200
C	-2.0765700	-9.6468160	-0.4551190
C	-2.2443740	-10.5199540	0.8015440
H	-2.4718120	-11.5561820	0.5057270
H	-1.3277470	-10.5433810	1.4115170
H	-3.0707260	-10.1688170	1.4392550
C	-0.9259030	-10.2365450	-1.3039260
H	-1.1404320	-11.2863790	-1.5630700
H	-0.7836570	-9.6835160	-2.2449070
H	0.0262740	-10.2111110	-0.7499750
C	-3.3928320	-9.7066380	-1.2652390
H	-4.2337420	-9.2977520	-0.6822910
H	-3.3242070	-9.1360000	-2.2039570
H	-3.6351890	-10.7500410	-1.5258540

Table S7. XYZ cartesian coordinates from the calculated ground state optimization of $[Cr(^{CF3}BTP)_2]^-$

Cr	0.0019680	-0.0009770	0.0001130
N	-0.0016260	-0.0047880	-2.0509900
F	5.1694130	-3.5382230	-0.9452010
F	-4.0795610	-4.1480430	-0.8747200
N	-1.4315900	1.4089290	-0.4434630
F	-3.5748280	-5.1503260	0.9801890
F	4.1393130	-4.0944610	0.8792090
N	1.4350370	-1.4114760	-0.4433580
N	-2.5790470	2.5343460	-1.9879040
N	0.0042400	-0.0001290	2.0512080
F	-5.1062880	-3.6267390	0.9618510
N	2.2632010	-2.2214140	0.2311960
F	3.6683440	-5.0912160	-0.9874970
N	2.5764360	-2.5432970	-1.9875530
N	-2.2552810	2.2235250	0.2309180
N	-1.4085030	-1.4330870	0.4460640
C	-1.6432720	1.6127840	-1.7653930
N	-2.5368960	-2.5757190	1.9918410
N	-2.2220670	-2.2585360	-0.2273120
C	1.6408910	-1.6214640	-1.7652170
C	-0.8426590	0.8206990	-2.6984930
C	0.8354680	-0.8343950	-2.6984320
N	1.4126980	1.4325880	0.4411750
N	2.5438060	2.5767440	1.9838980
N	2.2242420	2.2583630	-0.2344730
C	-2.9174290	2.8722180	-0.7261790
C	2.9215680	-2.8741070	-0.7258130
C	-0.8732380	0.8433120	-4.0942940
H	-1.5557690	1.5136600	-4.6164150
C	-0.8228800	-0.8385070	2.7001300
C	-0.0097080	-0.0130990	-4.7862050
H	-0.0129620	-0.0164970	-5.8779830
C	0.8578710	-0.8653950	-4.0941850
H	1.5372040	-1.5390380	-4.6162430
C	-1.6144390	-1.6410950	1.7682270
C	-3.9104090	-3.9630050	0.4411080
C	0.0084130	0.0004860	4.7864460
H	0.0100850	0.0007390	5.8782340
C	1.6216560	1.6412260	1.7627810
C	-2.8730910	-2.9172780	0.7305240
C	-0.8475670	-0.8646460	4.0959650
H	-1.5192500	-1.5449660	4.6192600
C	0.8332550	0.8386110	2.6972540
C	3.9765310	-3.9019690	-0.4362640
C	0.8622350	0.8653850	4.0929820
H	1.5354180	1.5460630	4.6138810
C	2.8762200	2.9186560	0.7217330
C	-3.9722270	3.9001800	-0.4363070
C	3.9014330	3.9759110	0.4310530
F	3.5209580	5.1749260	0.9131440
F	5.0830970	3.6859510	1.0074250
F	-4.1223550	4.1052490	0.8787800
F	-5.1691990	3.5283860	-0.9293650
F	-3.6728310	5.0847130	-1.0024840
F	4.1143560	4.1180140	-0.8836290

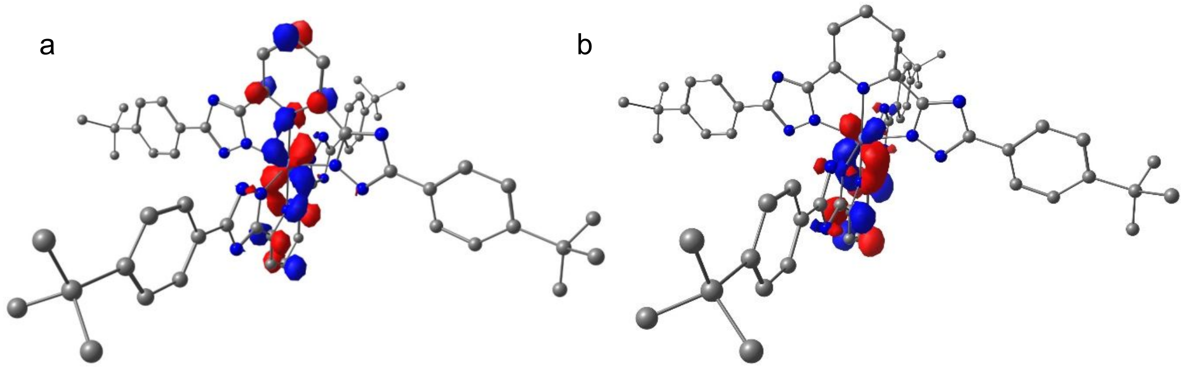

Figure S12. Visualizations of the β-spin molecular orbitals involved in the reduction process: (a) the singly occupied molecular orbital (SOMO β, corresponding to the lowest unoccupied β-spin orbital) of $[Cr(^{tBuPh}BTP)_2]^-$ and (b) the highest occupied orbital (HOMO β) of the one-electron reduced species $[Cr(^{tBuPh}BTP)_2]^{2-}$. These orbital visualizations illustrate the character of the orbital accepting the additional electron. The metal contributions are 32.9% and 30.2%, respectively, supporting a predominantly ligand-centered reduction.

4 Determination of 10 Dq, B and β

The ligand-field splitting parameter (10 Dq) for $[Cr(^{tBuPh}BTP)_2]^-$ was estimated from the absorption band at 498 nm (20,080 cm^{-1}), which DFT calculations (see above) assigned a strong MC character.

In the same way, for $[Cr(^{CF3}BTP)_2]^-$, this was estimated to be at 452nm (22,100 cm^{-1}).

The calculation of the Racah B parameter was performed using the following equation:[20]

$$E(^2E) = 9B + 3C - 50\left(\frac{B^2}{10Dq}\right) \qquad \text{(S4)}$$

Where $E(^2E)$ is the energy of the photoactive excited state estimated from the short wavelength side of the emission band, where the intensity equals 10% of the intensity at the maximum, i.e. 820 nm (12,200 cm^{-1}) for $[Cr(^{tBuPh}BTP)_2]^-$ and 780 nm (12,820 cm^{-1}) for $[Cr(^{CF3}BTP)_2]^-$.

The Racah *C* parameter that further takes into account the interelectronic repulsions was assumed to be C = 4·B following textbooks, though recent work demonstrated that the C / B ratio can be substantially higher in some cases.[21]

On this basis, we obtain B = 630 cm^{-1} for $[Cr(^{tBuPh}BTP)_2]^-$ and 660 cm^{-1} for $[Cr(^{CF3}BTP)_2]^-$.

Therefore, 10 Dq /B = 32 for $[Cr(^{tBuPh}BTP)_2]^-$ and 33 for $[Cr(^{CF3}BTP)_2]^-$.

Table S8. 10 Dq / B ratios for several photoactive Cr^{III} complexes.

Complex	10 Dq / cm^{-1}	B / cm^{-1}	10 Dq / B
$[Cr(dpc)_2]^+$ [a]	19,200	550	35
$[Cr(ddpd)_2]^{3+}$ [b]	22,900	760	30
$[Cr(dqp)_2]^{3+}$ [c]	24,937	660	38
$[Cr(NH_3)_6]^{3+}$ [d]	21,600	670	32
$[Cr(^{tBuPh}BTP)_2]^-$	20,080	630	32
$[Cr(^{CF3}BTP)_2]^-$	22,100	660	33

[a] From ref [22,23]; [b] From ref [24]; [c] From ref [11]; [d] From ref [14].

The nephelauxetic parameter β was obtained by dividing B by the value of the Racah parameter B_0 of Cr^{3+} in gas phase (950 cm^{-1}).[23]

The β value resulted to be 0.66 for $[Cr(^{tBuPh}BTP)_2]^-$ and 0.69 for $[Cr(^{CF3}BTP)_2]^-$.

Table S9. Nephelauxetic parameters (β) for several Cr^{III} complexes and wavelength of the $^2E \rightarrow {}^4A_2$ emission band maximum ($\lambda_{em,\ max}$), the ^2E excited state lifetime lifetime (τ), and the ^2E emission quantum yield (Φ) in solution at room temperature, unless stated otherwise.

Complex	β [a]	$\lambda_{em,\ max}$ / nm [a]	τ / µs	Φ / %
$[Cr(dpc)_2]^+$	0.64	1067 (at 77 K)	1.4 and 6.3 [b]	Not emissive at room temperature
$[Cr(bpi^{H,H})_2]^+$	0.74	950	8.0 [c]	n.a.
fac-$[Cr(ppy)_3]$	0.75	910	9.5 [d]	0.03 [d]
$[Cr(bpi^{NMe2,H})_2]^+$	0.88	777	310 [e]	5.9 [e]
$[Cr(^{tBuPh}BTP)_2]^-$	0.66	905	23	0.1
$[Cr(^{CF3}BTP)_2]^-$	0.69	850	5	n.a.

[a] Extracted from ref [23]; [b] From ref [22], computed in MeCN; [c] From ref [25], computed in deoxygenated MeCN; [d] From ref [26], computed in 2-MeTHF; [e] From ref [27], computed in deoxygenated MeCN.

5 Further photophysical characterization

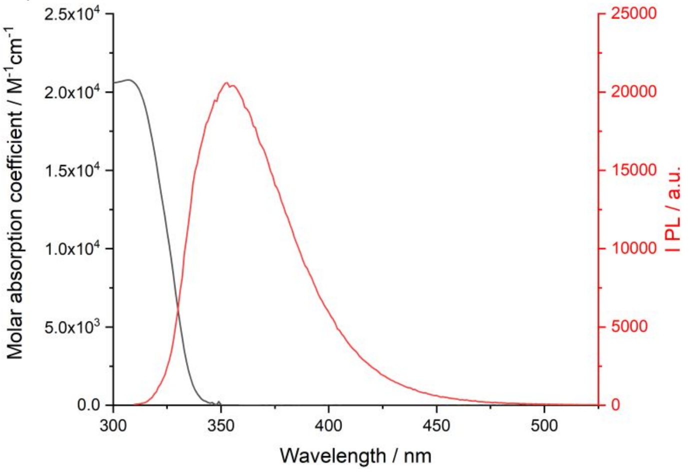

Figure S13. Absorption (black line) and emission (red line, λ_{ex} = 300 nm) spectra of ^{tBuPh}BTP in air-equilibrated 2-MeTHF at 25 °C.

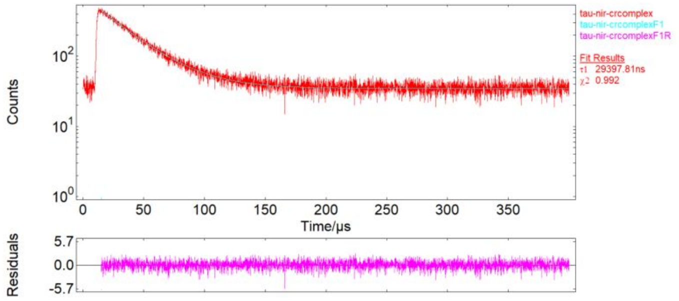

Figure S14. Emission intensity decay for (nBu$_4$N)[Cr(^{tBuPh}BTP)$_2$] in MeCN deoxygenated by 5 freeze-pump-thaw cycles (λ_{ex} = 300 nm; λ_{em} = 900 nm) at 25 °C.

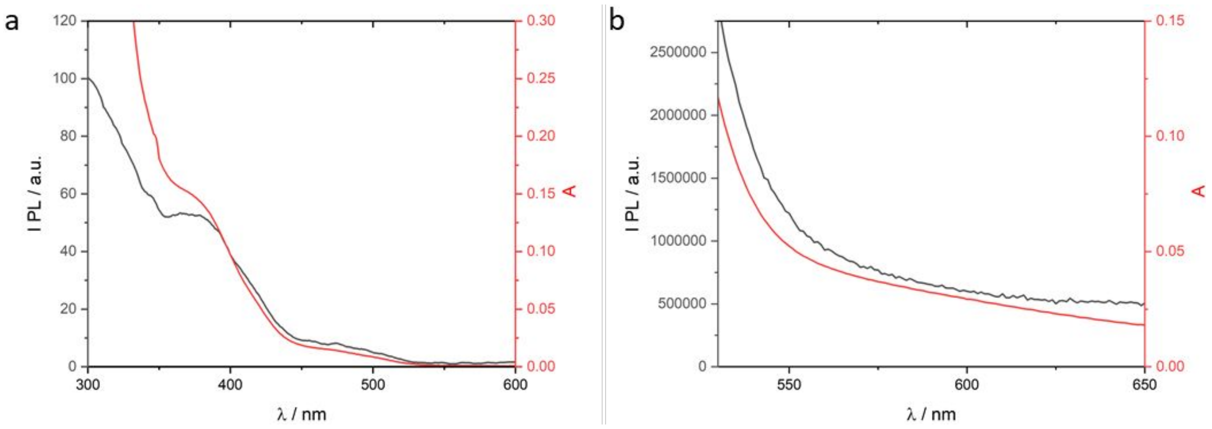

Figure S15. (a) Excitation spectrum (black line λ_{em} = 850 nm, recorded in the presence of a 645-nm cutoff filter) of (nBu$_4$N)[Cr(^{tBuPh}BTP)$_2$] in deoxygenated MeCN at 25 °C, compared to the absorption spectrum (red line). (b) Excitation spectrum collected from a more concentrated solution (black line λ_{em} = 850 nm) to show the behavior in the spectral range between 535 and 650 nm, compared to the absorption spectrum (red line) in the same range.

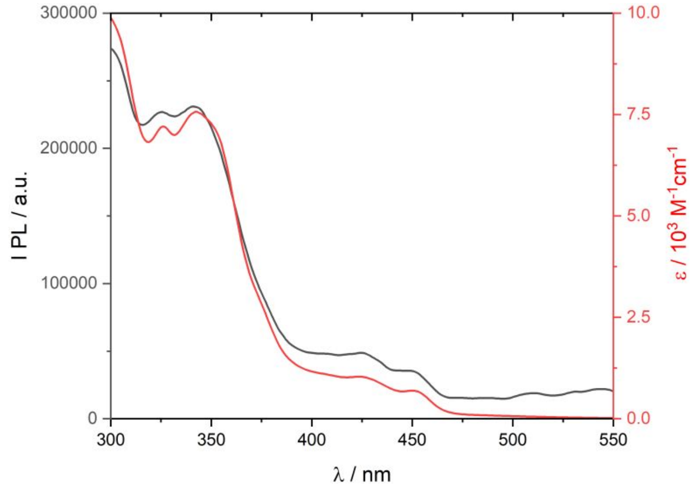

Figure S16. Excitation spectrum (black line λ_{em} = 800 nm, recorded in the presence of a 600-nm cutoff filter) of (*n*Bu$_4$N)[Cr(^{CF3}BTP)$_2$] in deoxygenated MeCN at 25 °C, compared to the absorption spectrum (red line).

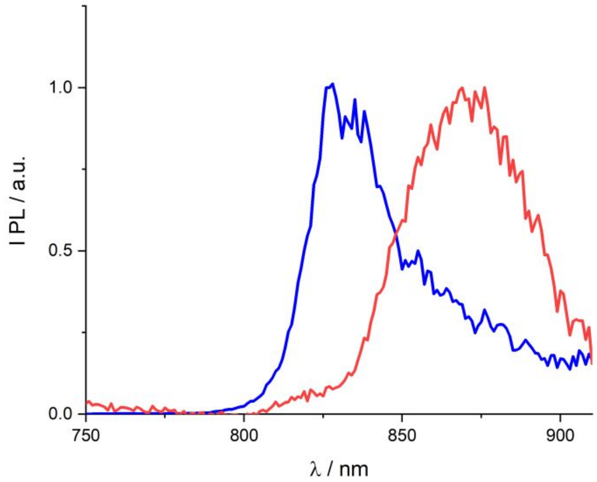

Figure S17. Emission spectra of (*n*Bu$_4$N)[Cr(^{tBuPh}BTP)$_2$] (red line, λ_{ex} = 300 nm, in the presence of a 645-nm cutoff filter) and (*n*Bu$_4$N)[Cr(^{CF3}BTP)$_2$] (blue line, λ_{ex} = 350 nm) in a frozen matrix of 2-MeTHF at 77 K.

6 Electrochemical data

6.1 Electrochemistry of (nBu$_4$N)[Cr(^{tBuPh}BTP)$_2$]

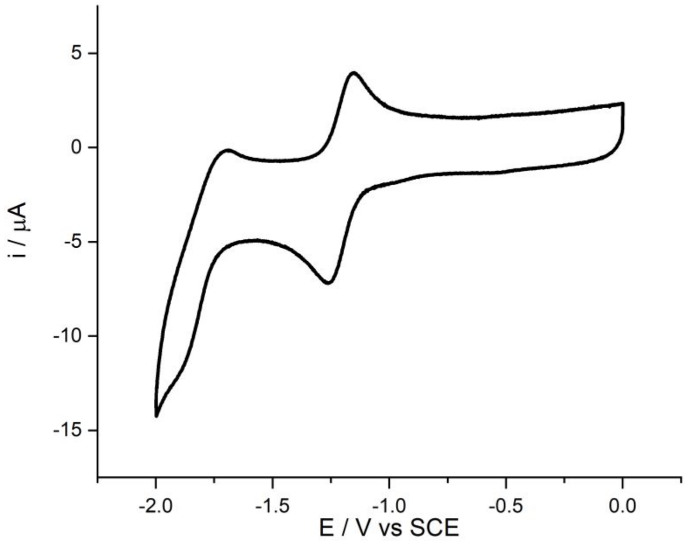

Figure S18. Cyclic voltammetry of (nBu$_4$N)[Cr(^{tBuPh}BTP)$_2$] (0.5 mM) in deoxygenated MeCN, under argon, in the presence of 0.1 M nBu$_4$PF$_6$. Scan rate: 0.2 V/s. Working electrode: glassy carbon. Counter electrode: silver wire. Reference electrode: saturated KCl calomel (SCE). Cathodic current.

A reversible reduction is seen at $E_{1/2}$ = -1.21 V vs SCE. This reduction has been attributed to the ligand, as reported for other chromium complexes.[28]

To estimate the reduction potential of the excited state ($E_{*Cr-/Cr2-}$), this formula was utilized:

$$E_{*Cr-/Cr2-} = E_{Cr-/Cr2-} + E^{00} \quad \text{(S5)}$$

Where $E_{(Cr-/Cr2-)}$ is the reduction potential of the ground state obtained from the cyclic voltammetry and E^{00} is the energy of the photoactive state, as estimated above (1.50 eV). The result is 0.29 V.

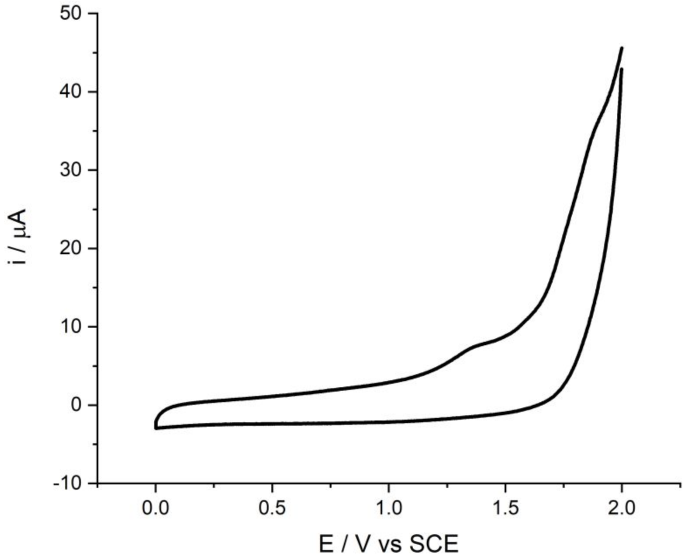

Figure S19. Cyclic voltammetry of (nBu$_4$N)[Cr(^{tBuPh}BTP)$_2$] (0.5 mM) in deoxygenated MeCN, under argon, in the presence of 0.1 M nBu$_4$PF$_6$. Scan rate: 0.2 V/s. Working electrode: glassy carbon. Counter electrode: silver wire. Reference electrode: saturated KCl calomel (SCE). Anodic current.

In the anodic regime, an irreversible oxidation is observed at +1.3 V vs SCE.

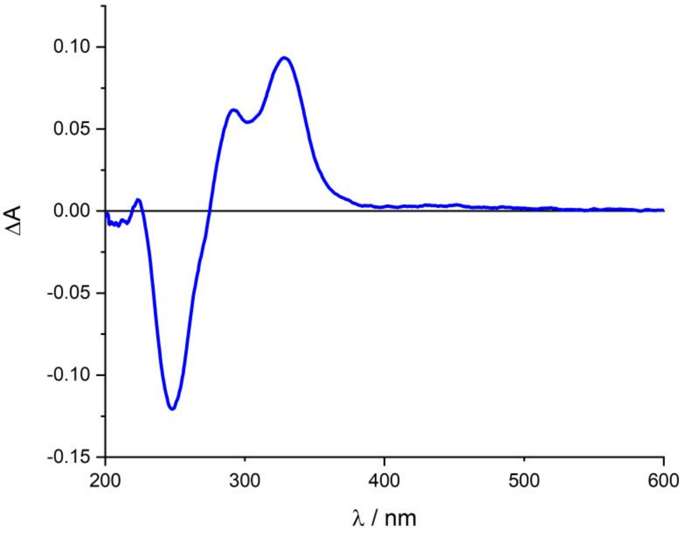

Figure S20. Spectroelectrochemistry of (nBu$_4$N)[Cr(^{tBuPh}BTP)$_2$] (1.0 mM) in deoxygenated MeCN, under argon, in the presence of 0.1 M nBu$_4$PF$_6$ collected at -1.3 V vs SCE. Integration time: 50 ms. Working electrode: platinum grid. Counter electrode: platinum wire. Reference electrode: saturated KCl calomel. The UV-Vis absorption spectrum of the initial Cr^{III} complex prior to its reduction is used as a baseline here.

6.2 Electrochemistry of (nBu$_4$N)[Cr(^{CF3}BTP)$_2$]

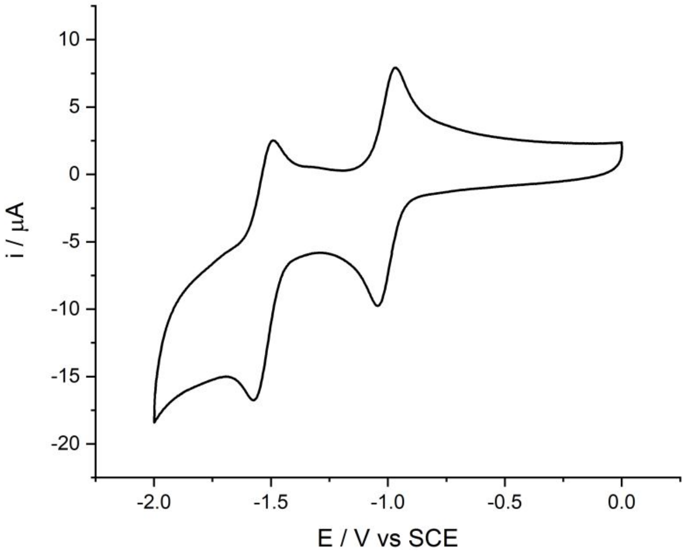

Figure S21. Cyclic voltammetry of (nBu$_4$N)[Cr(^{CF3}BTP)$_2$] (0.5 mM) in deoxygenated MeCN, under argon, in the presence of 0.1 M nBu$_4$PF$_6$. Scan rate: 0.2 V/s. Working electrode: glassy carbon. Counter electrode: silver wire. Reference electrode: saturated KCl calomel (SCE). Cathodic current.

Two reversible reductions are visible at $E_{1/2}$ = -1.00 V and -1.53 V vs SCE. The excited state reduction potential (0.59 V) has been computed with the same formula enounced in the previous paragraph, by substituting $E_{(M-/M2-)}$ with -1.00 V and E^{00} with 1.59 eV (i.e. 780 nm).

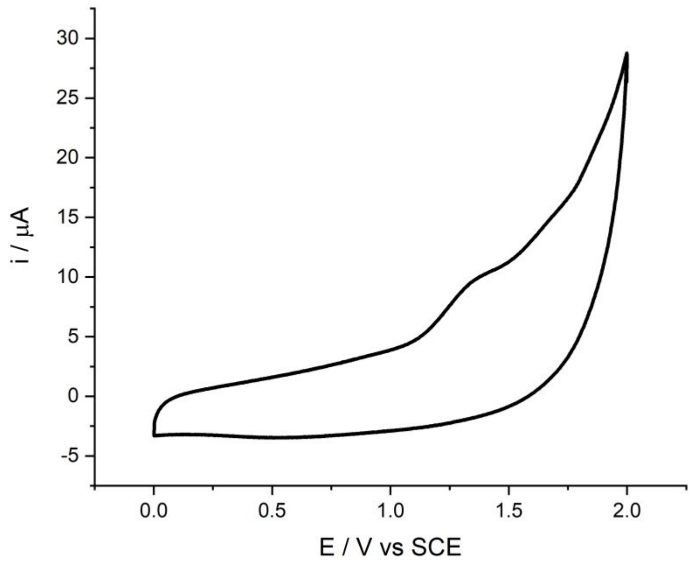

Figure S22. Cyclic voltammetry of (nBu$_4$N)[Cr(^{CF3}BTP)$_2$] (0.5 mM) in deoxygenated MeCN, under argon, in the presence of 0.1 M nBu$_4$PF$_6$. Scan rate: 0.2 V/s. Working electrode: glassy carbon. Counter electrode: silver wire. Reference electrode: saturated KCl calomel (SCE). Anodic current.

In the anodic regime, an irreversible oxidation is observed at +1.3 V vs SCE.

7 Emission quantum yield of (nBu$_4$N)[Cr(^{tBuPh}BTP)$_2$]

The emission quantum yield of (nBu$_4$N)[Cr(^{tBuPh}BTP)$_2$] in deoxygenated MeCN at 25 °C was measured following the method of Demas and Crosby[29] and using [Os(bpy)$_3$](PF$_6$)$_2$ as a reference ($\Phi_{em,R}$ = 0.5% in deoxygenated MeCN at 25 °C).[30]

Solutions of the sample and the reference were prepared so that the absorbances where in the range 0.05 and 0.1 between 450 and 500 nm. Emission spectra were collected for both solutions by exciting at 450 nm (isoabsorbing point), 480 nm and 500 nm, where the shape of the absorption spectra were similar, to minimize the error in the excitation wavelength. The spectra were collected by maintaining the same instrumental setup and by acquiring 2 summed signals to reduce the noise.

Then, the emission quantum yield Φ_{em} was determined at each excitation wavelength with the following equation:

$$\Phi_{emi} = \Phi_{em,R} \times \frac{I}{I_R} \times \frac{A_R}{A} \times \left(\frac{n}{n_R}\right)^2 \qquad \text{(S6)}$$

Where the subscript R refers to the reference, A is the absorbance at the excitation wavelength and n is the refractive index of the solvent. Since both the sample and the reference are dissolved in the same solvent, this factor cancels out.

Since the emission of the reference and the sample occur in different spectral ranges, the emission spectra were converted to wavenumbers:

- The x-axis was converted from wavelength λ (expressed in nm) into wavenumbers $\tilde{\nu}$ (in cm^{-1}) according to the formula:

$$\tilde{\nu}(cm^{-1}) = \frac{10^7}{\lambda(nm)} \qquad \text{(S7)}$$

- The y-axis was corrected as well by multiplying the square of the corresponding wavelength.[31]

$$I(\tilde{\nu}) = \lambda^2 I(\lambda) \qquad \text{(S8)}$$

The resulting emission quantum yield (0.10 ± 0.01)% is an arithmetical average of the three obtained values.

8 Singlet oxygen generation quantum yield and excited state quenching efficiency

The singlet oxygen generation quantum yield was determined by actinometry, using a solution of benzophenone in air-equilibrated MeCN as a reference. The singlet oxygen generation quantum yield obtainable with benzophenone, $\Phi(^1O_2)$, is 37%.[32] Both air-equilibrated solutions were excited at the isoabsorbing point, and singlet oxygen phosphorescence was detected in the 1230–1330 nm range. For each measurement, four spectra were accumulated. The singlet oxygen generation quantum yield was calculated using the following formula:

$$\Phi(^1O_2) = \Phi(^1O_2)_R \times \frac{I}{I_R} \tag{S9}$$

Where I is the integrated phosphorescence intensity, and the subscript R refers to the reference. The result is 70%.

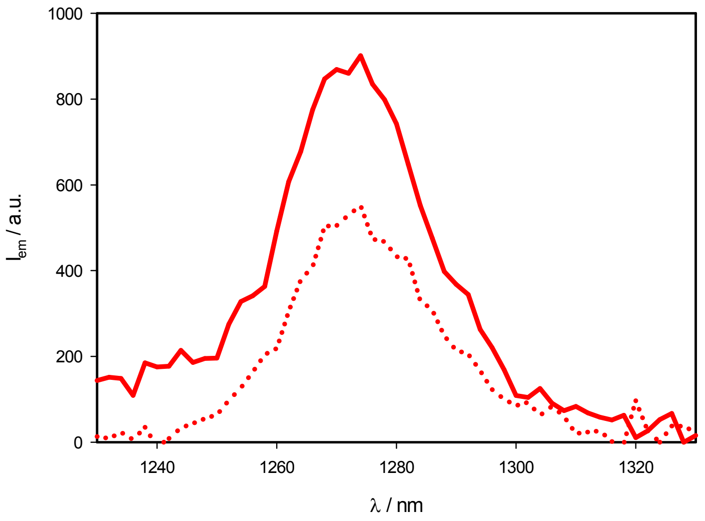

Figure S23. Singlet oxygen phosphorescence spectra resulting from Dexter energy transfer from (nBu$_4$N)[Cr(^{tBuPh}BTP)$_2$] (red solid line) and benzophenone (dotted line) in air-equilibrated MeCN at 25 °C upon excitation at 294 nm, where the absorbances of both solutions were matched.

The quenching efficiency by oxygen was calculated by measuring the $^2E/^2T_1$ excited state lifetime of a 0.33 mM air-equilibrated solution of (nBu$_4$N)[Cr(^{tBuPh}BTP)$_2$] in MeCN, by exciting at 532 nm (27 mJ).

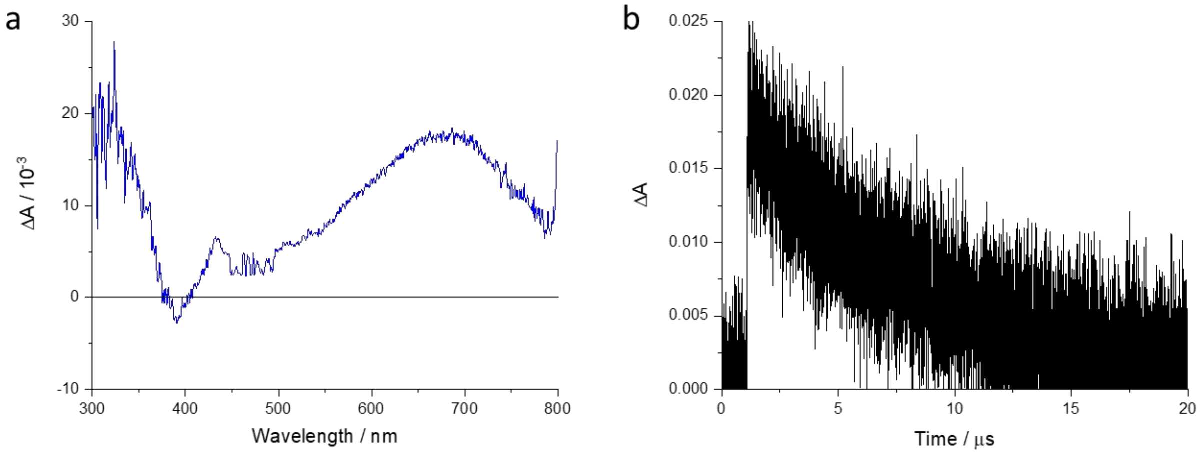

Figure S24. (a) Transient absorption spectrum of a 0.33 mM air-equilibrated solution of (nBu$_4$N)[Cr(^{tBuPh}BTP)$_2$] in MeCN, measured after excitation at 532 nm (27 mJ, 1 µs integration time, no delay) and (b) related transient absorption decay of the excited state absorption at 680 nm.

The resulting lifetime of the $^2E/^2T_1$ excited state in the presence of 1.9 mM of oxygen[30] is 7.7 µs. This implies a quenching efficiency at this oxygen concentration of:

$$\eta_q = 1 - \frac{\tau}{\tau_0} = 1 - \frac{7.7\ \mu s}{23\ \mu s} = 67\% \qquad \text{(S10)}$$

where τ_0 represents the lifetime of the doublet in the absence of quencher (deoxygenated solution) and τ is the lifetime in the presence of quencher (oxygen, i.e. air-equilibrated solution).

This value is in good agreement with the singlet oxygen generation quantum yield, meaning that the quenching of the $^2E/^2T_1$ excited state mostly occurs by energy transfer.

From the Stern-Volmer equation:

$$\frac{\tau_0}{\tau} = 1 + K_{SV}[Q] = 1 + k_q\tau_0[Q] \qquad \text{(S11)}$$

by substituting the concentration of oxygen to [Q], it is also possible to estimate the Stern-Volmer (K_{SV}) and the quenching constants (k_q), which are, respectively:

$$K_{SV} = 1{,}050\ M^{-1}$$

$$k_q = 5.3 \times 10^7 M^{-1} s^{-1}$$

9 Stern-Volmer experiments and Rehm-Weller analysis

Stern-Volmer excited-state quenching experiments were performed by sequential addition of a concentrated solution of quencher in deoxygenated (Ar flushed) acetonitrile to a deoxygenated MeCN solution of (nBu$_4$N)[Cr(^{tBuPh}BTP)$_2$]. Absorption spectra were performed before and after the flushing and every addition. Stern-Volmer fittings based on emission intensities followed a similar equation to Eq. S11; however, instead of using the lifetimes (τ_0/τ), the ratio between the integrated emission spectra in the absence (I_0) and in presence (I) of increasing amount of quencher was used.[33] Since the detector used to collect the emission was not sensitive above 900 nm, emission spectra were initially recorded in the 750–900 nm range. The full spectrum (up to 1200 nm) was then acquired separately using an InGaAs detector. For the Stern–Volmer experiments, the spectra were reconstructed by aligning the two datasets at a common wavelength (either 860 or 875 nm). Emission spectra were corrected by the dilution factor. To validate the reliability of both approaches, we also performed a Stern–Volmer experiment for DIPEA by monitoring the change in lifetime, obtaining a comparable K_{SV} value of (0.35 ± 0.06) M^{-1}, in agreement with the value of (0.44 ± 0.01) M^{-1} from photoluminescence intensity measurements.

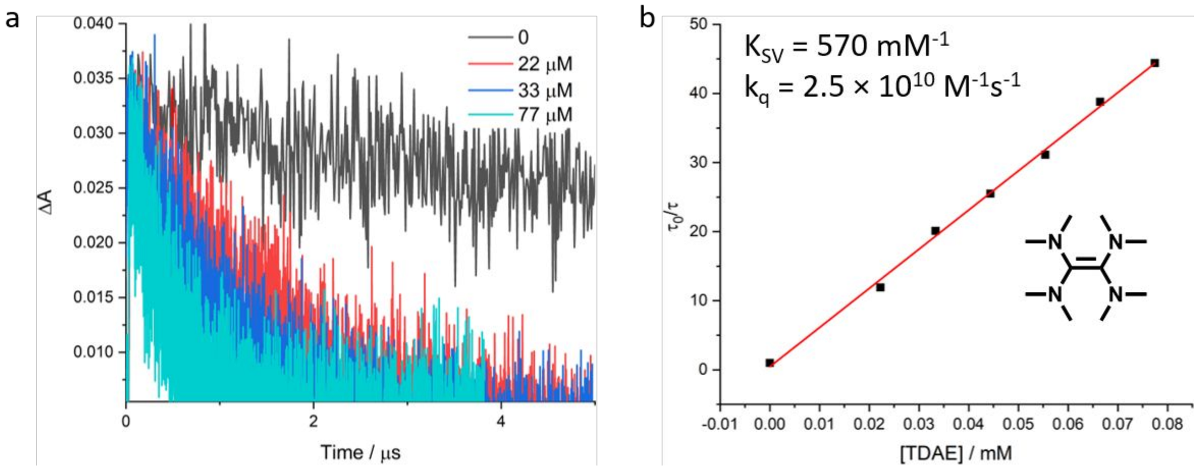

Figure S25. (a) Excited state absorption decays (λ_{ex} = 532 nm, 38 mJ, 200 ns of integration time, detected at 680 nm) of (nBu$_4$N)[Cr(^{tBuPh}BTP)$_2$] in deoxygenated MeCN upon addition of increasing amounts of TDAE (tetrakis(dimethylamino)ethylene) as electron donor and (b) obtained Stern-Volmer plot.

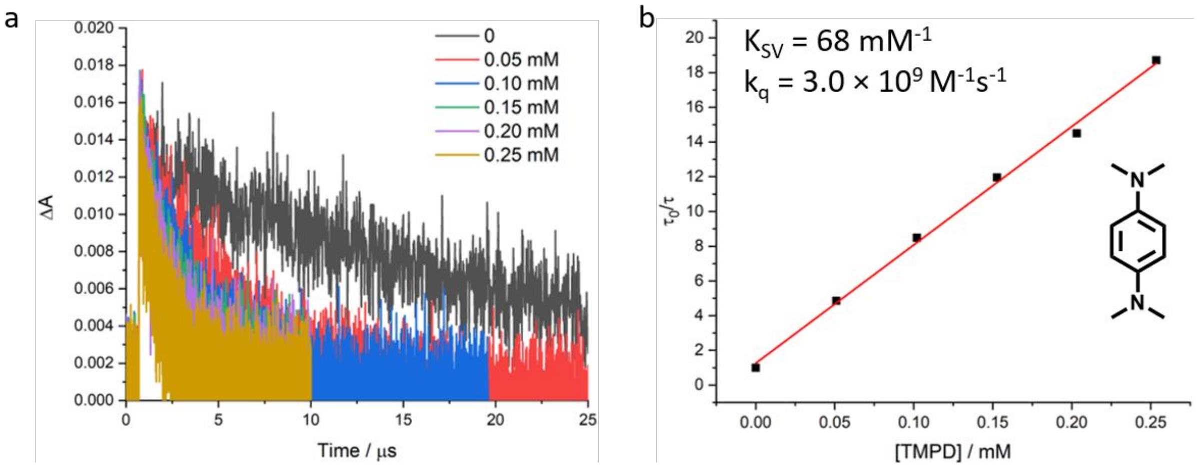

Figure S26. (a) Excited state absorption decays (λ_{ex} = 532 nm, 38 mJ, 200 ns of integration time, detected at 680 nm) of (nBu$_4$N)[Cr(^{tBuPh}BTP)$_2$] in deoxygenated MeCN upon addition of increasing amounts of TMPD (N^1,N^1,N^4,N^4-tetramethylbenzene-1,4-diamine) as electron donor and (b) obtained Stern-Volmer plot.

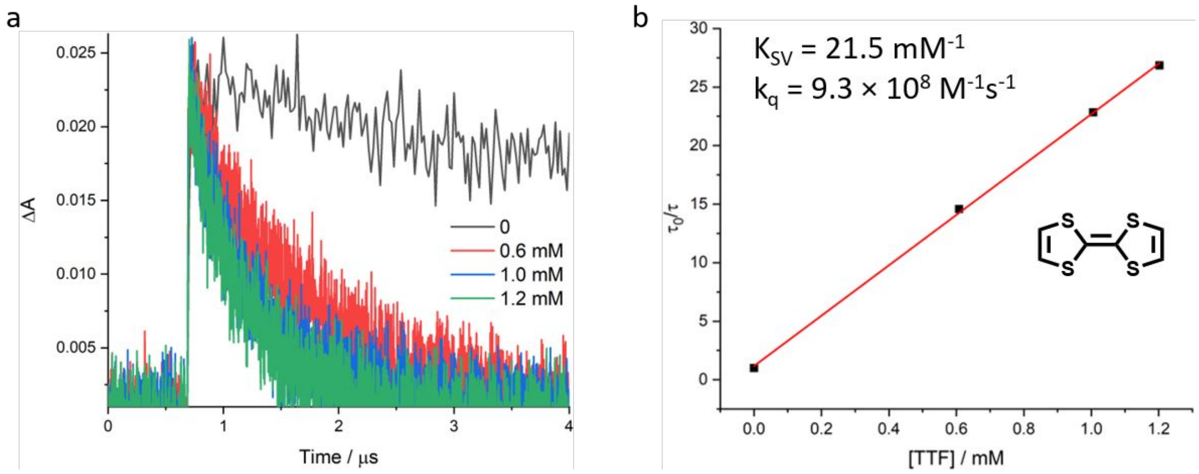

Figure S27. (a) Excited state absorption decays (λ_{ex} = 532 nm, 38 mJ, 200 ns of integration time, detected at 680 nm) of (nBu$_4$N)[Cr(^{tBuPh}BTP)$_2$] in deoxygenated MeCN upon addition of increasing amounts of TTF (2,2'-bi(1,3-dithiolylidene)) as electron donor and (b) obtained Stern-Volmer plot.

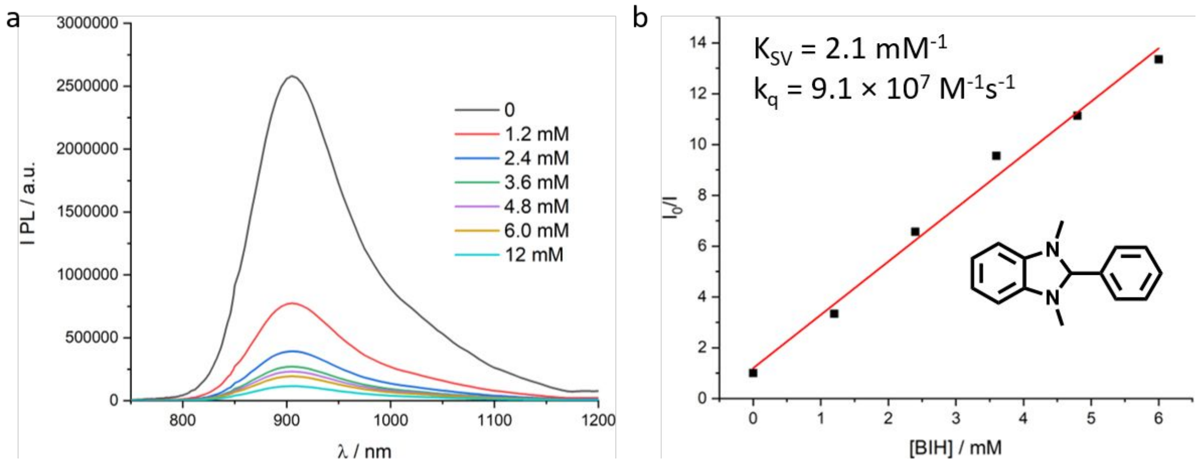

Figure S28. (a) Emission spectra (λ_{ex} = 475 nm, 2 summed spectra per scan, the entire spectra were obtained by recording the emission in the range 750-900 nm and reconstructed by matching the intensity at 860 nm of the full NIR spectrum recorded separately) of (nBu$_4$N)[Cr(^{tBuPh}BTP)$_2$] in deoxygenated MeCN upon addition of increasing amounts of BIH (1,3-dimethyl-2-phenyl-2,3-dihydro-1H-benzo[d]imidazole) as electron donor and (b) obtained Stern-Volmer plot.

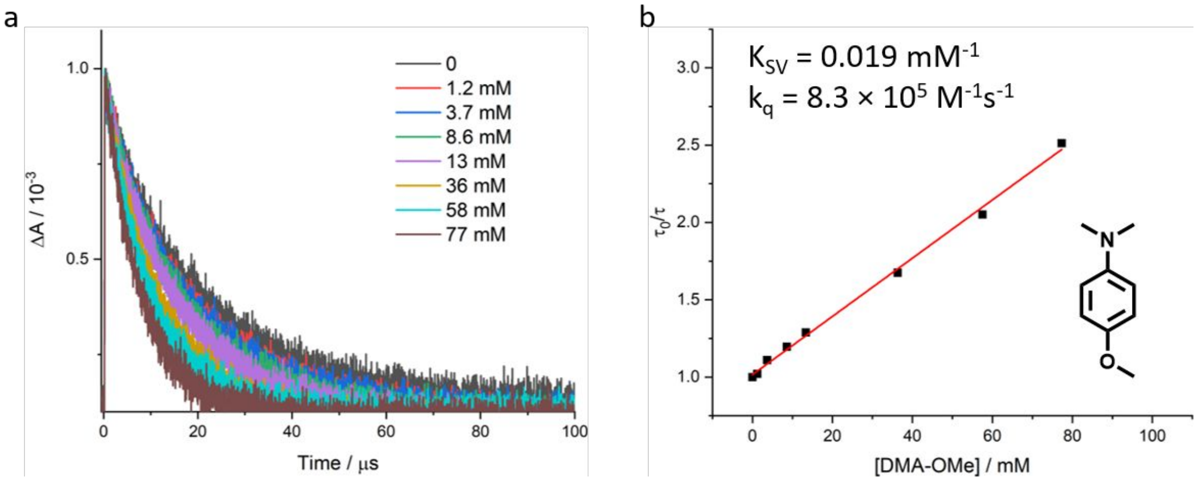

Figure S29. (a) Excited state absorption decays (λ_{ex} = 440 nm, 13 mJ, 200 ns of integration time, detected at 680 nm) of (nBu$_4$N)[Cr(^{tBuPh}BTP)$_2$] in deoxygenated MeCN upon addition of increasing amounts of DMA-OMe (4-methoxy-N,N-dimethylaniline) as electron donor and (b) obtained Stern-Volmer plot.

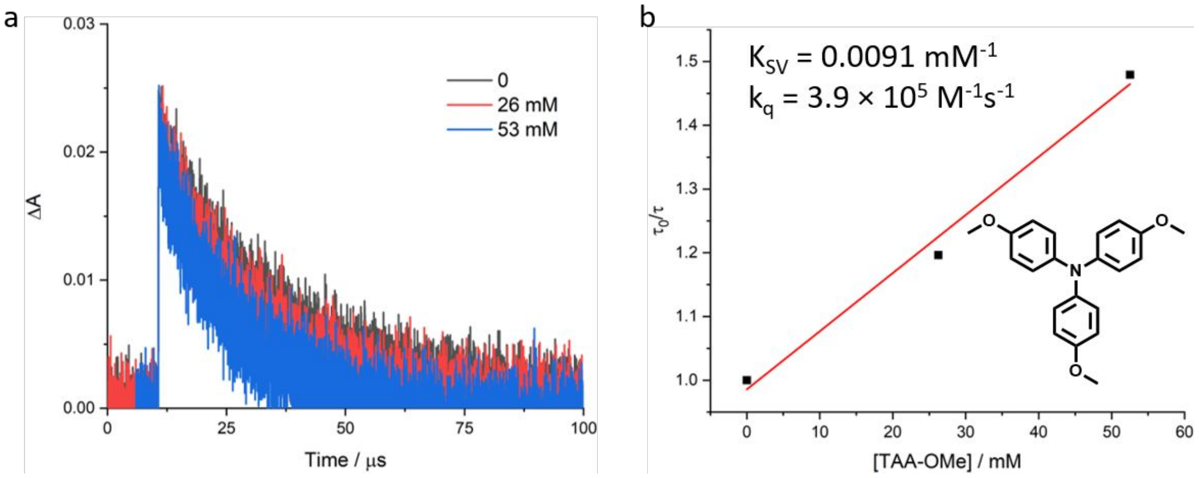

Figure S30. (a) Excited state absorption decays (λ_{ex} = 532 nm, 38 mJ, 200 ns of integration time, detected at 680 nm) of (nBu$_4$N)[Cr(^{tBuPh}BTP)$_2$] in deoxygenated MeCN upon addition of increasing amounts of TAA-OMe (tris(4-methoxyphenyl)amine) as electron donor and (b) obtained Stern-Volmer plot.

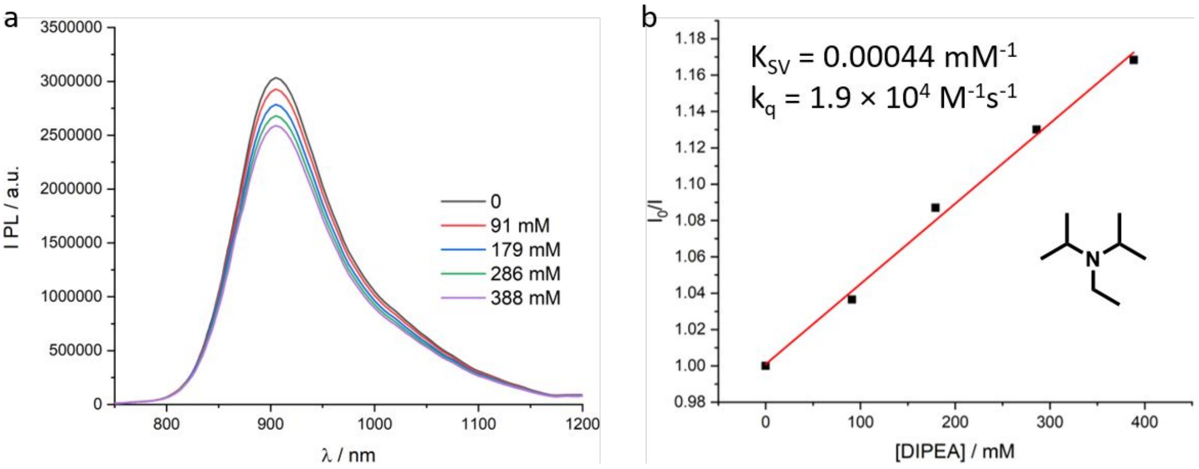

Figure S31. (a) Emission spectra (λ_{ex} = 475 nm, 2 summed spectra per scan, corrected by the dilution factor; the entire spectra are obtained by recording the emission in the range 750-900 nm and reconstructed by matching the intensity at 875 nm of the full NIR spectrum recorded separately) of (nBu$_4$N)[Cr(^{tBuPh}BTP)$_2$] in deoxygenated MeCN upon addition of increasing amounts of DIPEA (diisopropylamine) as electron donor and (b) obtained Stern-Volmer plot.

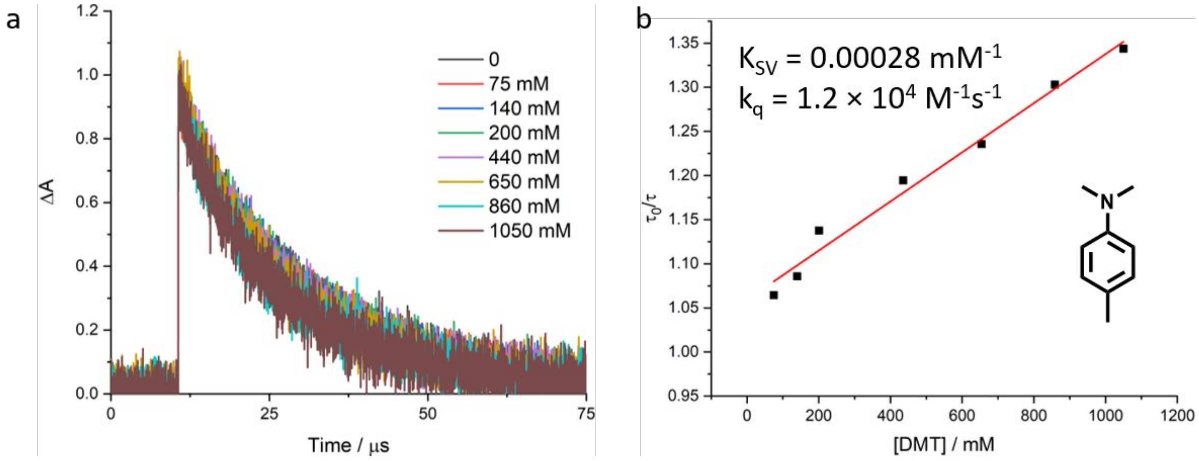

Figure S32. (a) Excited state absorption decays (λ_{ex} = 440 nm, 13 mJ, 200 ns of integration time, detected at 680 nm) of (nBu$_4$N)[Cr(^{tBuPh}BTP)$_2$] in deoxygenated MeCN upon addition of increasing amounts of DMT (N,N,4-trimethylaniline) as electron donor and (b) obtained Stern-Volmer plot.

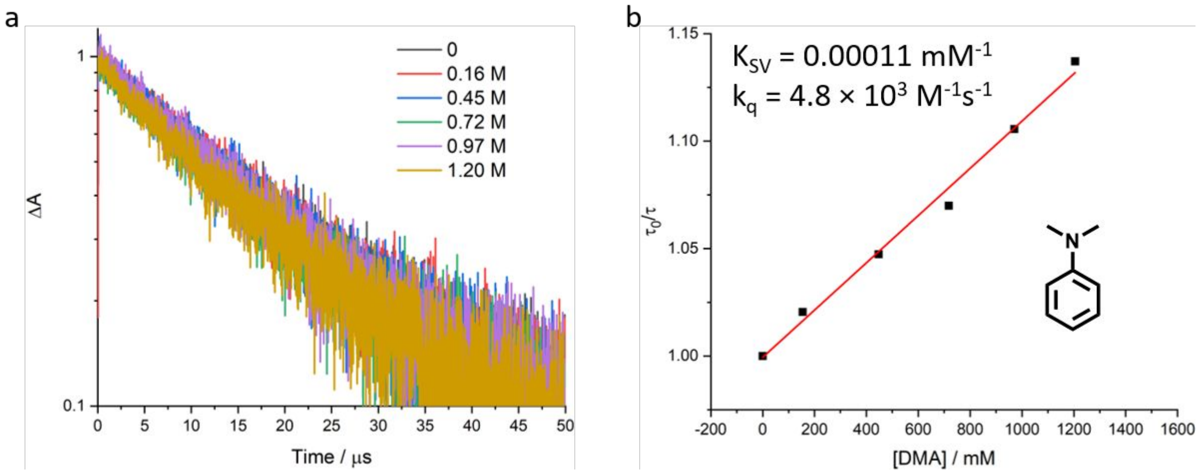

Figure S33. (a) Excited state absorption decays (λ_{ex} = 440 nm, 13 mJ, 200 ns of integration time, detected at 680 nm, logarithmic scale) of (nBu$_4$N)[Cr(^{tBuPh}BTP)$_2$] in deoxygenated MeCN upon addition of increasing amounts of DMA (N,N-dimethylaniline) as electron donor and (b) obtained Stern-Volmer plot.

Table S10. Oxidation potentials of the investigated electron donors in MeCN ($E_{ED+/ED}$, values found in literature), ΔG_{ET} of the energy transfer reaction with $^2E/^2T_1$ excited (nBu$_4$N)[Cr(^{tBuPh}BTP)$_2$] and resulting quenching constants (k_q) from the Stern-Volmer experiments.

**Electron donor**	**$E_{ED+/ED}$ vs SCE**	**ΔG_{ET} / eV [h]**	**k_q / $M^{-1}s^{-1}$ [i]**
TDAE	-0.61 [a]	–0.90	2.5×10^{10}
TMPD	+0.09 [b]	-0.20	3.0×10^{9}
TTF	+0.29 [b]	0.00	9.3×10^{8}
BIH	+0.33 [c]	+0.04	9.1×10^{7}
DMA-OMe	+0.55 [d]	+0.26	8.3×10^{5}
TAA-OMe	+0.58 [e]	+0.29	3.9×10^{5}
DIPEA	+0.64 [f]	+0.35	1.9×10^{4} [j]
DMT	+0.72 [g]	+0.43	1.2×10^{4}
DMA	+0.81 [g]	+0.52	4.8×10^{3}

[a] Based on ref [34]; [b] from ref [35]; [c] from ref [36]; [d] from ref [37]; [e] from ref [38]; [f] from ref [39]; [g] from ref [30]; [h] Calculated according to the formula: $\Delta G_{ET} = E_{ED+/ED} - (E_{*Cr-/Cr2-})$. The latter is the reduction potential of the complex in its $^2E/^2T_1$ excited state, i.e. +0.29 V vs SCE. The work terms are neglected for the outer-sphere reaction; [i] Determined using the Stern-Volmer equation (see Eq. S11); [j] The value obtained by monitoring the change in lifetime was 1.6×10^4 M^{-1}s^{-1}.

The obtained k_q values as a function of ΔG_{ET} were fitted with the empirical Rehm-Weller equation:[40–42]

$$k_q = \frac{k_d}{1+m\left[\exp\left(\frac{\Delta G^{\ddagger}_{ET}}{k_B T}\right)+\exp\left(\frac{\Delta G_{ET}}{k_B T}\right)\right]} \quad \text{(S12)}$$

Where k_d is the diffusion rate constant, k_B the Boltzmann constant and T the absolute temperature (298 K). The parameter m is an empirical factor that accounts for the rate at which the encounter complex (formed between the excited state of the complex and the electron donor) dissociates before electron transfer occurs. $\Delta G_{ET}^{\neq}$ is the activation energy, which can be expressed as:

$$\Delta G^{\ddagger}_{ET} = [(\frac{\Delta G_{ET}}{2})^2 + \Delta G^{\ddagger}_{ET}(0)^2]^{\frac{1}{2}} + (\frac{\Delta G_{ET}}{2}) \quad \text{(S13)}$$

Here $\Delta G_{ET}^{\neq}(0)$ represents the free energy of activation in the case where $\Delta G_{ET} = 0$.

The best fit (R^2 = 0.999) was obtained with the values of:

$k_d = (3.04 \pm 0.05) \times 10^{10}$ M^{-1}s^{-1}

$\Delta G^{\ddagger}_{ET}(0) = (0.230 \pm 0.003)$ eV

$m = 0.025$

The diffusion constant obtained from the fitting appears significantly higher than the value reported for acetonitrile (~2.5×10^{10} $M^{-1}s^{-1}$).[43] However, the quenching constant measured in the presence of TDAE is in good agreement with this diffusion limit. We attribute the discrepancy in the fitted diffusion constant to the limited number of experimental data points in the highly exergonic region (very negative ΔG), where the fitting is most sensitive to accurately estimating this parameter.

From $\Delta G_{ET}^{\ddagger}(0)$ it is possible to estimate the outer-sphere reorganization energy λ_o according to the relationship:

$$\lambda_o = 4 \times \Delta G^{\ddagger}_{ET}(0) = (0.92 \pm 0.02)\ \text{eV} \qquad \text{(S14)}$$

10 HPLC-MS calibration curve of α-bromoacetophenone and acetophenone

Solutions at four different concentrations (0.1 mM, 0.5 mM, 1.0 mM and 2.0 mM) of α-bromoacetophenone and acetophenone where prepared in 1.2 mL of HPLC-grade MeCN. 10 μL of each solution were injected in the HPLC column. The photodiode-array (PDA) signals were collected, showing one peak at the elution time of 4.8 and 4.2 minutes for α-bromoacetophenone and acetophenone, respectively. The chromatograms were analyzed with MestReNova. Correlation between the area of the peaks and the concentration yielded the calibration curve for both compounds.

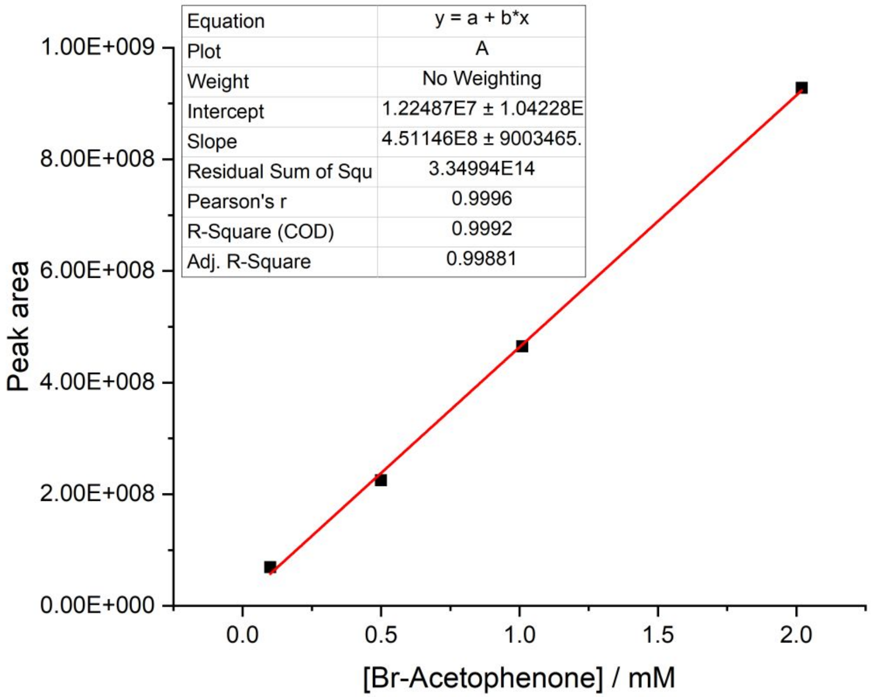

Figure S34. Calibration curve for α-bromoacetophenone.

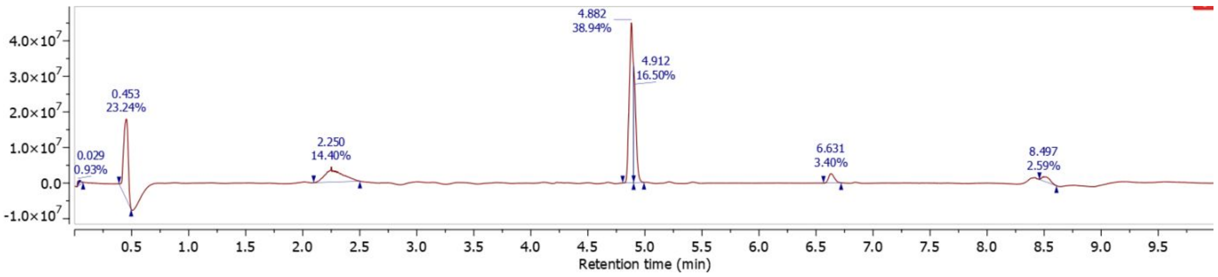

Figure S35. HPLC-PDA Chromatogram of a 1 mM solution of α-bromoacetophenone in MeCN.

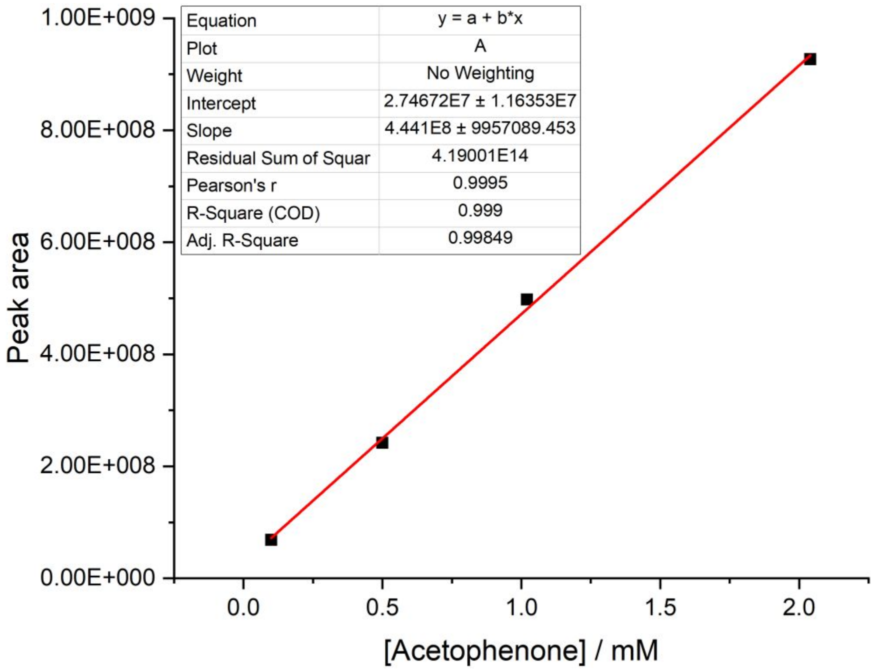

Figure S36. Calibration curve for acetophenone.

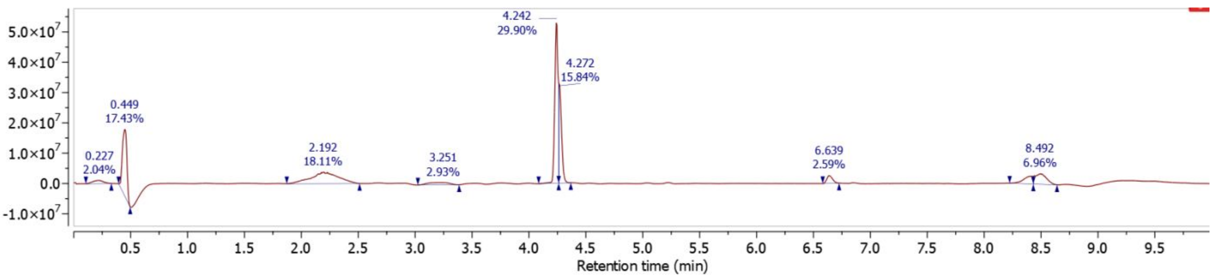

Figure S37. HPLC-PDA Chromatogram of a 1 mM solution of acetophenone in MeCN.

11 Photocatalytic experiments

11.1 Dehalogenation of α-bromoacetophenone

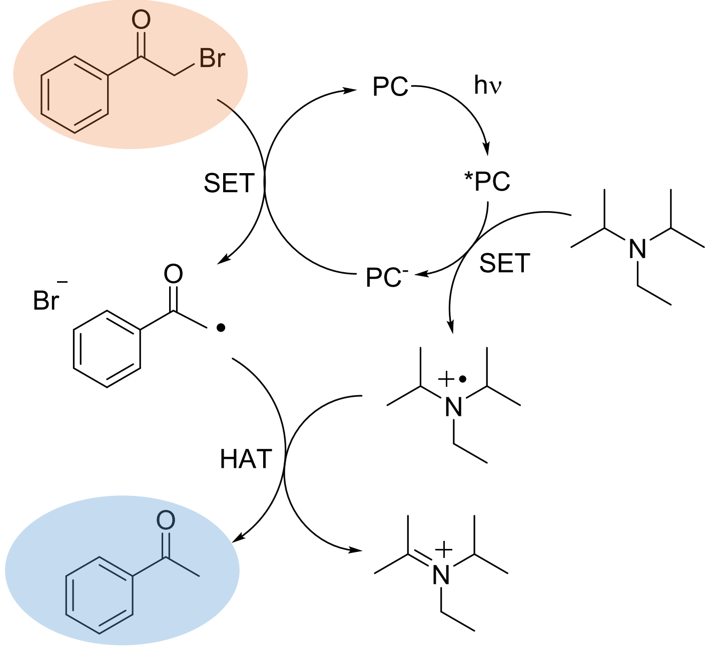

Figure S38. Plausible mechanism for the dehalogenation of α-bromoacetophenone in the presence of $[Cr(^{tBuPh}BTP)_2]^-$ as photocatalyst (PC in the figure) and DIPEA as electron donor.[38,44] Here, hν indicates the photoexcitation, SET stands for single electron transfer and HAT stands for hydrogen atom transfer.

Dehalogenation of bromoacetophenone at 405 nm. 40 mg of α-bromoacetophenone (0.2 mmol), 350 μL of DIPEA (2 mmol), 5 mg of $(nBu_4N)[Cr(^{tBuPh}BTP)_2]$ (0.004 mmol) where dissolved in 2.5 mL of anhydrous acetonitrile in a flame-dried 5-mL Schlenk flask and degassed by flushing argon for 10 minutes. The flask was dipped in a beaker full of distilled water and irradiated while stirring at a distance of 5 cm from a 405-nm LED, where the sample was homogeneously irradiated with a power of 250 mW.

In this case, the energy per photon is 4.90×10^{-19} J, which implies a flux of 0.85 μE/s, or 3 mE/h.

The expected absorbance of the photocatalyst was superior to 4 (quantitative absorption of the incident photons), while the absorbance of the bromoacetophenone was computed to be 0.08. The quenching efficiency of DIPEA at the concentration used in our experiments was estimated to be around 24% according to the Stern-Volmer equation (see Eq. S10 and Eq. S11). α-bromoacetophenone alone is not capable of quenching the excited state of the Cr^{III} complex. 50 μL of solution were withdrawn before the irradiation and at specific times of irradiation, diluted in 1 mL of MeCN and analyzed by HPLC. The concentration inside of the HPLC vial was determined by using calibration curves and monitoring the peaks at 4.8 min (for the reagent) and 4.2 min (for the product). The conversion in percentage at the irradiation time *t* was determined with the following equation:

$$Conv(t) = 100\% \times (1 - \frac{[Br-acetophenone]_t}{[Br-acetophenone]_0}) \qquad (S15)$$

Where $[Br\text{-}acetophenone]_t$ is the concentration of the reagent at time *t*, while $[Br\text{-}acetophenone]_0$ is its initial concentration.

The yield in percent was calculated with the following formula:

$$Yield(t) = 100\% \times (\frac{[Acetophenone]_t}{[Br-acetophenone]_0}) \qquad (S16)$$

Where $[Acetophenone]_t$ is the concentration of the product at time *t*.

Two control experiments were performed (a) in the absence of (nBu$_4$N)[Cr(^{tBuPh}BTP)$_2$] and (b) in absence of both (nBu$_4$N)[Cr(^{tBuPh}BTP)$_2$] and DIPEA.

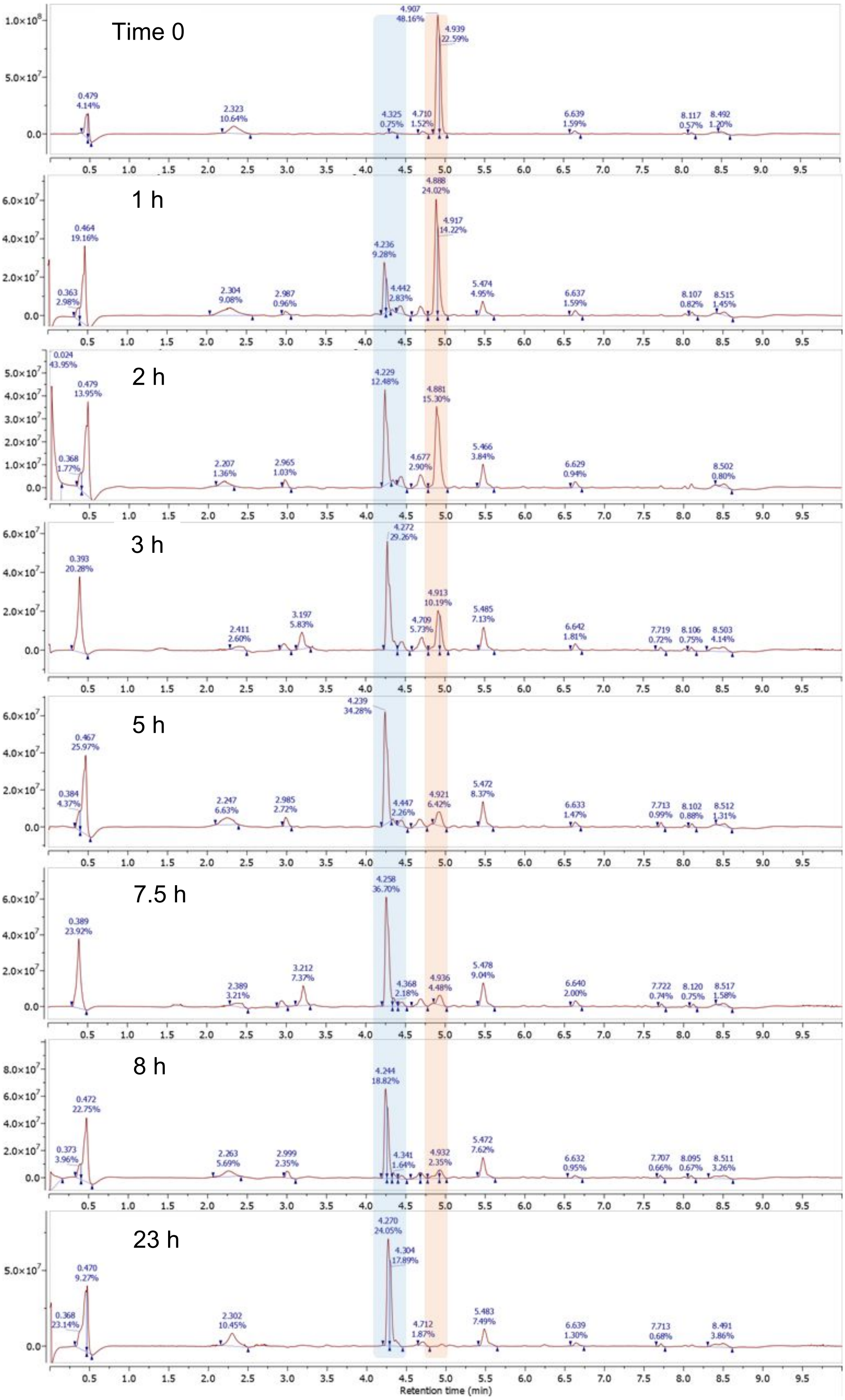

Figure S39. PDA chromatograms of the photocatalytic debromination of α-bromoacetophenone upon irradiation at 405 nm in the presence of 2 mol% of (nBu$_4$N)[Cr(^{tBuPh}BTP)$_2$].

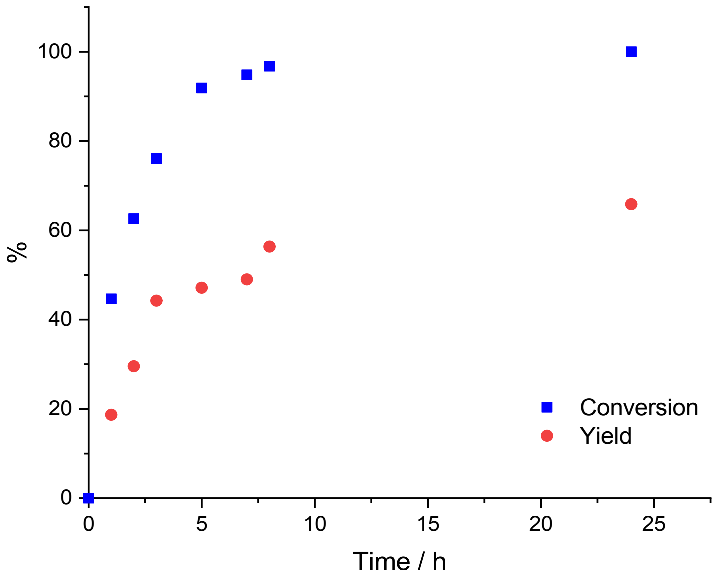

Figure S40. Plot of the conversion and yield as a function of time for the photocatalytic debromination of α-bromoacetophenone upon irradiation at 405 nm in the presence of 2 mol% of (nBu$_4$N)[Cr(^{tBuPh}BTP)$_2$].

The photoreaction quantum yield in the presence of the Cr^{III} photocatalyst during the first 3 h, where there is still linearity between product concentration and reaction time, was determined by dividing the number of mmols of product by the mE of absorbed photons according to the formula:

$$\Phi_R = \frac{mmol_{product}}{mE_{absorbed}} = \frac{(44\%)(0.20\ mmol)}{(3\ \frac{mE}{h})(3\ h)} \sim 1\% \qquad \text{(S17)}$$

Where 44% is the reaction yield after 3 h. The LED emission spectrum spans a range from roughly 375 to 430 nm; for simplicity, the LED output was treated here as monochromatic 405 nm light. This approximation allows an order of magnitude estimation of the reaction quantum yield.

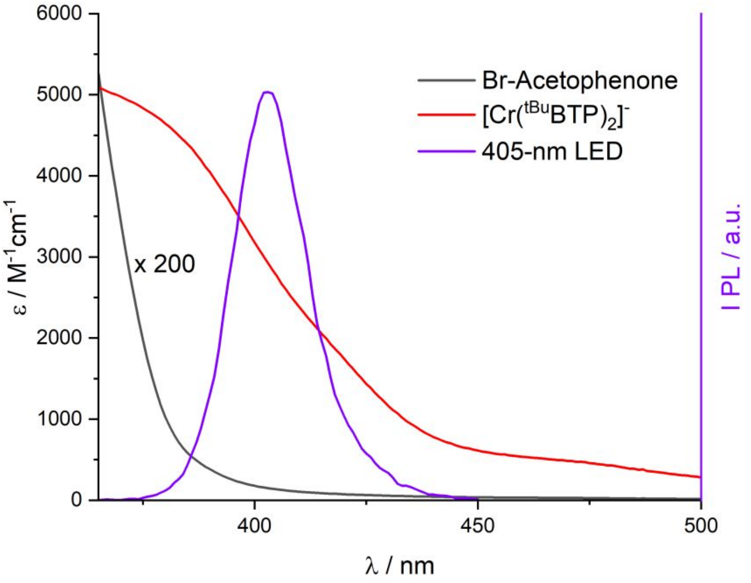

Figure S41. Comparison between the molar absorption coefficients of [Cr(^{tBuPh}BTP)$_2$]$^-$ (red line), α-bromoacetophenone (dark grey line, magnified spectrum, multiplied by a factor 200) in acetonitrile, overlapped with the emission spectrum of the 405-nm LED (violet line) used for the irradiation.

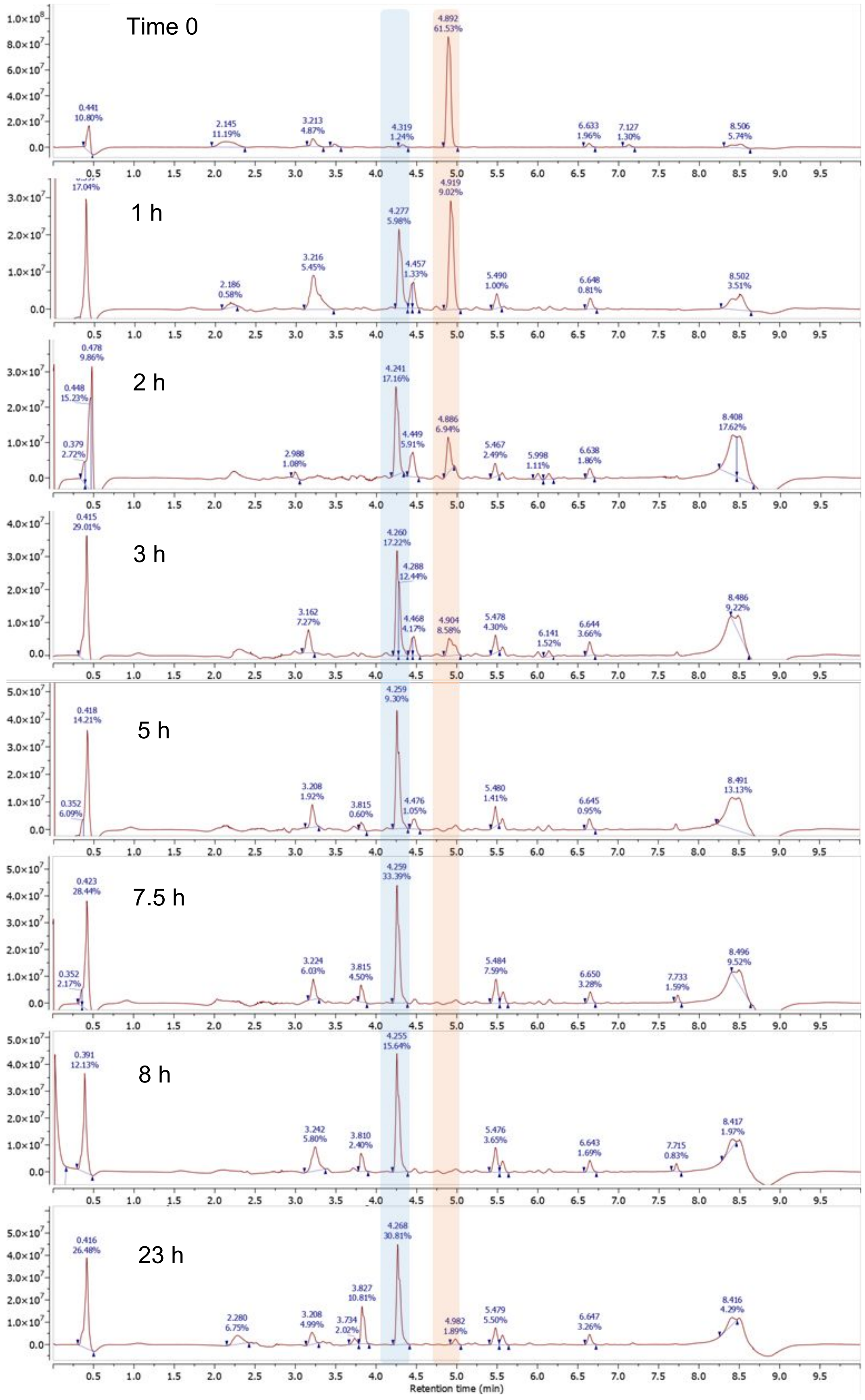

Figure S42. PDA chromatograms of the photocatalytic debromination of α-bromoacetophenone upon irradiation at 405 nm in absence of (nBu$_4$N)[Cr(^{tBuPh}BTP)$_2$].

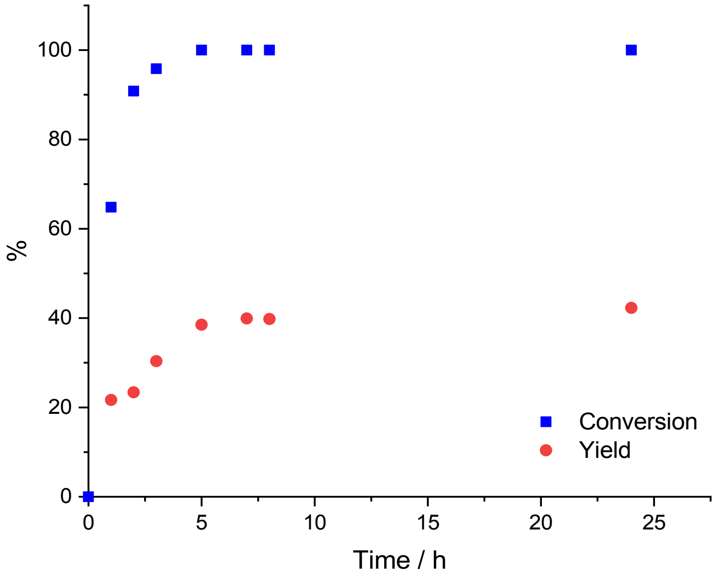

Figure S43. Plot of the conversion and yield as a function of time for the photocatalytic debromination of α-bromoacetophenone upon irradiation at 405 nm in absence of (nBu$_4$N)[Cr(^{tBuPh}BTP)$_2$].

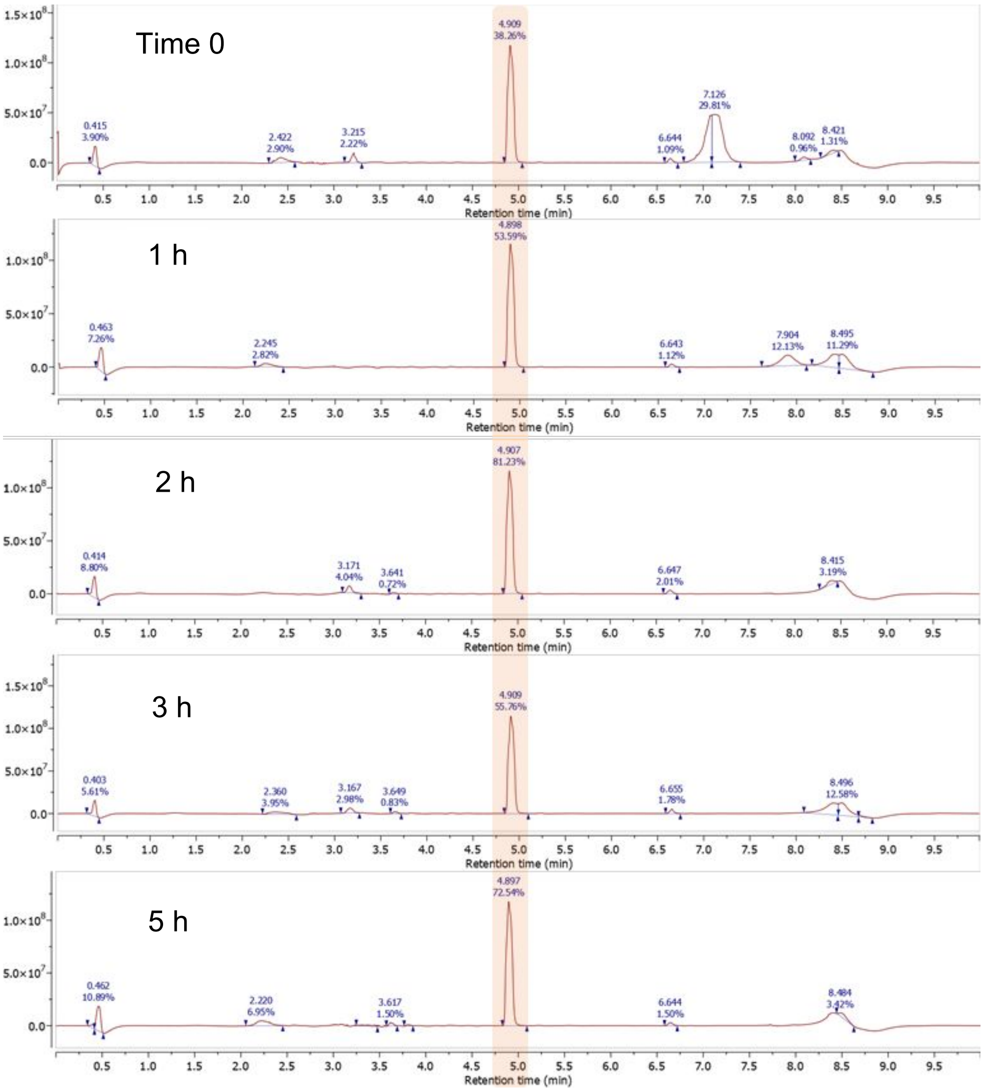

Figure S44. PDA chromatograms of the photocatalytic debromination of α-bromoacetophenone upon irradiation at 405 nm in the absence of both (nBu$_4$N)[Cr(^{tBuPh}BTP)$_2$] and DIPEA.

Table S11. Conversion and yield at different irradiation times for the photocatalytic debromination of α-bromoacetophenone upon irradiation at 405 nm in the presence (second and third column) and in absence (fourth and fifth column) of (nBu$_4$N)[Cr(^{tBuPh}BTP)$_2$] and in absence of DIPEA (sixth and seventh column).

	**With Photocatalyst**		**Control with DIPEA**		**Control without DIPEA**	
**Time / h**	**Conversion[a] / %**	**Yield[a] / %**	**Conversion[a] / %**	**Yield[a] / %**	**Conversion[a] / %**	**Yield[a] / %**
1	45	19	65	22	0	0
2	63	30	91	23	0	0
3	76	44	96	30	0	0
5	92	47	>99	39	0	0
7	95	49	>99	40	-	-
8	97	56	>99	40	-	-
24	>99	66	>99	42	-	-

[a] The expected error in the percentage value is ±1.

Dehalogenation of α-bromoacetophenone at 632 nm. 40 mg of α-bromoacetophenone (0.2 mmol), 350 μL of DIPEA (2 mmol), 5 mg of (nBu$_4$N)[Cr(^{tBuPh}BTP)$_2$] (0.004 mmol) were dissolved in 2.5 mL of anhydrous acetonitrile in a flame-dried 5-mL Schlenk flask and degassed by flushing argon for 10 minutes. The flask was dipped in a beaker full of distilled water and irradiated while stirring at a distance of 5 cm from a 632-nm LED.

At that distance, the sample was homogeneously irradiated with a power of 2 W. Since the average energy per photon is 3.14×10^{-19} J, this results in a flux of incident photons of 10.6 μE/s, or 38 mE/h.

The estimated absorbance of the sample (considering the concentration, i.e., 1.6 mM, the molar absorption coefficient at 632 nm, i.e., 13 $M^{-1}cm^{-1}$, and the average optical path of the Schlenk flask, i.e. 1.5 cm) is 0.03. Under these conditions, 7% of the incident light are absorbed. Neither α-bromoacetophenone nor acetophenone absorb substantially at 632 nm (Fig. S47).

The quenching efficiency of DIPEA at the concentration used in our experiments was estimated to be around 24% according to the Stern-Volmer equation (see Eq. S10 and S11). 50 μL of solution were withdrawn before the irradiation and at specific times of irradiation, diluted in 1 mL of MeCN and analyzed by HPLC. The concentration inside of the HPLC vial was determined by using calibration curves and monitoring the peaks at 4.8 min (for the reagent) and 4.2 min (for the product). The conversion in percentage at the irradiation time t was determined with Eq. S15 and Eq. S16, respectively.

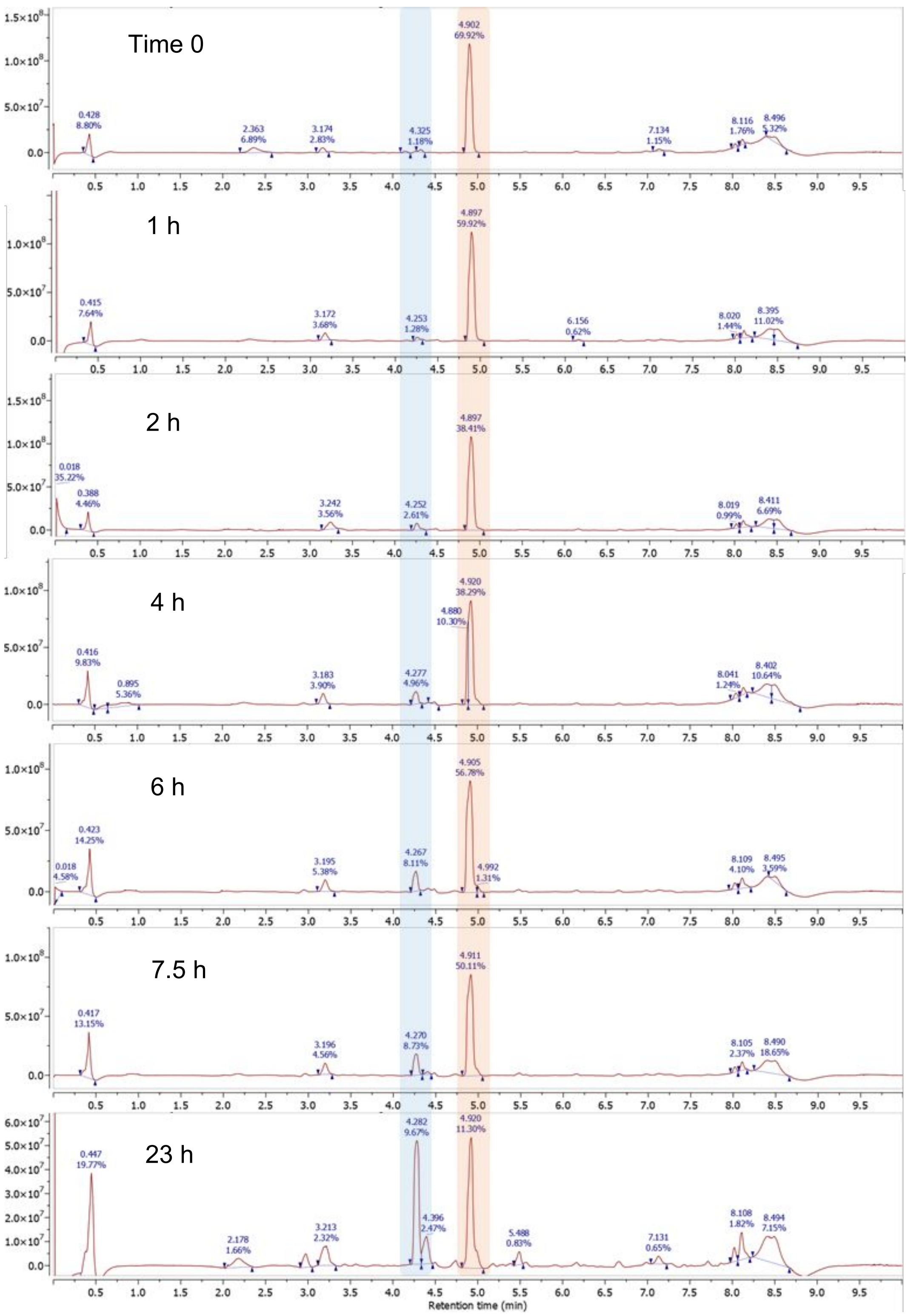

Figure S45. PDA chromatograms of the photocatalytic debromination of α-bromoacetophenone upon irradiation at 632 nm in the presence of 2 mol% of (nBu$_4$N)[Cr(^{tBuPh}BTP)$_2$].

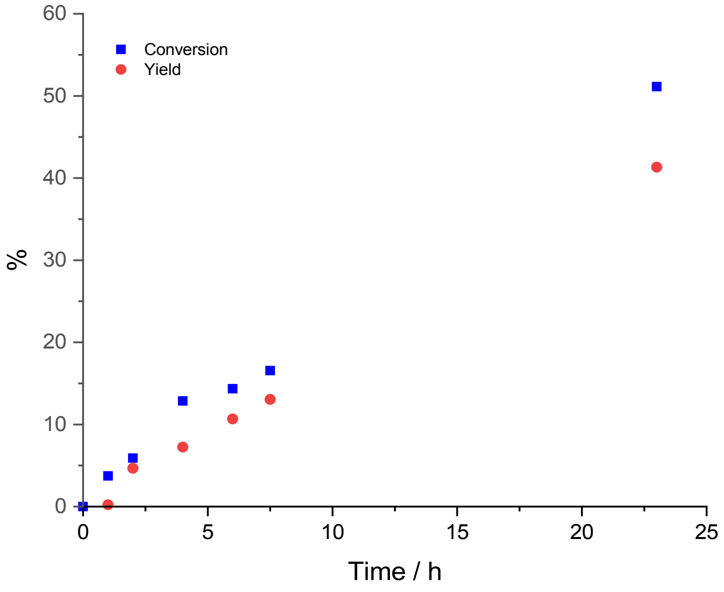

Figure S46. Plot of the conversion and yield as a function of time for the photocatalytic debromination of α-bromoacetophenone upon irradiation at 632 nm in the presence of 2 mol% of (nBu$_4$N)[Cr(^{tBuPh}BTP)$_2$].

The photoreaction quantum yield after 23 h was determined by dividing the number of mmols of product by the mE of absorbed photons according to Eq. S17:

$$\Phi_R = \frac{mmol_{product}}{mE_{absorbed}} = \frac{(41\%)(0.20\ mmol)}{(7\%)(38\ \frac{mE}{h})(23\ h)} = 0.13\%$$

Where 41% is the reaction yield and 7% is the fraction of absorbed photons.

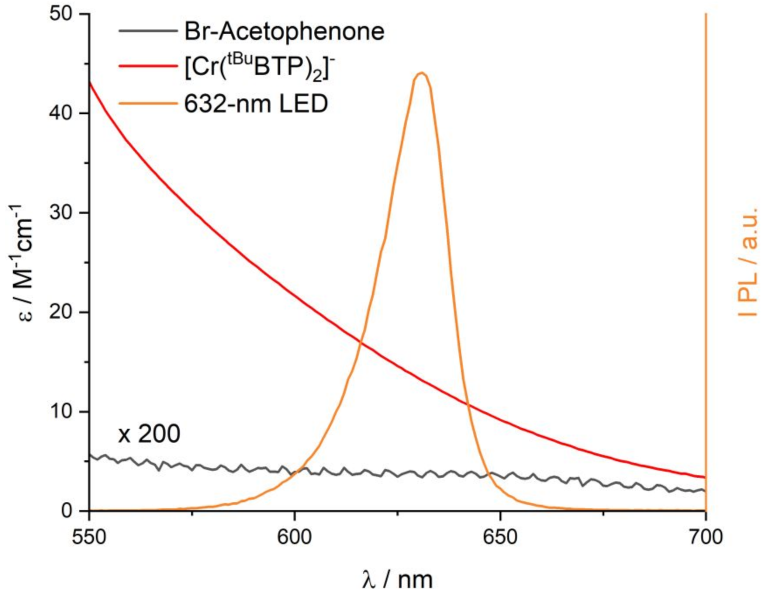

Figure S47. Comparison between the molar absorption coefficients of [Cr(^{tBuPh}BTP)$_2$]$^-$ (red line), α-bromoacetophenone (dark grey line, magnified spectrum, multiplied by a factor 200) in acetonitrile, overlapped with the emission spectrum of the 632-nm LED (orange line) used for the irradiation.

The control experiment was performed and checked in the same way as the one reported above, but no (nBu$_4$N)[Cr(^{tBuPh}BTP)$_2$] was added.

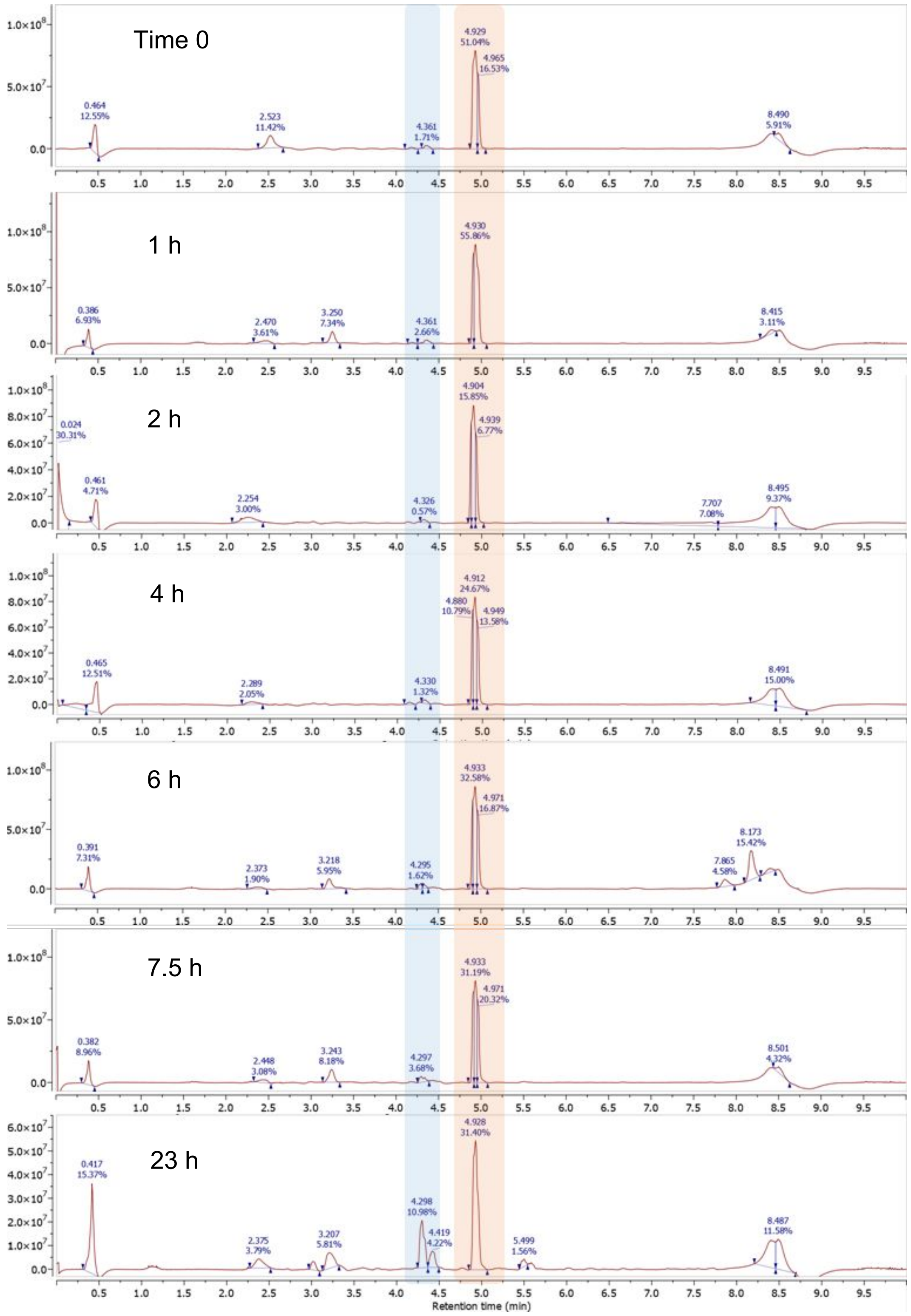

Figure S48. PDA chromatograms of the photocatalytic debromination of α-bromoacetophenone upon irradiation at 632 nm in the absence of (nBu$_4$N)[Cr(^{tBuPh}BTP)$_2$].

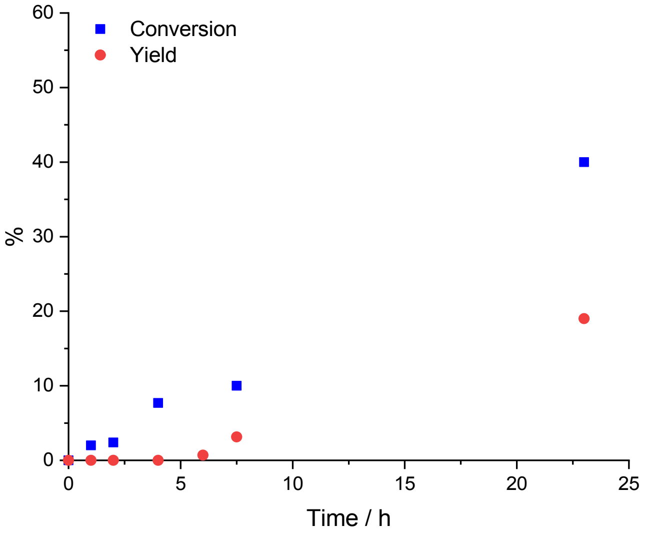

Figure S49. Plot of the conversion and yield as a function of time for the photocatalytic debromination of α-bromoacetophenone upon irradiation at 632 nm in the absence of (nBu$_4$N)[Cr(^{tBuPh}BTP)$_2$].

Table S12. Conversion and yield at different irradiation times for the photocatalytic debromination of α-bromoacetophenone upon irradiation at 632 nm in the presence (second and third column) and in the absence (fourth and fifth column) of (nBu$_4$N)[Cr(^{tBuPh}BTP)$_2$].

	**With Photocatalyst**		**Control with DIPEA**	
**Time / h**	**Conversion[a] / %**	**Yield[a] / %**	**Conversion[a] / %**	**Yield[a] / %**
1	4	0	2	0
2	6	5	2	0
4	13	7	8	0
6	14	11	-[b]	1
7.5	17	13	10	3
23	51	41	40	19

[a] The expected error in the percentage value is ±1; [b] The integrated area of the reagent was higher than in the previous measurement, likely due to the presence of an intermediate or by-product with the same retention time.

The control experiment exhibited a yield of 0% for nearly 6 h, along with the progressive disappearance of the reagent. After 7.5 h, the product was detected despite the fact that the reagent α-bromoacetophenone does not significantly absorb at the irradiation wavelengths, as shown in Fig. S47. Although purely speculative, we tentatively attribute this behavior to the slow reaction between α-bromoacetophenone and DIPEA, leading to enolate formation. The resulting enolate, having a more extended π-conjugation, may potentially absorb at longer wavelengths and could thus contribute to the observed debromination.

11.2 Oxidation of α-terpinene with singlet oxygen

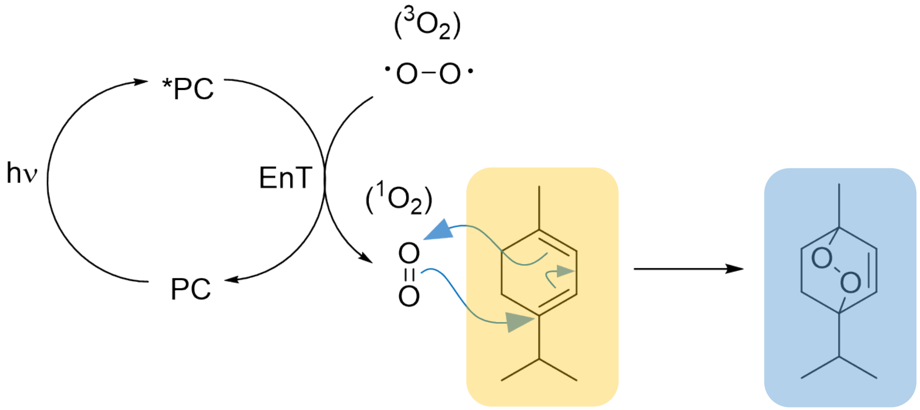

Figure S50. Plausible mechanism for the oxidation of α-terpinene initiated by Dexter energy transfer (EnT) from the excited state of $[Cr(^{tBuPh}BTP)_2]^-$ (PC) to dioxygen.[45]

Oxidation of α-terpinene under air-equilibrated conditions. 17.5 μL of α-terpinene (0.1 mmol), 2 mg of $(PPN)[Cr(^{tBuPh}BTP)_2]$ (0.001 mmol) and 2 mg of hexamethylcyclotrisiloxane as an internal standard (0.01 mmol) were dissolved in 1 mL of air-equilibrated CD_3CN. 0.5 mL of this solution were introduced in an NMR tube, which was dipped into a water bath (to avoid excess heating) and homogeneously irradiated at 632 nm at a distance of 5 cm (output power 3.6 W, 0.4 W reaching the sample). At specific times, ^1H-NMR experiments (CD_3CN, 250 MHz, 8 scans, 298 K) were performed.

A control sample was prepared and irradiated in the same way in the absence of photocatalyst.

The peaks that were following for monitoring of the reaction are: 5.59 ppm (reagent, vinylic protons), 6.47 ppm (product, vinylic protons). The integration was performed by comparison with the integral of the peak at 0.14 ppm (internal standard).

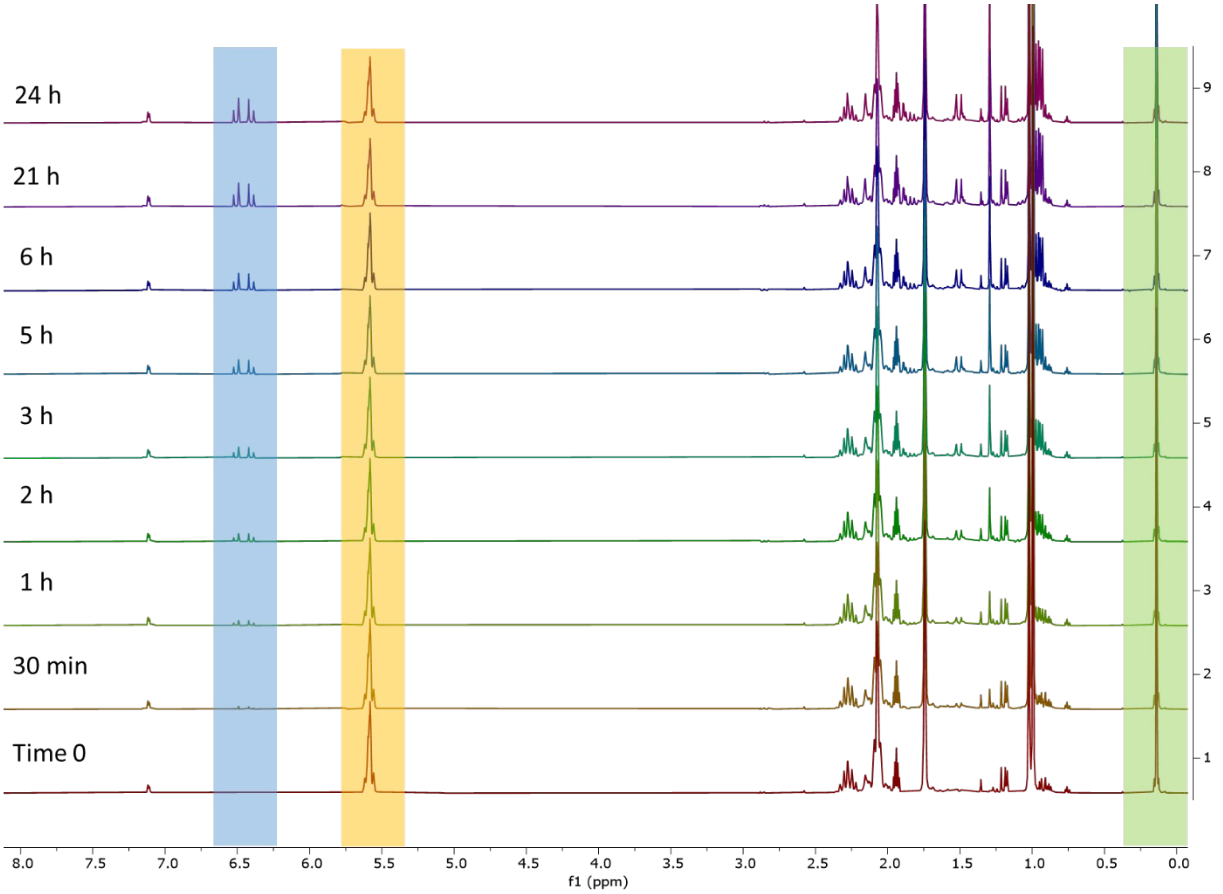

Figure S51. ^{1}H-NMR (CD_3CN, 250 MHz) spectra of a solution of 0.05 mmol α-terpinene, 1.0 mol% (PPN)[Cr(^{tBuPh}BTP)$_2$], 0.005 mmol of hexamethylcyclotrisiloxane as internal standard in 0.5 mL of air-equilibrated CD_3CN after the indicated irradiation times. The signals highlighted in blue are due to the endoperoxide product, the signal in yellow is due to the reagent and the signal of the internal standard is highlighted in green.

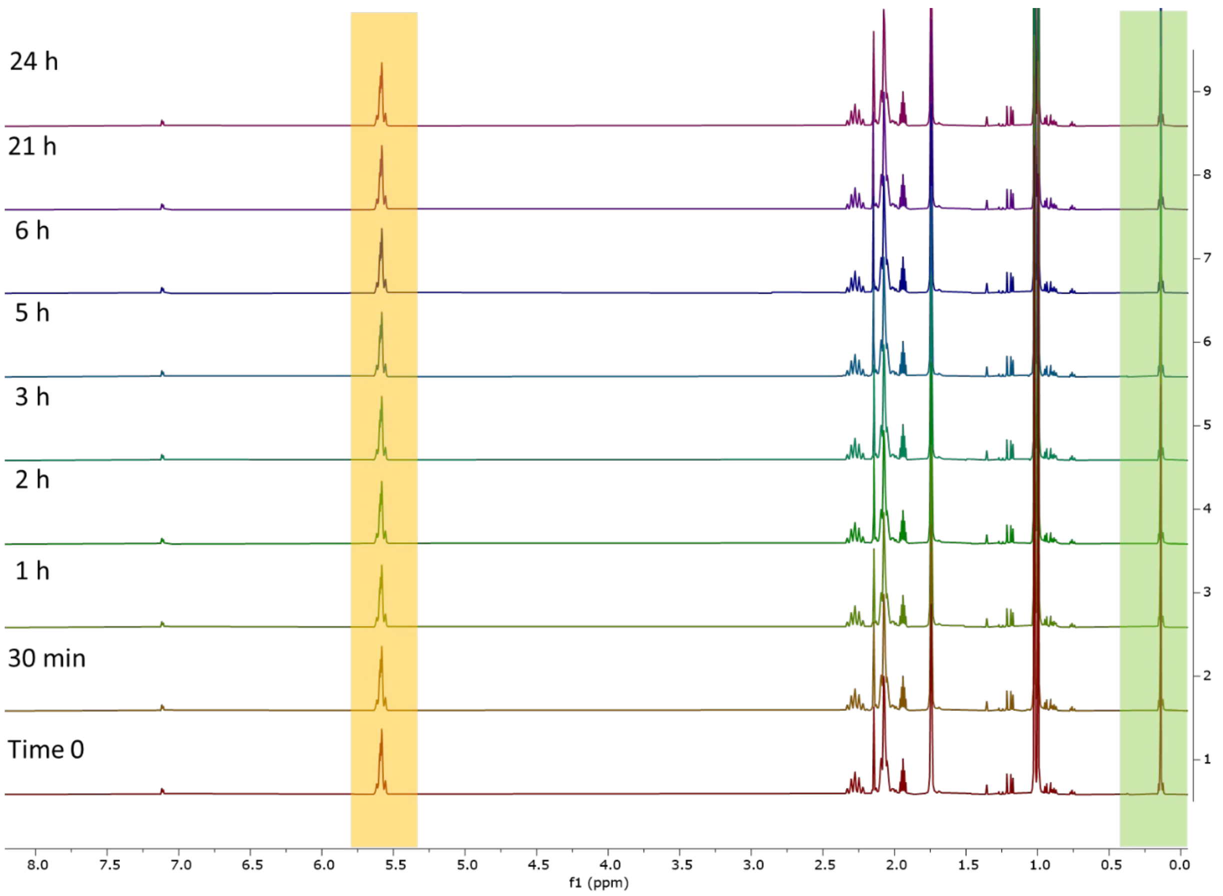

Figure S52. ^{1}H-NMR (CD_3CN, 250 MHz) spectra of a solution of 0.05 mmol α-terpinene and 0.005 mmol of hexamethylcyclotrisiloxane as internal standard in 0.5 mL of air-equilibrated CD_3CN after the indicated irradiation times. The signal highlighted in yellow is due to the reagent and the signal of the reference is highlighted in green.

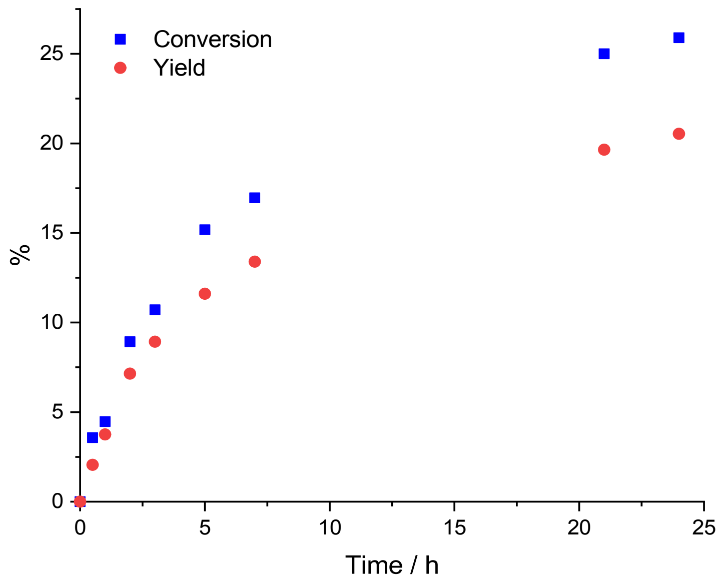

Figure S53. Plot of the conversion and yield as a function of time for the photocatalytic oxidation of α-terpinene upon irradiation at 632 nm in the presence of 1 mol% of (PPN)[Cr(^{tBuPh}BTP)$_2$] in air-equilibrated CD_3CN.

Table S13. Substrate conversion and yield determined by integration of the characteristic peaks in the ^{1}H-NMR spectra by using hexamethylcyclotrisiloxane as internal standard. The last two columns refer to the control experiment in the absence of photocatalyst.

	With photocatalyst		Control experiment	
**Time / h**	**Conversion[a] / %**	**Yield[a] / %**	**Conversion[a] / %**	**Yield[a] / %**
0.5	3.6	2.1	1.9	0
1	4.5	3.8	2.9	0
2	8.9	7.1	2.9	0
3	10.7	8.9	3.8	0
5	15.2	11.6	4.8	0
6	17.0	13.4	4.8	0
21	25.0	19.6	4.8	0
24	25.9	20.5	4.8	0

[a] The expected error in the percentage value is ±0.1.

Oxidation of α-terpinene under oxygen atmosphere. The samples were prepared as described above for the air-equilibrated measurements. However, prior to irradiation, both solutions (with and without photocatalyst) were purged with oxygen for 1 minute.

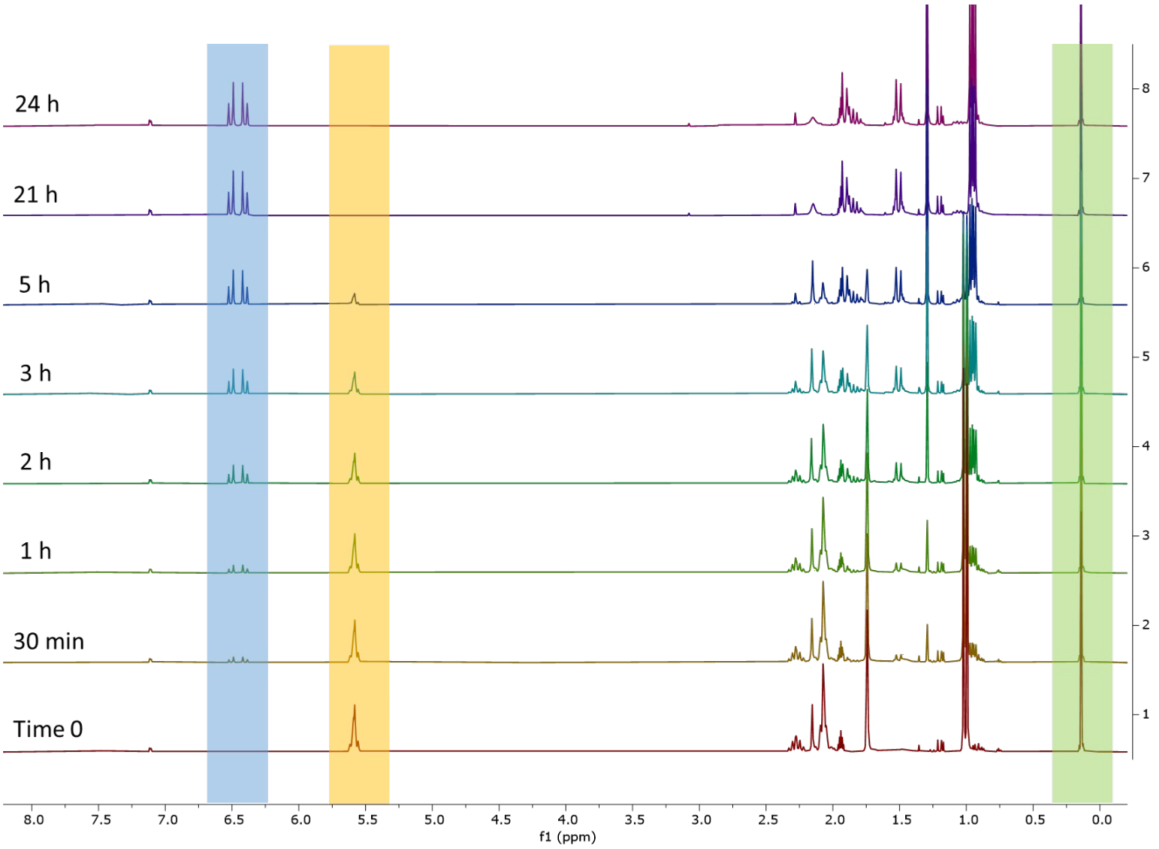

Figure S54. ^{1}H-NMR (CD_3CN, 250 MHz) spectra of a solution of 0.05 mmol α-terpinene, 1.0 mol% (PPN)[Cr(^{tBuPh}BTP)$_2$], 0.005 mmol of hexamethylcyclotrisiloxane as internal standard in 0.5 mL of oxygen-equilibrated CD_3CN after the indicated irradiation times. The signals highlighted in blue are due to the endoperoxide product, the signal in yellow is due to the reagent and the signal of the internal standard is highlighted in green.

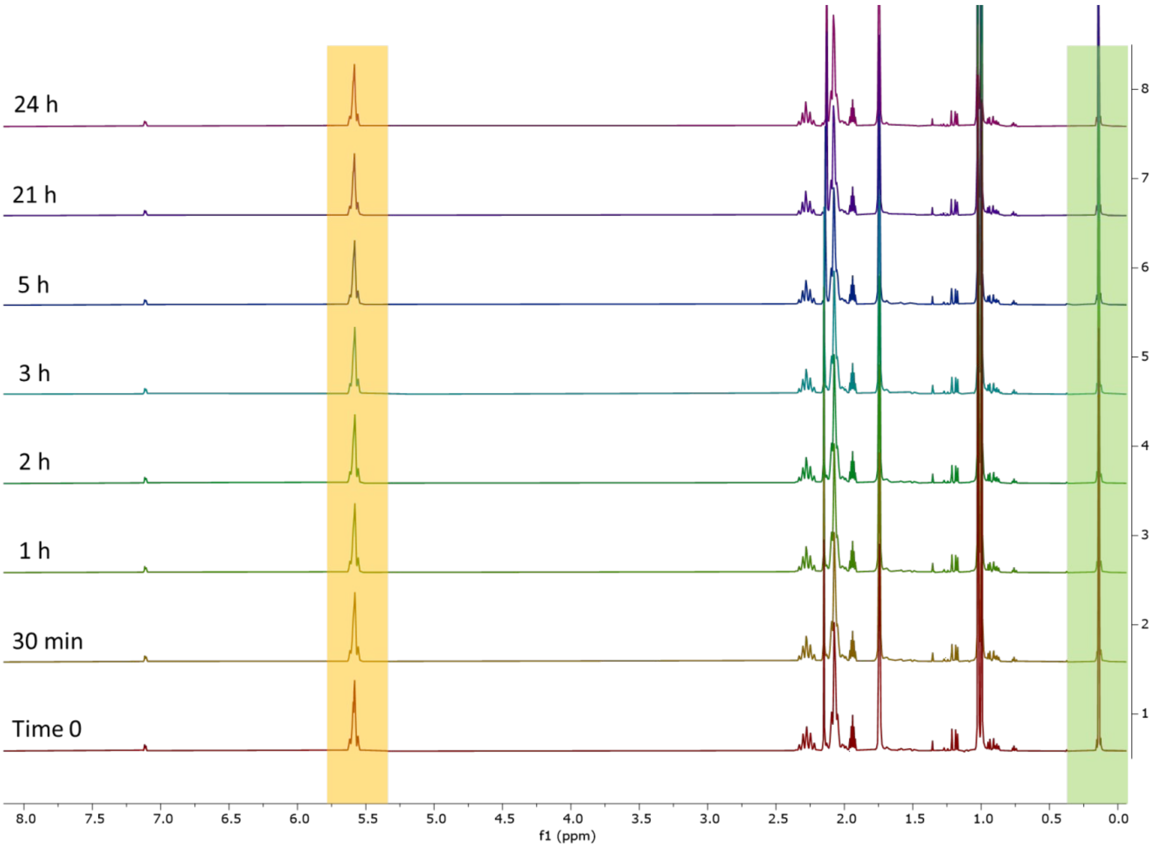

Figure S55. ^{1}H-NMR (CD_3CN, 250 MHz) spectra of a solution of 0.05 mmol α-terpinene and 0.005 mmol of hexamethylcyclotrisiloxane as internal standard in 0.5 mL of oxygen-equilibrated CD_3CN after the indicated irradiation times. The signal highlighted in yellow is due to the reagent and the signal of the reference is highlighted in green.

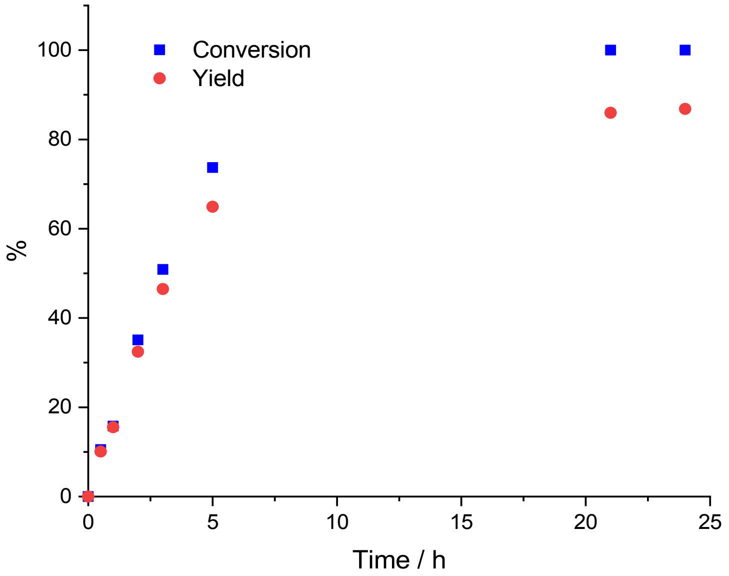

Figure S56. Plot of the conversion and yield as a function of time for the photocatalytic oxidation of α-terpinene upon irradiation at 632 nm in the presence of 1 mol% of (PPN)[Cr(^{tBuPh}BTP)$_2$] in oxygen-equilibrated CD_3CN.

Table S14. Substrate conversion and yield determined by integration of the characteristic peaks in the ^{1}H-NMR spectra by using hexamethylcyclotrisiloxane as internal standard. The last two columns refer to the control experiment in the absence of photocatalyst.

	**With photocatalyst**		**Control experiment**	
**Time / h**	**Conversion[a] / %**	**Yield[a] / %**	**Conversion[a] / %**	**Yield[a] / %**
0.5	10.5	10.0	0.9	0
1	15.8	15.5	0.9	0
2	35.1	32.5	0.9	0
3	50.9	46.5	0.9	0
5	73.9	64.9	1.9	0
21	>99	86.0	4.7	0
24	>99	86.8	4.7	0

[a] The expected error in the percentage value is ±0.1.

12 Photostability

12.1 Photostability in air-equilibrated solution

The photostability of (nBu$_4$N)[Cr(^{tBuPh}BTP)$_2$] was assessed both in air-equilibrated and degassed MeCN solutions.

A 90 µM solution of (nBu$_4$N)[Cr(^{tBuPh}BTP)$_2$] in 4 mL of air-equilibrated MeCN was introduced in a quartz cuvette. The sample was irradiated with a 405-nm laser (incident power: 0.3 W) while stirring. Absorption spectra were automatically detected at specific times (Fig. S57a).

The concentration of the sample was monitored by monitoring the absorbance value at 355 nm (at which the molar absorption coefficient is 5,500 M^{-1}cm^{-1}. By dividing the final concentration by the value of the initial concentration, the plot shown in Fig. S57b was obtained. After 10 h the photodegradation slows down.

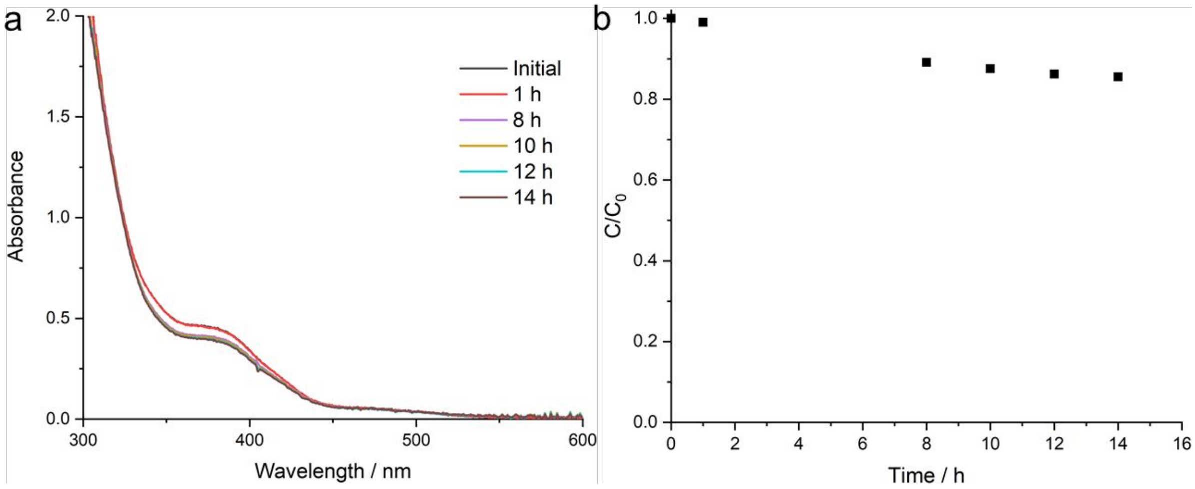

Figure S57. (a) Absorption spectra of an air-equilibrated 90 µM solution of (nBu$_4$N)[Cr(^{tBuPh}BTP)$_2$] in MeCN at different irradiation times with a 405-nm laser; (b) relative concentration at different irradiation times.

The photodegradation quantum yield was determined individual irradiation time t according to the formula:

$$\Phi_{deg}(t) = \frac{n_{reag\ deg}(t)}{n_{abs\ photons}(t)} \qquad \text{(S18)}$$

Where $n_{(reag\ deg)}(t)$ indicates the molar quantity of degraded Cr^{III} complex at the time t and $n_{(abs\ photons)}(t)$ the mE of absorbed photons at the time t.

The number of absorbed photons was determined based on the absorbance at 405 nm (~0.28 at every time, meaning that 47% of the incident photons are absorbed at that wavelength) and based on the number of incident photons.

The irradiation intensity of 0.3 W at 405 nm (i.e., 4.90×10^{-19} J/photon) corresponds to 3.64 mE/h. The resulting absorbed flux is 1.71 mE/h.

The obtained photodegradation quantum yield under air-equilibrated conditions is (2.3 ± 0.3)×10^{-4}%.

12.2 Photostability under inert (Ar) atmosphere

A 95 µM solution of (nBu$_4$N)[Cr(^{tBuPh}BTP)$_2$] in 4 mL of MeCN was introduced in a quartz cuvette and degassed by flushing argon for 10 minutes. The sample was irradiated with a 405-nm laser (incident power: 0.3 W) while stirring. Absorption spectra were automatically detected at specific times (Fig. S58a).

The photodegradation quantum yield was determined in the same way as described above. In this case the absorbance of the sample at 405 nm was 0.3, meaning that the fraction of absorbed photons was 50% (i.e. 1.82 mE/h).

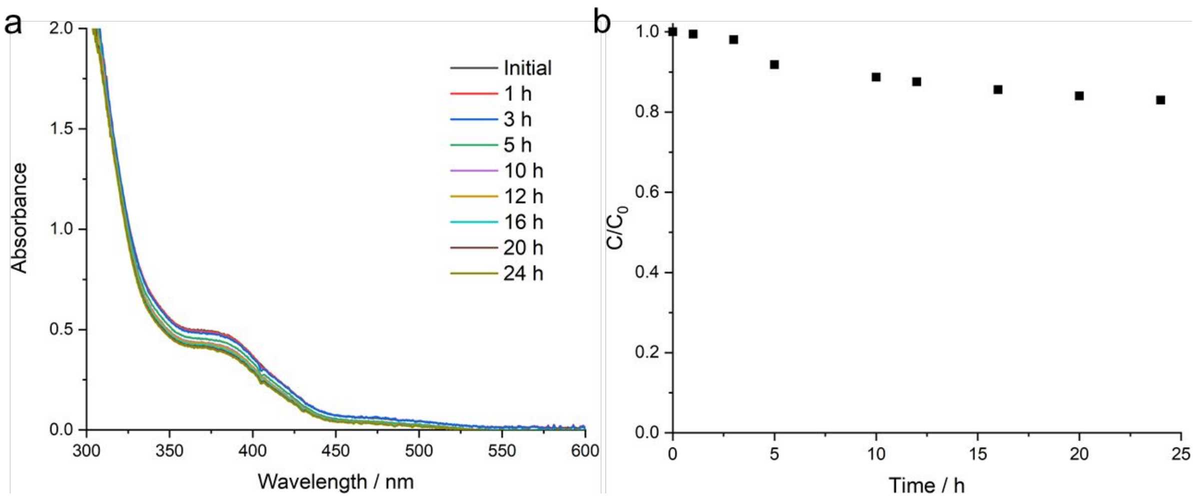

Figure S58. (a) Absorption spectra of an Ar-saturated 95 µM solution of (nBu$_4$N)[Cr(^{tBuPh}BTP)$_2$] in MeCN at different irradiation times with a 405-nm laser; (b) relative concentration at different irradiation times.

The obtained photodegradation quantum yield under air-equilibrated conditions is $(2.5 \pm 0.6)\times10^{-4}$% according to Eq. S18. This value is very close in agreement with the value obtained under oxygen-free conditions, indicating that oxygen is not causing the photo-decomposition.

13 $^2E/^2T_1$ excited-state lifetime dependence on the Cr^{III} concentration and the excitation power

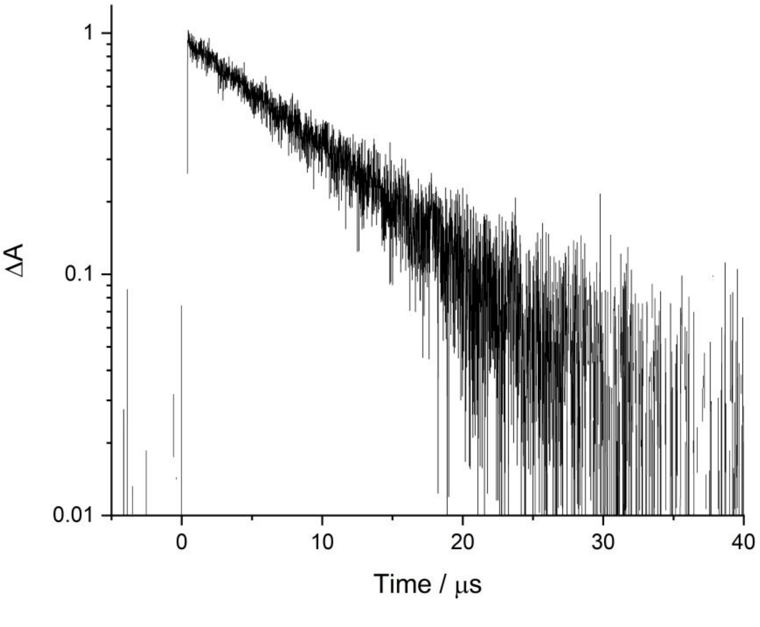

Figure S59. Excited state absorption decay (λ_{ex} = 532 nm, 38 mJ, 200 ns of integration time, detected at 680 nm, logarithmic scale, normalized) of 7.0 mM (nBu$_4$N)[Cr(^{tBuPh}BTP)$_2$] in deoxygenated MeCN.

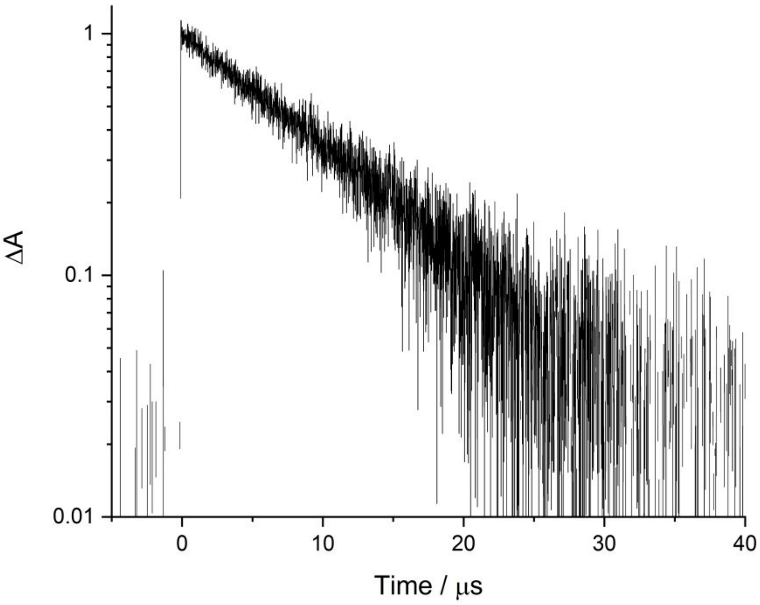

Figure S60. Excited state absorption decay (λ_{ex} = 532 nm, 55 mJ, 200 ns of integration time, detected at 680 nm, logarithmic scale, normalized) of 7.0 mM (nBu$_4$N)[Cr(^{tBuPh}BTP)$_2$] in deoxygenated MeCN.

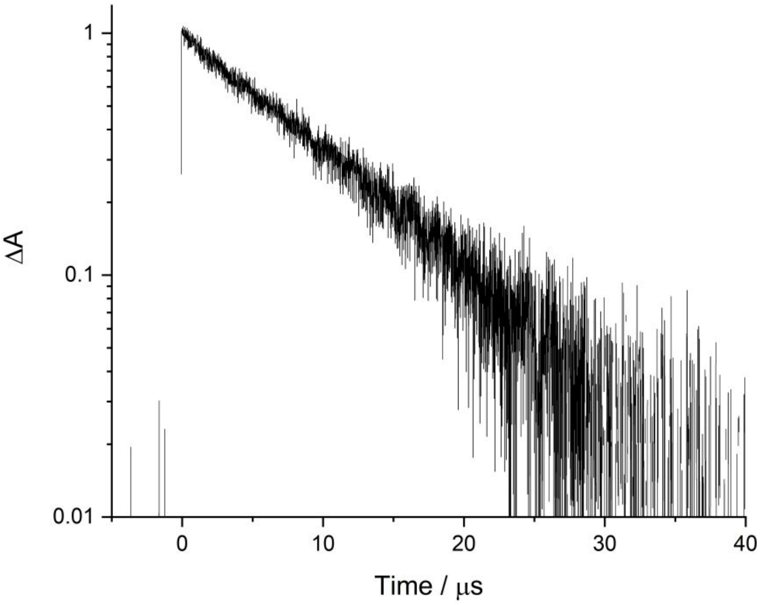

Figure S61. Excited state absorption decay (λ_{ex} = 532 nm, 80 mJ, 200 ns of integration time, detected at 680 nm, logarithmic scale, normalized) of 7.0 mM (nBu$_4$N)[Cr(^{tBuPh}BTP)$_2$] in deoxygenated MeCN.

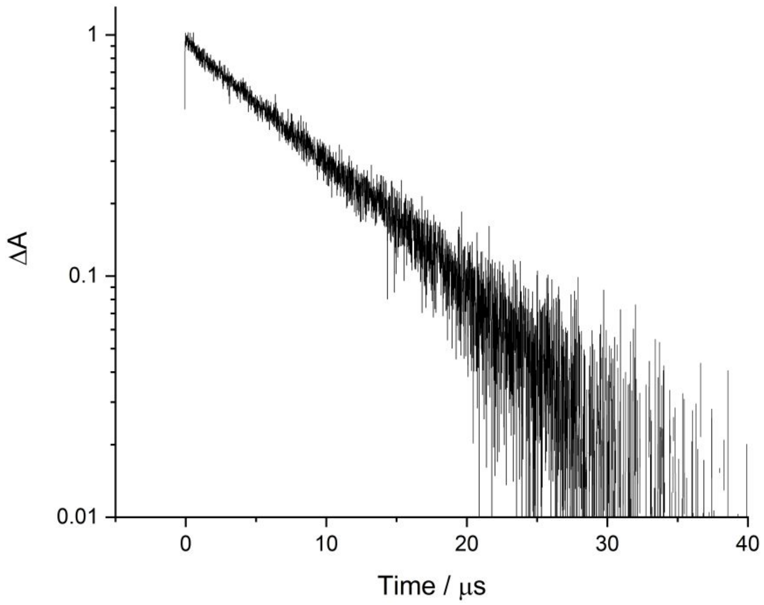

Figure S62. Excited state absorption decay (λ_{ex} = 532 nm, 100 mJ, 200 ns of integration time, detected at 680 nm, logarithmic scale, normalized) of 7.0 mM ($n$$Bu_4N$)[Cr(^{tBuPh}BTP)$_2$] in deoxygenated MeCN.

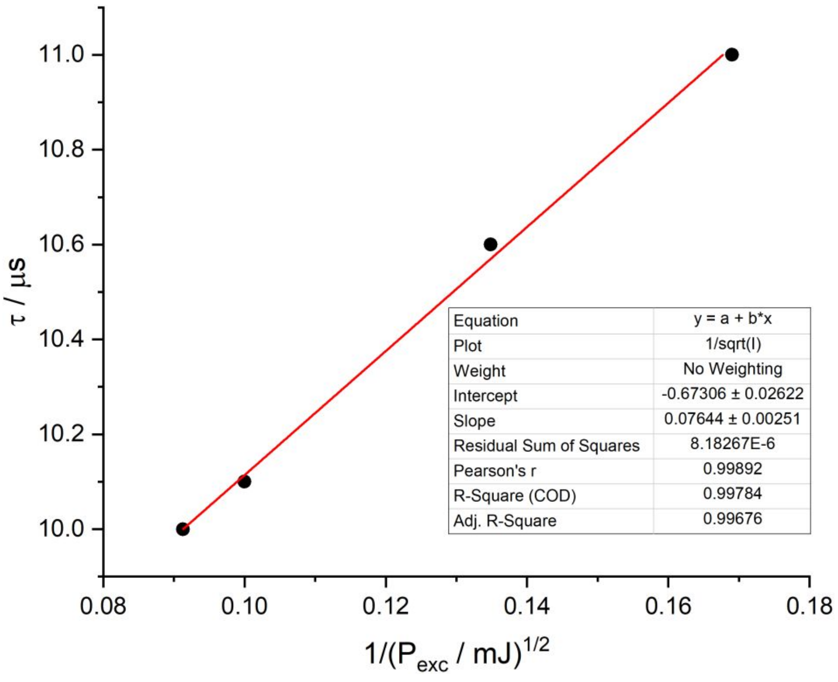

Figure S63. Dependence of the $^2E/^2T_1$ excited state lifetime of ($n$$Bu_4N$)[Cr(^{tBuPh}BTP)$_2$] (7.0 mM, Ar-equilibrated MeCN, monitoring the disappearance of the excited-state absorption at 680 nm) on the reciprocal of the square root of the excitation pulse energy (at 532 nm). The excitation pulse duration was 10 ns. The absorbance of the sample at the excitation wavelength was ca. 0.4. The solid red line is the result of a linear regression fit to the experimental data.

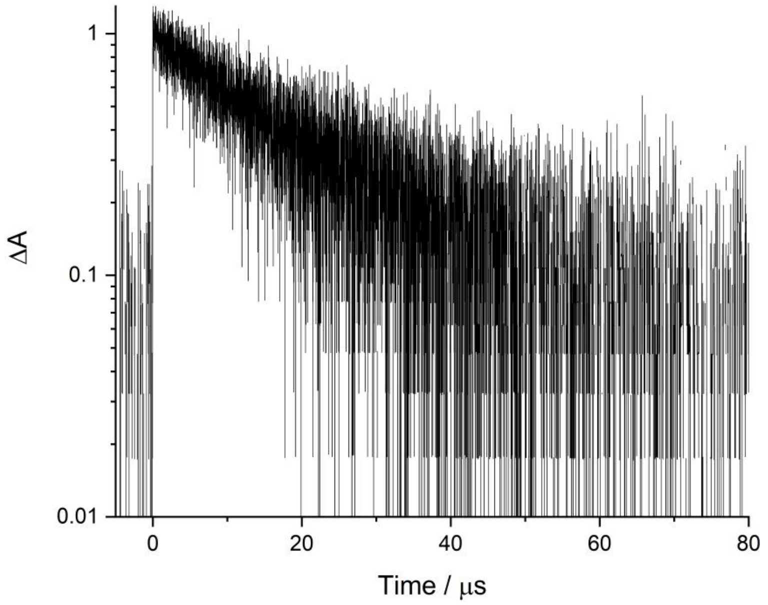

Figure S64. Excited state absorption decay (λ_{ex} = 355 nm, 15 mJ, 200 ns of integration time, detected at 680 nm, logarithmic scale, normalized) of 7.0 mM (nBu$_4$N)[Cr(^{tBuPh}BTP)$_2$] in deoxygenated MeCN.

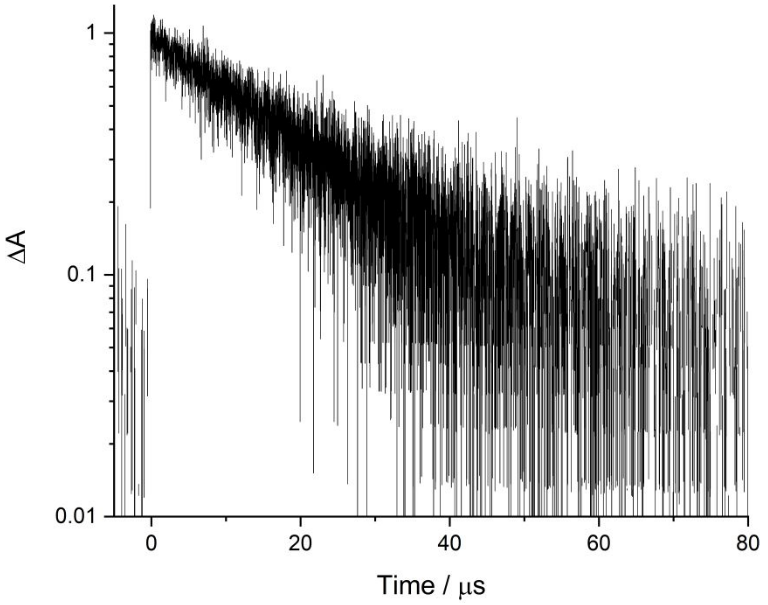

Figure S65. Excited state absorption decay (λ_{ex} = 355 nm, 20 mJ, 200 ns of integration time, detected at 680 nm, logarithmic scale, normalized) of 7.0 mM (nBu$_4$N)[Cr(^{tBuPh}BTP)$_2$] in deoxygenated MeCN.

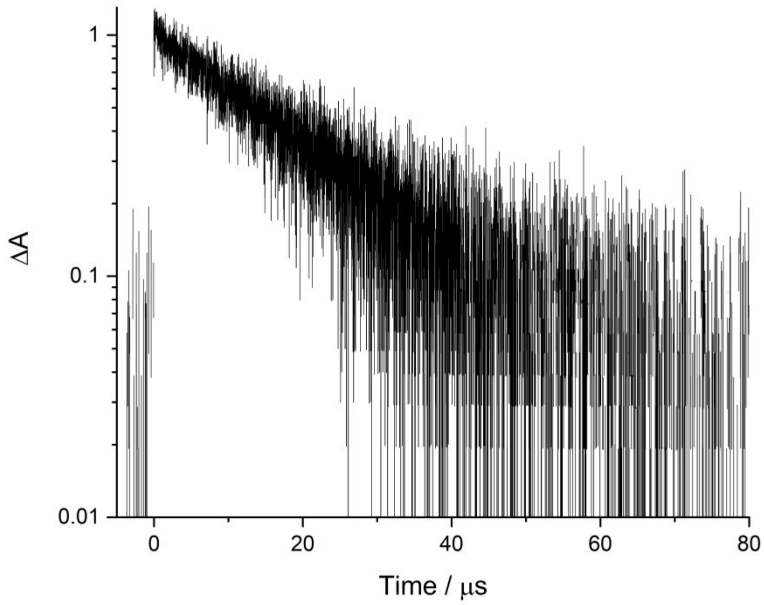

Figure S66. Excited state absorption decay (λ_{ex} = 355 nm, 50 mJ, 200 ns of integration time, detected at 680 nm, logarithmic scale, normalized) of 7.0 mM (nBu$_4$N)[Cr(^{tBuPh}BTP)$_2$] in deoxygenated MeCN.

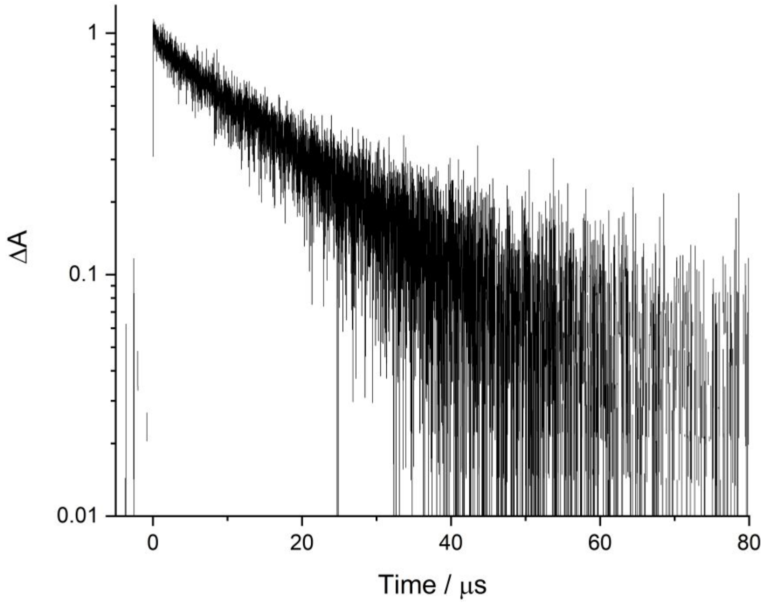

Figure S67. Excited state absorption decay (λ_{ex} = 355 nm, 60 mJ, 200 ns of integration time, detected at 680 nm, logarithmic scale, normalized) of 7.0 mM (nBu$_4$N)[Cr(^{tBuPh}BTP)$_2$] in deoxygenated MeCN.

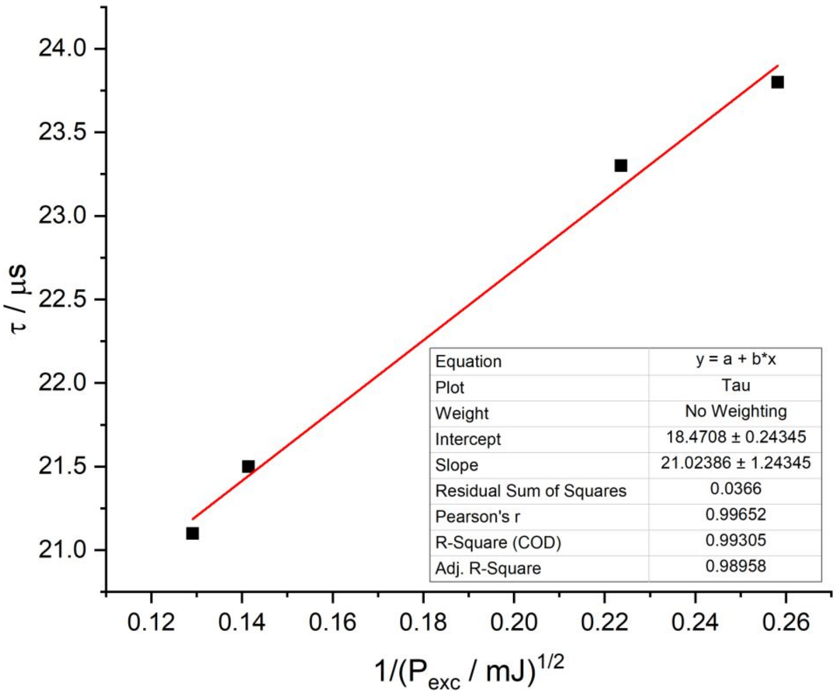

Figure S68. Dependence of the $^2E/^2T_1$ excited state lifetime of (nBu$_4$N)[Cr(^{tBuPh}BTP)$_2$] (0.1 mM, Ar-equilibrated MeCN, monitoring the disappearance of the excited-state absorption at 680 nm) on the reciprocal of the square root of the excitation pulse energy (at 355 nm). The excitation pulse duration was 10 ns. The absorbance of the sample at the excitation wavelength was ca. 0.4. The solid red line is the result of a linear regression fit to the experimental data.

The dependence of the $^2E/^2T_1$ excited state lifetime on the square root of the excitation power (P_{exc}) has its physical origin in a second-order process involving two $^2E/^2T_1$ excited Cr^{III} complexes as the following kinetic analysis demonstrates. Specifically, we consider here the population of the photoactive doublet excited state (here abbreviated simply as *D*) from an energetically higher-lying quartet state (*Q*), the depopulation of the doublet excited state to the ground state (*GS*), and an annihilation process involving two doublet excited states:

1) $Q \xrightarrow{k_1} D$

2) $D \xrightarrow{k_2} GS$

3) $D + D \xrightarrow{k_3} \ldots$

Where k_1 is the first order rate constant for the processes leading to the population of *D* upon intersystem-crossing from *Q*, k_2 describes the deactivation of the doublet excited state to the ground state, involving both radiative or nonradiative decays, and k_3 is a second order rate constant describing the annihilation process.

The kinetic equation describing the variation of the concentration of the doublet excited state over time is:

$$\frac{d[D]}{dt} = k_1[Q] - k_2[D] - 2k_3[D]^2 \qquad \text{(S19)}$$

Under steady-state conditions, the concentration of the doublet excited state is constant (applicable when considering only a very short period of time, i.e. within the excitation pulse duration), and the equation above reduces to:

$$k_1[Q] = k_2[D] + 2k_3[D]^2 \qquad \text{(S20)}$$

The presence of a fairly pure mono-exponential decay dependent on the excitation pulse energy, made us assume that the second order process is considerably faster than the first-order decay, *i.e.* $k_3[D]^2 \gg k_2[D]$, we obtain:

$$k_1[Q] \approx 2k_3[D]^2 \quad \text{(S21)}$$

The concentration of the quartet excited state is linearly dependent on the excitation power, and consequently it follows from this equation here that there is a linear dependency between the concentration of doublets and the square root of the excitation power.

Under the assumption that the annihilation process is much faster than the inherent $^2E/^2T_1$ excited state decay to the electronic ground state (see above), the observable $^2E/^2T_1$ excited state lifetime (τ_{obs}) can be expressed as follows:

$$\tau_{obs} = \frac{1}{k_3[D]} \quad \text{(S22)}$$

Since $k_3[D]$ linearly depends on the square root of the excitation power, the lifetime of the doublet is assumed to be linearly dependent on the reciprocal of the square root of the excitation power.

It is worth highlighting that all the measurements involving a decrease in the lifetime (namely, the Stern-Volmer experiments in the presence of oxygen and electron donors) were performed at low concentrations at low powers to avoid parasite processes of doublet quenching. *Id est*, for all the measurements, the initial lifetime τ_0 was 23 μs.

14 References

(1) Lou, D.; Yutronkie, N. J.; Oyarzabal, I.; Wang, L. F.; Adak, A.; Nadurata, V. L.; Diego, R.; Suturina, E. A.; Mailman, A.; Dechambenoit, P.; Rouzières, M.; Wilhelm, F.; Rogalev, A.; Bonhommeau, S.; Mathonière, C.; Clérac, R. Self-Assembled Tetranuclear Square Complex of Chromium(III) Bridged by Radical Pyrazine: A Molecular Model for Metal-Organic Magnets. *J. Am. Chem. Soc.* **2024**, *146*, 19649–19653.

(2) Mydlak, M.; Mauro, M.; Polo, F.; Felicetti, M.; Leonhardt, J.; Diener, G.; De Cola, L.; Strassert, C. A. Controlling Aggregation in Highly Emissive Pt(II) Complexes Bearing Tridentate Dianionic N^N^N Ligands. Synthesis, Photophysics, and Electroluminescence. *Chem. Mater.* **2011**, *23*, 3659–3667.

(3) Walden, M. T.; Yufit, D. S.; Williams, J. A. G. Luminescent Bis-Tridentate Iridium(III) Complexes: Overcoming the Undesirable Reactivity of Trans-Disposed Metallated Rings Using –N^N^N–Coordinating Bis(1,2,4-Triazolyl)Pyridine Ligands. *Inorg. Chim. Acta* **2022**, *532*, 120737.

(4) El Garah, M.; Sinn, S.; Dianat, A.; Santana-Bonilla, A.; Gutierrez, R.; De Cola, L.; Cuniberti, G.; Ciesielski, A.; Samorì, P. Discrete Polygonal Supramolecular Architectures of Isocytosine-Based Pt(II) Complexes at the Solution/Graphite Interface. *Chem. Commun.* **2016**, *52*, 11163–11166.

(5) Dolomanov, O. V.; Bourhis, L. J.; Gildea, R. .; Howard, J. A. K.; Puschmann, H. $OLEX_2$: A Complete Structure Solution, Refinement and Analysis Program. *J. Appl. Crystallogr.* **2009**, *42*, 339–341.

(6) Sheldrick, G. M. HELXT – Integrated Space-Group and Crystal_structure Determination. *Acta Crystallogr. Sect. A* **2015**, *A71*, 3–8.

(7) Sheldrick, G. M. Crystal Structure Refinement with SHELXL. *Acta Crystallogr. Sect. A* **2015**, *C71*, 3–8.

(8) Ketkaew, R.; Tantirungrotechai, Y.; Harding, P.; Chastanet, G.; Guionneau, P.; Marchivie, M.; Harding, D. J. OctaDist: A Tool for Calculating Distortion Parameters in Spin Crossover and Coordination Complexes. *Dalton Trans.* **2021**, *50*, 1086–1096.

(9) Dieterich, S.; Strähle, J. Zur Struktur Des Pentaazadienidions $[Tol\text{-}N{=}N\text{-}N\text{-}N{=}N\text{-}Tol]^-$. Synthese Und Kristallstruktur von Cs_2(18-Krone-6)$(TolN_5tol)_2$ Und $(NH_4)[Cr(NH_3)_6(H_2O)_4](TolN_5tol)_4$. *Zeitschrift für Naturforsch. B* **1993**, *48*, 1574–1580.

(10) Hauser, A.; Mäder, M.; Robinson, W. T.; Murugesan, R.; Ferguson, J. Electronic and Molecular Structure of $[Cr(bpy)_3]^{3+}$ (bpy = 2,2'-Bipyridine). *Inorg. Chem.* **1987**, *26*, 1331–1338.

(11) Jiménez, J. R.; Doistau, B.; Cruz, C. M.; Besnard, C.; Cuerva, J. M.; Campaña, A. G.; Piguet, C. Chiral Molecular Ruby $[Cr(dqp)^2]^{3+}$ with Long-Lived Circularly Polarized Luminescence. *J. Am. Chem. Soc.* **2019**, *141*, 13244–13252.

(12) Stein, L.; Wang, C.; Förster, C.; Resch-Genger, U.; Heinze, K. Bulky Ligands Protect Molecular Ruby from Oxygen Quenching. *Dalton Trans.* **2022**, *51*, 17664–17670.

(13) Wickramasinghe, W. A.; Bird, P. H.; Jamieson, M. A.; Serpone, N. Interligand Pockets in Polypyridyl Complexes. The Crystal and Molecular Structure of the $Cr(2,2',2''\text{-terpyridine})_2^{3+}$ Ion. *J. Chem. Soc., Chem. Commun.* **1979**, *18*, 798–800.

(14) Frisch, M. J.; Trucks, G. W.; Schlegel, H. B.; Scuseria, G. E.; Robb, M. A.; Cheeseman, J. R.; Scalmani, G.; Barone, V.; Petersson, G. A.; Nakatsuji, H.; Li, X.; Caricato, M.; Marenich, A. V.; Bloino, J.; Janesko, B. G.; Gomperts, R.; Mennucci, B.; Hratchian, H. P.; Al., E. *Gaussian 16, Revision C.01*. .J. Gaussian, Inc., Wallingford CT , GaussView 5.0. Wallingford, E.U.A.

(15) Lee, C.; Yang, W.; Parr, R. G. Development of the Colle-Salvetti Correlation-Energy Formula into a Functional of the Electron Density. *Phys. Rev. B* **1988**, *37*, 785–789.

(16) Becke, A. D. Density-functional Thermochemistry. III. The Role of Exact Exchange. *J. Chem. Phys.* **1993**, *98*, 5648–5652.

(17) Weigend, F.; Ahlrichs, R. Balanced Basis Sets of Split Valence, Triple Zeta Valence and Quadruple Zeta Valence Quality for H to Rn: Design and Assessment of Accuracy. *Phys. Chem. Chem. Phys.* **2005**, *7*, 3297–3305.

(18) Lu, T.; Chen, F. Multiwfn: A Multifunctional Wavefunction Analyzer. *J. Comput. Chem.* **2012**, *33*, 580–592.

(19) Version 1.8 build 682. *Chemcraft - graphical software for visualization of quantum chemistry computations.* https://www.chemcraftprog.com.

(20) Atkins, P. W.; Overton, T. L.; Rourke, J. P.; Weller, M. T.; Armstrong, F. A. *Shriver and Atkins' Inorganic Chemistry*; Press, O. U., Ed.; 2000.

(21) Yarranton, J. T.; McCusker, J. K. Ligand-Field Spectroscopy of Co(III) Complexes and the Development of a Spectrochemical Series for Low-Spin d^6 Charge-Transfer Chromophores. *J. Am. Chem. Soc.* **2022**, *144*, 12488–12500.

(22) Sinha, N.; Jiménez, J. R.; Pfund, B.; Prescimone, A.; Piguet, C.; Wenger, O. S. A Near-Infrared-II Emissive Chromium(III) Complex. *Angew. Chem., Int. Ed.* **2021**, *60*, 23722–23728.

(23) Sinha, N.; Yaltseva, P.; Wenger, O. S. The Nephelauxetic Effect Becomes an Important Design Factor for Photoactive First-Row Transition Metal Complexes. *Angew. Chem., Int. Ed.* **2023**, *62*, e202303864.

(24) Otto, S.; Dorn, M.; Förster, C.; Bauer, M.; Seitz, M.; Heinze, K. Understanding and Exploiting Long-Lived near-Infrared Emission of a Molecular Ruby. *Coord. Chem. Rev.* **2018**, *359*, 102–111.

(25) Sawicka, N.; Craze, C. J.; Horton, P. N.; Coles, S. J.; Richards, E.; Pope, S. J. A. Long-Lived, near-IR Emission from Cr(III) under Ambient Conditions. *Chem. Commun.* **2022**, *58*, 5733–5736.

(26) Stein, L.; Boden, P.; Naumann, R.; Förster, C.; Niedner-Schatteburg, G.; Heinze, K. The Overlooked NIR Luminescence of $Cr(ppy)_3$. *Chem. Commun.* **2022**, *58*, 3701–3704.

(27) Cheng, Y.; Yang, Q.; He, J.; Zou, W.; Liao, K.; Chang, X.; Zou, C.; Lu, W. The Energy Gap Law for NIR-Phosphorescent Cr(III) Complexes. *Dalton Trans.* **2022**, *52*, 2561–2565.

(28) Gowda, A. S.; Petersen, J. L.; Milsmann, C. Redox Chemistry of Bis(Pyrrolyl)Pyridine Chromium and Molybdenum Complexes: An Experimental and Density Functional Theoretical Study. *Inorg. Chem.* **2018**, *57*, 1919–1934.

(29) Crosby, G. A.; Demas, J. N. Measurement of Photoluminescence Quantum Yields. Review. *J. Phys. Chem.* **1971**, *75*, 991–1024.

(30) Montalti, M.; Credi, A.; Prodi, L.; Gandolfi, M. T. *Handbook of Photochemistry*, 3 ed.; Taylor & Francis, 2006.

(31) Lakowicz, J. R. *Principles of Fluorescence Spectroscopy*, 3rd ed.; Springer US: Boston, MA, 2006.

(32) Wilkinson, F.; Helman, W. P.; Ross, A. B. Quantum Yields for the Photosensitized Formation of the Lowest Electronically Excited Singlet State of Molecular Oxygen in Solution. *J. Phys. Chem. Ref. Data* **1993**, *22*, 113–262.

(33) Balzani, V.; Ceroni, P.; Juris, A. *Photochemistry and Photophysics: Concepts, Research, Applications*, Second Edi.; Wiley, Ed.; 2024.

(34) Broggi, J.; Terme, T.; Vanelle, P. Organic Electron Donors as Powerful Single-Electron

Reducing Agents in Organic Synthesis. *Angew. Chem., Int. Ed.* **2014**, *53*, 384–413.

(35) Shukla, J.; Kumar, S.; Rustam; Mukhopadhyay, P. Synthesis of Stable, High-SOMO Zwitterionic Radicals: Enabling Intermolecular Electron Transfer between Naphthalenediimides. *Org. Lett.* **2020**, *16*, 6229–6233.

(36) Pellegrin, Y.; Odobel, F. Sacrificial Electron Donor Reagents for Solar Fuel Production. *Comptes Rendus. Chim.* **2017**, *20*, 283–295.

(37) Lenshina, N. A.; Arsenyev, M. V.; Fagin, A. A.; Bogdanov, A. V.; Chesnokov, S. A. Photoinitiating Systems Based on 1-Hexadecylisatin Derivatives. *Russ. Chem. Bull.* **2023**, *72*, 1737–1745.

(38) Wang, C.; Li, H.; Bürgin, T. H.; Wenger, O. S. Cage Escape Governs Photoredox Reaction Rates and Quantum Yields. *Nat. Chem.* **2024**, *16*, 1151–1159.

(39) Cheng, H.; Lam, T.-L.; Liu, Y.; Tang, Z.; Che, C.-M. Photoinduced Hydroarylation and Cyclization of Alkenes with Luminescent Platinum(II) Complexes. *Angew. Chem., Int. Ed.* **2020**, *60*, 1383–1389.

(40) Bürgin, T. H.; Glaser, F.; Wenger, O. S. Shedding Light on the Oxidizing Properties of Spin-Flip Excited States in a CrIII Polypyridine Complex and Their Use in Photoredox Catalysis. *J. Am. Chem. Soc.* **2022**, *144*, 14181–14194.

(41) Balzani, V.; Bolletta, F.; Scandola, F. Vertical and "Nonvertical" Energy Transfer Processes. A General Classical Treatment. *J. Am. Chem. Soc.* **1980**, *102*, 2152–2163.

(42) Rehm, D.; Weller, A. Kinetics of Fluorescence Quenching by Electron and H-Atom Transfer. *Isr. J. Chem.* **1970**, *8*, 259–271.

(43) Rosspeintner, A.; Angulo, G.; Vauthey, E. Bimolecular Photoinduced Electron Transfer beyond the Diffusion Limit: The Rehm-Weller Experiment Revisited with Femtosecond Time Resolution. *J. Am. Chem. Soc.* **2014**, *136*, 2026–2032.

(44) Prier, C. K.; Rankic, D. A.; MacMillan, D. W. C. Visible Light Photoredox Catalysis with Transition Metal Complexes: Applications in Organic Synthesis. *Chem. Rev.* **2013**, *113*, 5322–5363.

(45) Schenck, G. O. Probleme Praparativer Photochemie. *Angew. Chem.* **1952**, *64*, 12–23.
